# COMPASS: An Open-Source, General-Purpose Software Toolkit for Computational Psychiatry

**DOI:** 10.3389/fnins.2018.00957

**Published:** 2019-01-11

**Authors:** Ali Yousefi, Angelique C. Paulk, Ishita Basu, Jonathan L. Mirsky, Darin D. Dougherty, Emad N. Eskandar, Uri T. Eden, Alik S. Widge

**Affiliations:** ^1^Department of Psychiatry, Massachusetts General Hospital and Harvard Medical School, Boston, MA, United States; ^2^Department of Mathematics and Statistics, Boston University, Boston, MA, United States; ^3^Department of Neurology, Massachusetts General Hospital and Harvard Medical School, Boston, MA, United States; ^4^Department of Neurosurgery, Massachusetts General Hospital and Harvard Medical School, Boston, MA, United States

**Keywords:** computational psychiatry, mathematical behavioral analysis, computational methods, state-space modeling, open source software, cognitive neuroscience

## Abstract

Mathematical modeling of behavior during a psychophysical task, referred to as “computational psychiatry,” could greatly improve our understanding of mental disorders. One barrier to the broader adoption of computational methods, is that they often require advanced statistical modeling and mathematical skills. Biological and behavioral signals often show skewed or non-Gaussian distributions, and very few toolboxes and analytical platforms are capable of processing such signal categories. We developed the Computational Psychiatry Adaptive State-Space (COMPASS) toolbox, an open-source MATLAB-based software package. This toolbox is easy to use and capable of integrating signals with a variety of distributions. COMPASS has the tools to process signals with continuous-valued and binary measurements, or signals with incomplete—missing or censored—measurements, which makes it well-suited for processing those signals captured during a psychophysical task. After specifying a few parameters in a small set of user-friendly functions, COMPASS allows users to efficiently apply a wide range of computational behavioral models. The model output can be analyzed as an experimental outcome or used as a regressor for neural data and can also be tested using the goodness-of-fit measurement. Here, we demonstrate that COMPASS can replicate two computational behavioral analyses from different groups. COMPASS replicates and can slightly improve on the original modeling results. We also demonstrate the use of COMPASS application in a censored-data problem and compare its performance result with naïve estimation methods. This flexible, general-purpose toolkit should accelerate the use of computational modeling in psychiatric neuroscience.

## Introduction

There is a growing need for advanced computational methods within psychiatric neuroscience (Wang and Krystal, 2014; Paulus et al., 2016; Redish and Gordon, 2016). Existing investigational techniques have not revealed the neural mechanisms of complex behavioral disorders. This may in part be because behavior is difficult to measure objectively. Newer work seeks to explain complex behaviors in terms of relatively low-dimensional parametric models, where each parameter maps to a psychological or brain circuit construct (Wang and Krystal, 2014; Paulus et al., 2016; Widge et al., 2017). Such models have been used to quantify measurements of psychiatric disease processes, relative to healthy subjects, or to design experiments based on theoretical predictions (Voon and Fox, 2007; Gold et al., 2012; Anticevic et al., 2015; Widge et al., 2017). Computational models are also expected to improve the reliability, utility, and predictability of human behavior in specific cognitive tasks. For example, investigations in psychiatric neuroscience usually classify subjects through categorical diagnoses and a subjective rating scale. This, in turn, leads to poor signal-to-noise ratios (SNR) and difficulty in identifying reliable neural biomarkers of psychiatric illness (Insel et al., 2010; Widge et al., 2016, 2017). A more reliable approach may be to classify patients based on models of (ab)normal functioning. For instance, both patients and controls could perform the same standard psychophysical task, and patients' degree of abnormality could be quantified based on the parameters of a model fitted to their task behavior. Such models' output(s) can become independent (regressor) variables in neuro-imaging or electrophysiologic analyses (O'Doherty et al., 2007; Cavanagh, 2015; Webb et al., 2016), potentially reducing inter-subject variability and improving the SNR. Computational modeling also provides a framework for another major goal of psychiatric neuroscience: the identification of cross-diagnostic phenotypes and the circuits sub-serving those phenotypes (Insel et al., 2010; Anticevic et al., 2012; Huys et al., 2016; Redish and Gordon, 2016; Sweis et al., 2018).

A significant challenge is that modeling the analyses of psychophysical task behavior is often performed using custom written programming packages, often in the framework of a specific underlying theoretical construct. The behavior is then formalized as a function, in a system of parameterized equations, finally identifying those parameters consistent with each experimental subject's observed data. Because each laboratory does this differently, often using custom-developed computer programs optimized for the modeling approach at hand, not only could the resulting programs be inappropriate to analyze slightly different datasets but comparing models and their parameter development is nearly impossible across laboratories. Peer reviewers who are not modeling/programming experts themselves are not necessarily able to assess whether models have been correctly implemented, particularly if the code, the data, and all parameters are not openly shared (Blackford, 2017). Many researchers also do not have the mathematical/computational expertise to design such modeling systems *de novo*. Similar problems arose in the early days of neuro-imaging and have been ameliorated at least in part by the development of freely available analysis packages that make it easier to apply best practices (Oostenveld et al., 2011; Ashburner, 2012; Cox, 2012; Fischl, 2012; Gramfort et al., 2014; Ahn et al., 2017; Blackford, 2017; Poldrack et al., 2017).

Considerable efforts have been made to standardize the modeling of complex behavior using analysis packages, which can model underlying features, and both derive and predict behavior such as hBayesDM (Ahn et al., 2017) and KFAS (Helske, 2016) using R, VBA (Daunizeau et al., 2014) and TAPAS (Mathys et al., 2011; Kasper et al., 2017) sing MATLAB and HDDM (Wiecki et al., 2013) using Python. The available packages, however, do not work well with multiple behavioral outputs (e.g., reaction times and choices), or with data that naturally follow a specific type of non-normal distribution. These packages also do not fully handle missing and censored information in datasets (Shih, 2002).

Here, we present a general-purpose, open-source toolbox to apply to a wide variety of computational models and to an equally wide variety of behavioral data, following the precept that not only can the data be treated equally, but the parameter space and pipeline for deriving models of state, including predictive models of behavior, can be standardized. COMPASS is based on the state-space formalism, which assumes that behavior is influenced both by the parameters of individual task trials and by an underlying “cognitive state” that varies smoothly from trial to trial. This framework has successfully modeled behavior and neural activity in many contexts (Eden et al., 2004; Prerau et al., 2009; Yousefi et al., 2015; Widge et al., 2017), and applies to the general concept that psychiatric symptoms arise from the disruption of basic underlying cognitive processes. Continuous (reaction times, physiologic measurements), binary (correct/incorrect, yes/no choices), and multinomial (learning of multiple stimulus-response contingencies) behavioral outputs can all be integrated into models, making the toolbox applicable to many laboratory tasks. To increase the applicability to “real world” data, COMPASS includes methods we recently developed to more optimally handle missing observations in these computational approaches (Yousefi et al., 2017a).

A distinct utility of COMPASS is its capability of processing data with non-Gaussian and mixed distributions. This assumption contrasts with the hypothesis of a Gaussian observation process—or additive Gaussian noise—widely adopted in the development of the aforementioned analytical tools (Bono et al., 2017). Though the Gaussian assumption is usually the first choice in the analysis of almost any neural and behavioral data, it might present a less accurate hypothesis in the analysis of many signal categories (Delucchi and Bostrom, 2004; Paninski et al., 2010; Limpert and Stahel, 2011, 2017). Mis-specifying the distribution will reduce the statistical power of the analysis and may cause accurate results to be overlooked (a Type II error) (Ghasemi and Zahediasl, 2012). For instance, a Gaussian distribution is an improper choice for characterizing reaction times and decision choice signals commonly recorded in behavioral experiments (Prerau et al., 2009). Other examples are survival data or neural spiking signals, which have distributions that significantly deviate from normality (Perkel et al., 1967; Mudholkar et al., 1996; Ratcliff and Smith, 2004). COMPASS provides the function to build distributions for data which are better described by Bernoulli, Gamma or a mixture of these distributions. It also has the tools to characterize point-process observation processes making COMPASS suitable for analysis of spiking data (Eden et al., 2004; Ratcliff and Van Dongen, 2011). Besides these functions, COMPASS is capable of processing incomplete—censored—data in a more optimal manner. Incomplete data are often obligatory as a function of an experiment design, justifying the need for analytical tools capable of processing these types of data (Yousefi et al., 2017a). For example, in the continuous-performance test (CPT), which is widely used to assess sustained and selective attention, the inter-trial interval is limited to force continuous, rapid decision making (Riccio et al., 2002). This often leads to missed responses, only some of which are deliberate omissions. Given these utilities of COMPASS with its ease of use, we think it aptly fulfills the need of behavioral experimentalists, computational psychiatrists as well as neuroscientists, in providing analytical tools with more versatility and flexible functions to be used in data analysis.

We first provide a general overview of COMPASS, then demonstrate its application on two examples of associative learning behavior from previously published literature. Besides these examples, we also demonstrate the application of COMPASS in a censored-data problem. In prior work, we showed how an early version of this toolbox could model reaction times in conflict tasks (Widge et al., 2017; Yousefi et al., 2017a). These examples illustrate the flexibility and generality of our approach. As a further illustration, the Supplementary Material shows the process of building a new model for a hypothetical decision task and provides further discuss the toolbox's model selection and parameter estimation. Finally, a detailed user manual and code are available at https://github.com/Eden-Kramer-Lab/COMPASS. In the toolbox manual and its GitHub repository, we also provide further modeling examples to describe its utilities in the analysis of diverse forms of behavioral and neural data. In the toolbox manual, we also discuss how to interpret and compare modeling results and more importantly, we provide multiple examples to help researchers to easily build their own analytical modeling using COMPASS.

## Overview of the State-space Toolbox Approach

**Figure 1:**
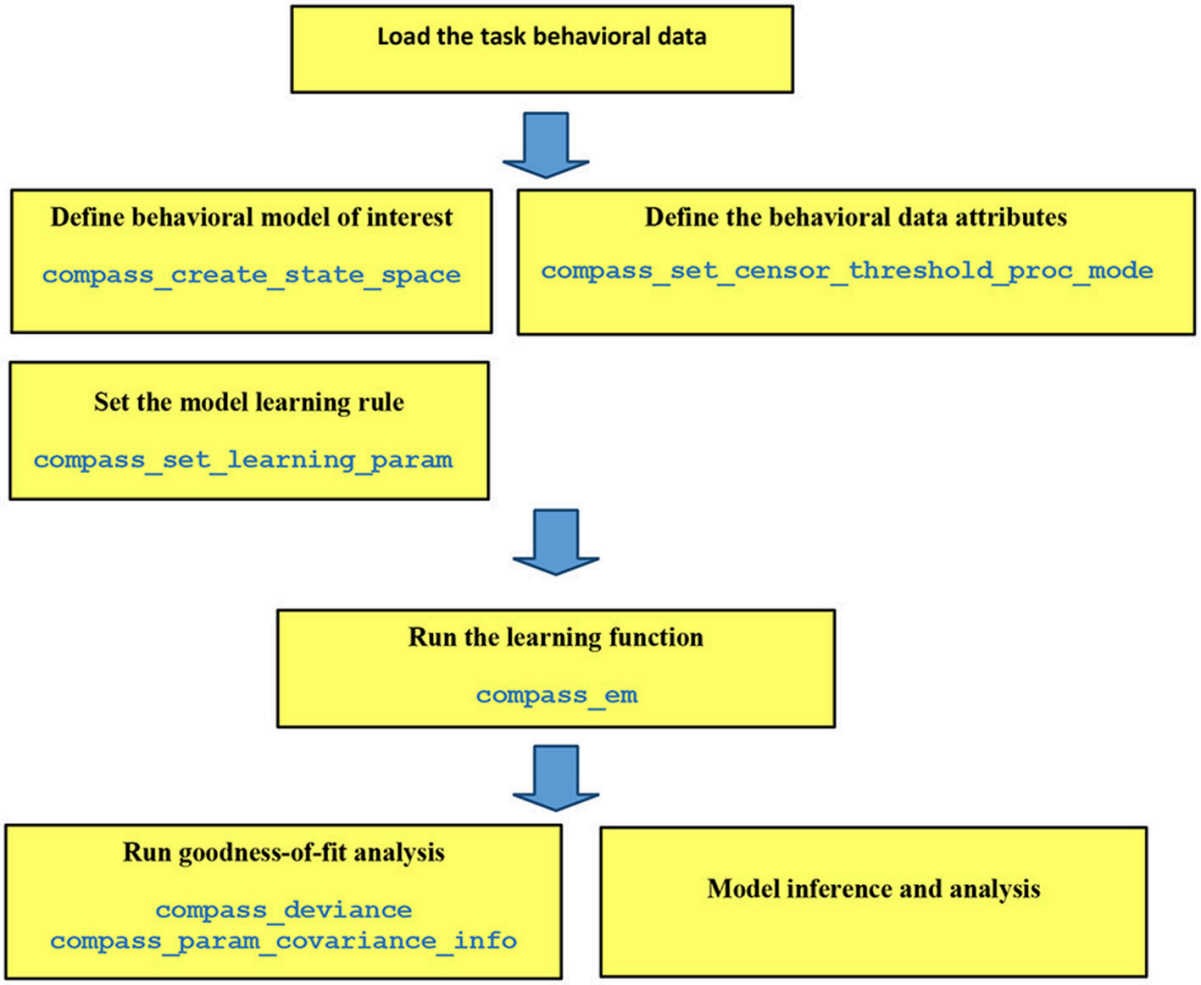
Pipeline of data analysis in COMPASS. The user manual at (https://github.com/Eden-Kramer-Lab/COMPASS/blob/master/compass_supplement_oct31_2018.pdf) describes each of these functions and how to use them.

Each step of COMPASS analysis pipeline is implemented as a high-level function in MATLAB (MathWorks, Natick, MA; Figure 1). Because these steps are separated, they can be configured to explore multiple models on a pilot dataset and determine which fits best before proceeding to a hypothesis-driven analyses. The core assumption is that behavior is driven by a multivariate cognitive state *X*, which varies over time according to its inherent dynamics and exogenous inputs:

(1) $$\begin{array}{r} f_{X_{k+1}|X_{k}}\sim \;N\left(AX_{k}+BU_{k},Q\right) \end{array}$$

where, *N* represents multivariate normal distribution with mean *AX*_*k*_+*BU*_*k*_ and covariance *Q*. That is, at time/trial *k*, the state *X*_*k*_ will evolve to its next value *X*_*k*+1_ based on innate transition dynamics *A* and its responsiveness *B* to an “input” *U*_*k*_. That evolution is driven by Gaussian noise with a covariance equal to *Q*. The parameters for this model (*A, B, Q*) can be assigned by the investigator or inferred from the behavioral data. For instance, by assigning *Q* to have small diagonal elements and *A* to be a diagonal matrix with elements close to 1, we would obtain a state with components independent of each other and which will change very little trial-to-trial unless acted upon by *U*_*k*_. This might model a more “trait-like” process. Making *Q* elements larger would favor a process that changes substantially during an experiment and would be more “state-like.” The input U_*k*_ may represent anything that would impact a subject's performance, including features of the current trial, the outcomes of past trials (e.g., a running total of rewards earned, or punishments delivered), or the presence/absence of an external manipulation such as a drug or neural stimulation.

We cannot directly observe *X*_*k*_, but we observe its effects on the set of task-related behaviors *Y*_*k*_, which again may include non-conscious “behaviors” such as a physiologic response. This “observation process” follows a parametric distribution *g*:

(2) $$\begin{array}{r} f_{Y_{k}|X_{k}}\sim \text{g}\left(X_{k},H_{k},I_{k},\theta \right) \end{array}$$

where, g defines the conditional distribution of *Y*_*k*_ as a function of *X*_*k*_, *H*_*k*_, *I*_*k*_, and θ. *H*_*k*_ represents the past history of the observations up to time *k*, and *I*_*k*_, like *U*_*k*_, may encode any other variables that can influence the behavior *Y*_*k*_ without having a persistent effect on the internal cognitive state *X*_*k*_. An example might be a conflict decision-making task, where trials with higher conflict/difficulty increase reaction times *Y*, but do not change the patient's underlying decisional bias *X* (Yousefi et al., 2015). Many possible *Y* are not normally distributed. Binary choices usually follow a Bernoulli distribution, and both reaction times and physiologic outputs often follow a gamma or log-normal distribution (Prerau et al., 2009; Palmer et al., 2011; Yousefi et al., 2015). Thus, g builds the relationship between *Y* and other covariates, (*X*_*k*_, *H*_*k*_, *I*_*k*_), using these distribution functions. COMPASS observation model thus allows us to model *Y* using a wide range of parametric distributions *g*, with parameters given by θ. We assume that *g* and θ do not vary trial-to-trial, and thus θ can be estimated directly from the data as with *A, B*, and *Q*. Further, *Y* may be multivariate, as in decision-making tasks where both the decision and the time needed to reach it may be experimentally relevant. In that situation, each element of the vector *Y*_*k*_ may have its own distribution, described by a *g*/θ pair.

COMPASS utilizes maximum likelihood estimation (MLE) in the model inference and cognitive state estimation. Using COMPASS, we not only build the model of interest but also estimate its free parameters and underlying state variable(s). The model free parameters are generally a subset of (θ, *A, B, Q*) parameters, which characterizes the state transition and observation processes. COMPASS utilizes an expectation-maximization algorithm (EM) in its estimation of the model parameters and provides the posterior estimation of *X*_*k*_ (Dempster et al., 1977; Smith and Brown, 2003; Widge et al., 2017). Appendix A in the Supplementary Material discusses COMPASS inference steps in detail.

Choosing a model and assessing the goodness-of-fit of that model are critical components of any statistical analysis. Therefore, the toolbox provides a collection of different goodness-of-fit methods to help identify and refine models of the data. These include methods based on the covariance of the estimated parameters and the model deviance/likelihood. The online manual includes an example of those assessments for one of the task examples given below.

Another important analysis issue relates to the common experimental problem of missing data (Little and Rubin, 2014; Yousefi et al., 2017a). Some data may be missing at random (MAR), such that knowing a given datum is missing provides no new information. For example, response recording devices are subject to noise that may obscure some trials, such as muscle artifact in an EEG or sensor failure for skin conductance. In such cases, trials with missing data are often removed prior to analysis. In other cases, features of the experiment influence the probability of data being missed. We do not identify these data as censored or missing at random. For example, in trial-based behavioral studies, subjects often fail to respond on individual trials within some designated time window, and this may be worse in patients taking psychotropic medications that can slow processing. In such cases, it is inadvisable to simply remove trials with missing data since these trials provide information about behavior. With censored reaction time (RT) data, we know that the subject's RT was larger than a specified threshold, which may affect the probability of a correct decision (Yousefi et al., 2017a). When fitting a model to COMPASS, each observation in the vector *Y* may be marked as observed, missing at random, or censored. COMPASS then incorporates this information in its state estimation and model identification processes, using the algorithms described in Yousefi et al. (2017a).

## Example 1: Multivariate Associative Learning

Associative learning tasks are one of the most common models used to assess psychiatric deficits (O'Doherty et al., 2007; Diwadkar et al., 2008; Limpert and Stahel, 2011; Wang and Krystal, 2014) and have been well-described using state-space models. In tasks where subjects must learn multiple associations simultaneously (Williams and Eskandar, 2006; Katnani et al., 2016), Prerau and colleagues described a method for inferring a single “learning state” (Prerau et al., 2009). In this task, a participant learns the association between four different scenes and targets; the participant is expected to learn this association and pick the correct target within a specific time window. The learning state variable estimates how well the overall set of associations has been learned, optimally integrating performance over all available stimuli (Prerau et al., 2009). The Prerau method also infers learning from both correct and incorrect choices and reaction times (RT), maximizing the information extracted from the available data (Prerau et al., 2009; Coleman et al., 2011).

**Figure 2:**
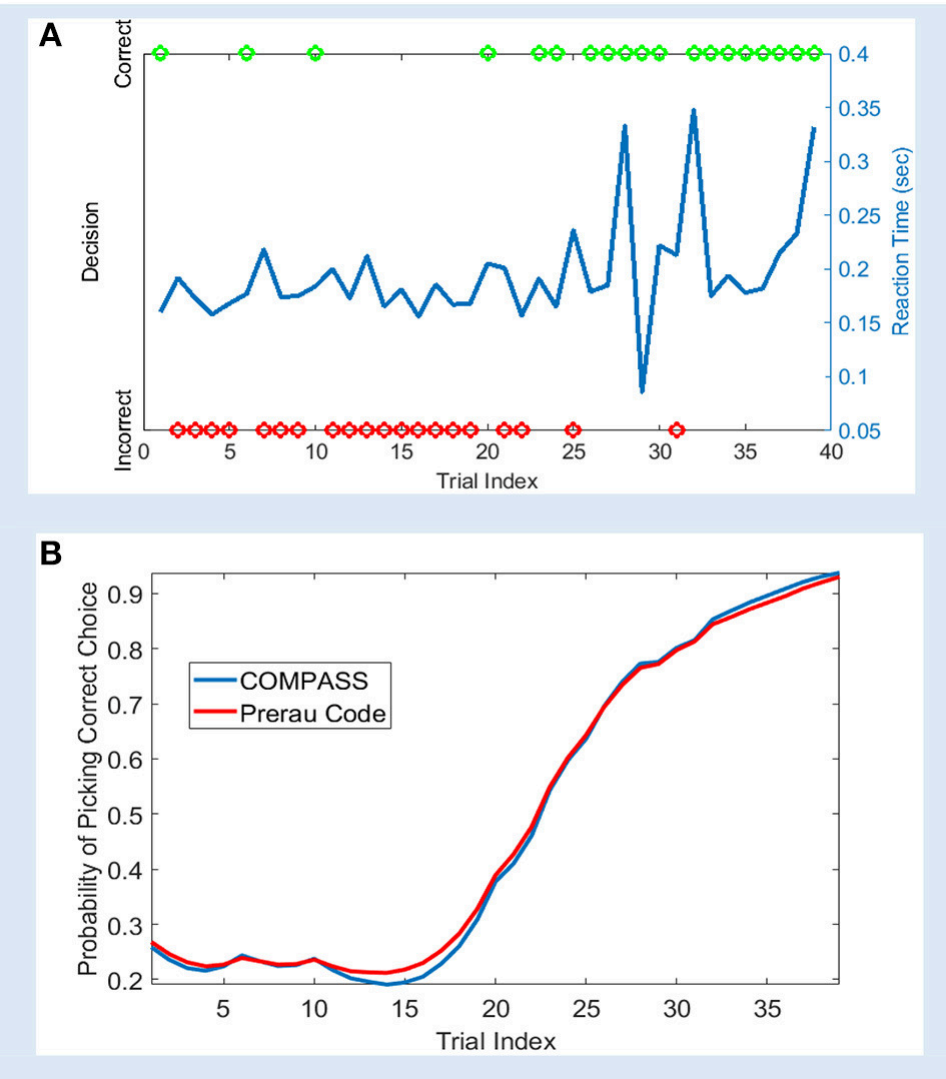
Sample learning behavior and learning state estimation using the method of Prerau et al. (2009) and COMPASS toolbox. **(A)** Correct (green) and incorrect (red) trial outcomes and reaction times (blue) over the course of a single task block. The behavior shows an abrupt change after 20 trials, with the subject beginning to match most stimuli correctly. The animal learns the task with an accuracy above 90% by the end of the trial block; the decision accuracy at the beginning of the block is < 30%. **(B)** The estimated learning curve using the Prerau code (red) and COMPASS toolbox (blue). The slight difference between the toolbox and Prerau et al. result relates to the different stopping criterion used in these methods. Prerau's method sets a preset threshold on the likelihood growth and stops when the likelihood growth falls below the threshold. In COMPASS, *compass_em* stops after a user-specified number of iterations.

We analyzed a sample learning dataset from Williams and Eskandar (2006) with both the original Prerau et al. code and an equivalent model set up in COMPASS (Figure 2). Here, we show an example from one behavioral session, comprising of 61 trials. On each trial, the subject (a rhesus macaque) attempted to learn/perform an arbitrary association between one of four stimulus pictures and four joystick directions. In 39 of the 61 trials, the subject indicated a choice within the required response window. For this example, the 22 non-response trials are excluded from the analysis. Appendix B in the Supplementary Material describes the learning model, and how the model can be replicated using COMPASS. In COMPASS user manual (see Supplementary Material) we show how to impute responses on non-observed trials. Thus, the behavioral signal processed in the toolbox includes the reaction time and decisions over the 39 trials (Figure 2A). The complete model is specified in only 10 lines of code, takes < 20 s to run, and shows almost a complete overlap with the output of the original authors' custom code. We observe an inflection point around trial 15, where the subject's learning state climbs rapidly as associations begin to be performed correctly (Figure 2B). The time to reach this or any other criterion point, or the slope of the learning curve around that point, could be used as a subject-level measurement of learning.

## Example 2: Negative Symptoms and Reward Motivation in Schizophrenia

Another major use of computational modeling is to tease out a differential sensitivity to reward and loss, as in learning, gambling, and approach-avoidance tasks (Bogacz et al., 2006; Anticevic et al., 2015; Veit et al., 2015). In one compelling example, Gold et al. (2012) demonstrated this approach in schizophrenia, showing that patients with prominent negative symptoms were impaired at learning from gains, but not from losses. This was reflected in behavioral modeling, where high-negative-symptom patients specifically showed lower values of a model parameter reflecting how well they could compare gain-seeking to loss-avoiding options. The resulting analysis blended two common reinforcement-learning models, “actor-critic” and “Q-learning,” with the key parameter being how much each model drove or reflected behavior.

Appendix C in the Supplementary Material shows how each term of the Gold et al. hybrid model can be captured in the state-space framework. In brief, the Gold et al. (2012) task involves learning the correct option in four stimulus-action contingencies. A participant is presented with four pairs of landscape items; two pairs involve potential gain and the other two pairs involve loss. For the gain items, the correct response is reinforced by a probabilistic gain—more than 80%—represented by a nickel coupled with the word “Win,” and the phrase “Not a winner” for the incorrect response. For the loss trial, the correct response is reinforced by “Keep your money” with a high probability chance, and “Lose” feedback on the incorrect response. To examine the participants' learning, 160 trials were administered with all pair types presented in a randomized order. Using COMPASS, we represent that learning progression by nine state variables; two state variables per contingency to represent the actor-critic learning process and one global state variable that represents the Q-learning process. The overall progress of learning for a given stimulus is given by a weighted combination of the state variables representing the actor-critic and Q-learning processes.

We analyzed a shared dataset, a subset of the original Gold et al. (2012) dataset which was graciously provided by the authors. On those data, using the Appendix C model in Supplementary Material, COMPASS replicated the original paper's result. The data comprised of 63 study subjects: 26 were healthy controls (HC) and the remaining 37 were clinically stable patients with schizophrenia or schizoaffective disorder. The latter were divided into a high negative symptom (HNS, 19 patients) group and a low negative symptom (LNS, 18 patients) group. Each subject performed 160 task trials, divided into four learning blocks of 40 trials. Behavioral outcomes included response accuracy (correct/incorrect), reaction time, money gained per trial, and trial type (Gain vs. Loss Avoidance).

**Figure 3:**
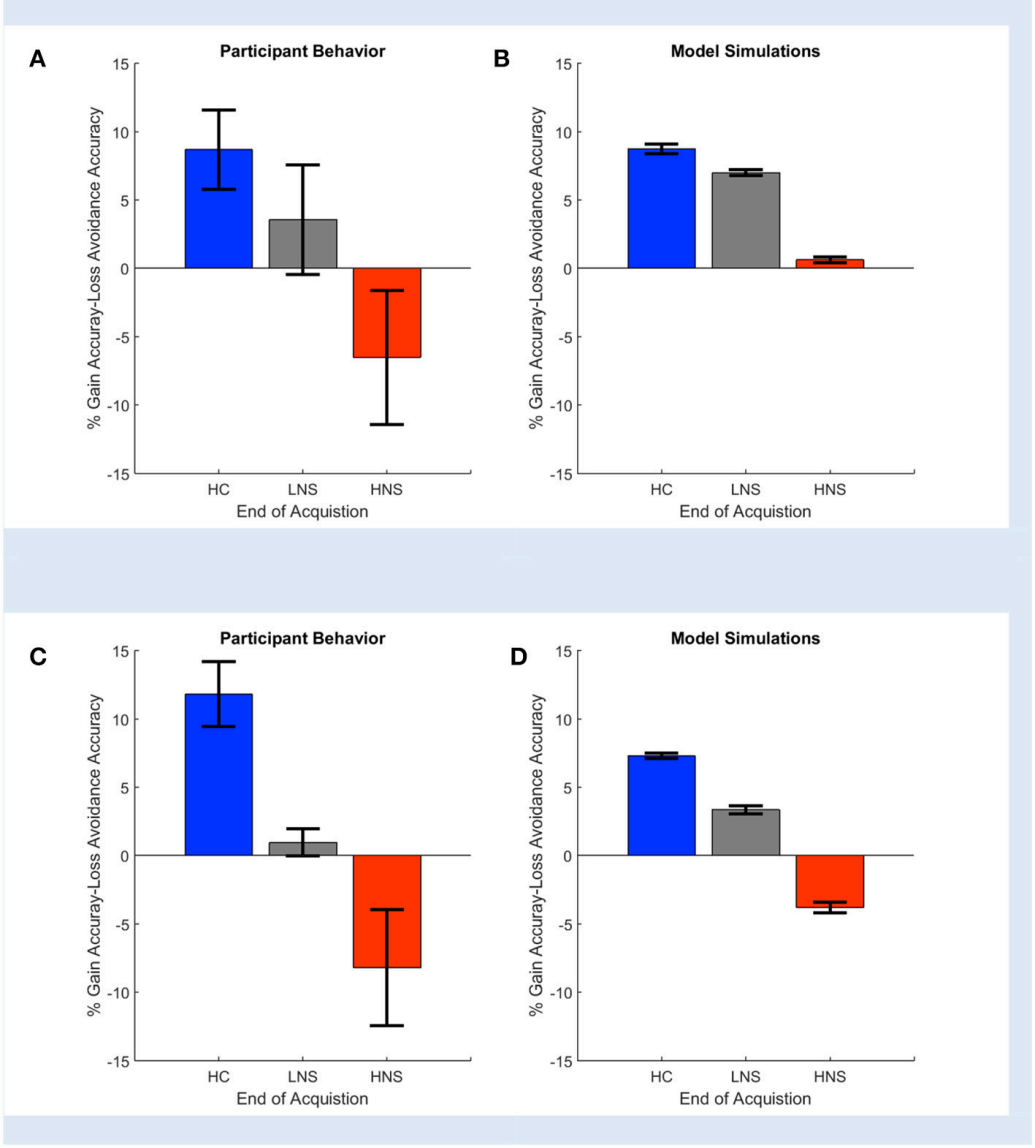
Observed **(A,C)** and simulated **(B,D)** end acquisition performance across patient (LNS, HNS) and healthy control (HC) groups. The HC group prefers learning from Gains (reward) rather than learning from avoided losses. This preference is reduced in the LNS group and inverted in the HNS group. The performance (% Gain accuracy—Loss Avoidance accuracy) is defined as the difference between the probability of picking the correct response on Gain trials and the probability of picking the correct response on the Loss Avoidance trials. **(A)** Observed performance and **(B)** simulated end of acquisition performance originally reported in Figures 4A,B of Gold et al. (2012). Simulated performance is generated from individual patients' estimated model parameters, as described in the original paper. **(C)** Observed performance in the dataset shared by Gold et al. and **(D)** simulated result using an equivalent behavior analysis conducted in COMPASS. For **(D)**, we run the equivalent hybrid model—described in Appendix C in Supplementary Material—per each patient. This gives, for each patient, an estimate (logistic regression coefficient) of the probability of making the correct choice on Gain vs. Loss Avoidance trials. The plot shows the average of these differences for each patient group. The pattern of HC > LNS > HNS on Gain-Loss Avoidance is replicated. Further, COMPASS modeling simulation **(D)** matches the empirical behavior pattern **(C)** more closely than the original authors' simulation.

Gold et al. (2012) reported that HC and LNS subjects learned more from gains than from losses, while HNS subjects' learning was more influenced by loss. This was reflected empirically in a greater accuracy on Gain than on Loss Avoidance trials (Figure 3A). It was also reflected in the modeling and simulation of patients' behavior at the end of the task acquisition. When Gold et al. simulated data based on the model parameters' fit to each individual subject's behavior, HC and LNS subjects were learned more by obtaining gains rather than by avoiding losses (Figure 3B). We replicated this finding using COMPASS. Subjects' behavior on our sample dataset showed the same pattern as in the original paper (Figure 3C). We then fit subject-level models to that behavior and plotted the individual subjects' Gain vs. Loss Avoidance coefficients for accuracy prediction. This replicated the pattern of HC/LNS showing gain sensitivity and HNS showing primarily loss sensitivity (Figure 3D; Appendix C in the Supplementary Material provides a detailed explanation of the Gold et al. computational model using COMPASS). In fact, COMPASS modeling result is slightly more faithful to the empirical behavior pattern than the original Gold et al. simulation. The modeled performance of the HNS group (Figure 3D) is below the X-axis (as it is in the original empirical performance, Figure 3A), whereas the original simulations of Gold et al. produced a mean Gain-Loss difference close to 0 (Figure 3B).

## Example 3: Censored-data Problem

Censored data occurs commonly in trial-structured behavioral experiments and many other forms of longitudinal data, e.g., survival data (Klein and Moeschberger, 2006; Yousefi et al., 2017a). There are multiple reasons that may lead to censored data in behavioral tasks. In many behavioral studies, the experimental design requires that the time window for each trial be limited, in which case the behavioral response is missing, or censored, whenever the response exceeds the time limit. For example, in the continuous-performance test (CPT), which is widely used to assess sustained and selective attention, the inter-trial interval is limited to demand continuous preparation for rapid decision making. The other cause for censored data is insufficient dynamic range or resolution of the measuring apparatus which leads to saturated—or censored—data. Censored data can lead to a severe bias and a reduction of statistical power in a subsequent analysis (Graham, 2009; Yousefi et al., 2017a). COMPASS provides the tools to overcome this issue; using COMPASS, we are not only able to estimate state variable(s) in the presence of censored data points but can also reliably estimate the model parameters. In this example, we simulated a hypothetical experiment where the state variable represents in-attention state. The behavioral readout is reaction time, which is defined as a function of in-attention state. The reaction time is observed when it is below a threshold—e.g., trial time period—and it is censored when the reaction time goes above this threshold. The objective is to estimate in-attention state and model parameters for different threshold levels.

**Figure 4:**
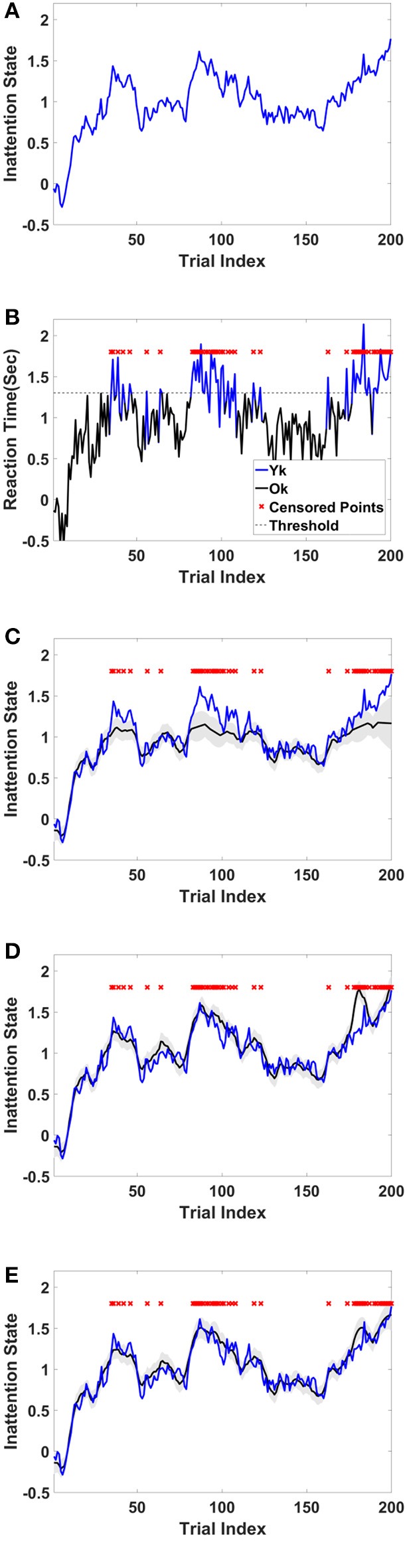
Sample simulated trajectory for inattention state, censored observation, plus state-estimation using naïve, imputation, and full-likelihood technique **(A)** Simulated inattention state. The state starts from a lower value and grows and then drops afterward. This pattern repeats one more time. The first peak is around trail 40, and the second peak is about trial 90. The state grows toward the end of the task, where it reaches its highest value at the last trial, trail 200. **(B)** Observed RT. RT is observed when it is below 1.3 s, and it is censored when it goes above the threshold. Red crosses show those trials where their corresponding RTs are above the threshold. **(C)** State estimation using MAR method. The state estimation fails to follow (true) inattention state trajectory as the censored data points are assumed to be dropped at random. **(D)** State estimation using imputation method. The state estimation reasonably follows (true) inattention state for most of censored data points, however its estimation accuracy drops when the number of consecutive censored points grow (Yousefi et al., 2017a). **(E)** State estimation using full-likelihood method. The state estimation follows (true) inattention state even when the number of censored data points are relatively high. For the full-likelihood method RMSE has the lowest value (0.0952), and MAR method RMSE is significantly higher than two other methods (0.1739). RMSE for imputation method is 0.1223. For this threshold, 49 of 200 trial are censored. This result suggests that MAR method in state estimation will lead to a large bias in the state estimation, and it is a less proper choice when number of censored points are relatively high. Here, we assume variance of the state and observation processes noise are known.

**Figure 5:**
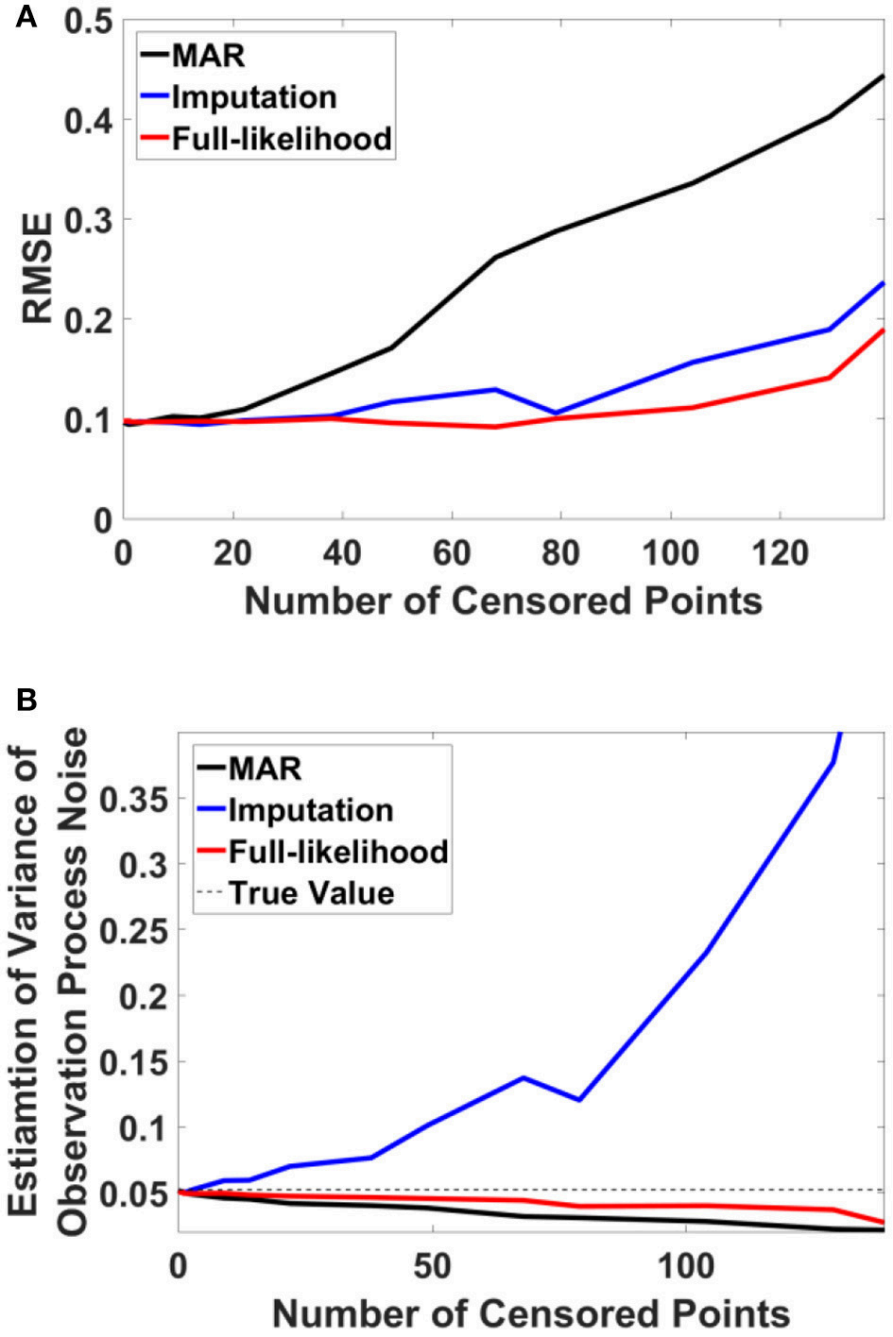
RMSE and observation noise parameter estimation in the censored data problem. **(A)** RMSE via censored data points. RMSE for naïve, imputation, and full likelihood method as a function of censored data points—or, different threshold level. The full likelihood method attains the lowest RMSE independent of the number of censored data points. Naïve method has the highest RMSE, which replicates its less accurate estimation of the state. **(B)** Observation noise parameter estimation. Full likelihood shows a more robust and consistent estimation of the observation noise variance. Naïve method shows a fairly robust estimation of the observation process noise variance, but its estimation is lower than the full likelihood method. Generally, the naïve method tends to under-estimates the state noise variance; the state variable on censored points are generally on the tail of their distribution and naïve method ignores those points in its estimation of the state process noise. Estimation of the observation process noise variance turns to be sensitive using the imputation method. Imputation method tries to replace those censored data points with a sampled one, and these samples which are generated from the tail of distribution induces more noise to the observation process.

For this example, we simulated the data to generate in-attention state and reaction time for 200 trials. We then used different thresholds to generate censored data points (Figures 4A,B show the inattention state, reaction time, and censored data points for a specific threshold), and utilized COMPASS to estimate the model parameters and state variables given for the different numbers of censored data points. COMPASS has the capability of running naïve—e.g., MAR assumptions, imputations, and full-likelihood techniques on censored data points, to estimate both the state variable and the model parameters. Figures 4C–E show the state estimation result for all three methods. We found that the likelihood method estimates the state variable even when a sequence of data points was being censored. We also utilized COMPASS to estimate the model state and observation process noise variance. Figures 5A,B show the estimation result using the three techniques for different threshold levels. Again, the likelihood method maintains an accurate estimation of the model parameters even when 50% of data points are being censored. The simulation result suggests that we may extract proper information from the behavioral data even if a significant percentage of the readout is being censored—or dropped—given the task specification or data recoding issue. Appendix D in the Supplementary Material describes the in-attention state and observation process models and how the data is generated in MATLAB. We also show COMPASS functions can be called for the state and model parameters estimation. A further discussion of the model implementation and its performance results can be found in COMPASS manual.

## Discussion

We developed an open-source toolbox for state-space modeling and demonstrated its utility in analyzing dynamical behavioral signals relevant to computational psychiatry. In two examples from relevant literature, we showed that COMPASS replicates the results of prior modeling studies. In one of those examples, COMPASS estimated model parameters that more faithfully reproduced the empirical results. The fact that COMPASS has the capability to incorporate further characteristics of the observed behavioral data—like missing or censored data—in its analysis allows us to run a more versatile inference analysis and derive an analytical result with a higher statistical significance. In the third example, we demonstrated the capability of COMPASS in the state and parameter estimation with censored data points. In the toolbox manual, we also re-analyzed the same behavioral data presented in Example 1, considering the missing and censored data points. COMPASS capability of analyzing missing—or censored—data has multifold benefits; this not only gives a better sense about the data, but also addresses other ambiguities like non-uniform interval sampling being imposed by dropping missing and censored data. The same statement is valid in the second example; though most parameters in Gold et al. (2012) were pre-set, we estimated almost all the model parameters in COMPASS. Using COMPASS goodness-of-fit function, we were capable of studying the significance of the model parameters plus the underlying learning mechanisms utilized in the Gold et al. model. The utility of COMPASS goes beyond replicating those models; we can build different models for different hypothesis in a minimum time and utilize COMPASS goodness-of-fit and estimation result to reject or validate those hypotheses.

The state-space modeling framework is a core tool in many research fields, especially engineering (Ogata and Yang, 1970; Aoki, 2013). It is paving the way into neuroscience, psychiatry, and other medical research (Barbieri et al., 2004; Chen et al., 2010; Paninski et al., 2010). COMPASS takes steps toward providing unified and high-level functions that make a range of state-space models straightforward to implement and use for a wide variety of behavioral signals. Users can build a wide range of models to analyze behavioral data and compare their results in a principled way. A further explanation and guideline for building and comparing model forms can be found in the toolbox manual. The functions *compass_deviance* and *compass_param_covariance_info* enables model comparison. Further, COMPASS includes “on-line mode” functions that can continuously update state variables in real time as new data are acquired. The toolbox function *compass_filtering* addresses this, and examples of its use are given in the toolbox manual. In on-line operation, one can also call the learning algorithm periodically to re-update model parameters. Thus, COMPASS can also be used to construct experiments with adaptive behavior paradigms, where the stimuli presented to each subject are adjusted based on its past performance. This approach could more efficiently sample regions of behavioral interest, e.g., the equipoise boundary of a choice task as modeled in Amemori et al. (2015) and Karmali et al. (2016). It could also drive real-time application of a study intervention, e.g., brain stimulation or optogenetic (in)activation (Grosenick et al., 2015; Widge et al., 2017).

The state-space modeling framework is not limited to normally distributed signals or discrete binary observations. COMPASS includes methods for highly skewed observation distributions (gamma and log-normal distributions) and for optimally imputing missing/censored data (Shih, 2002; Mathys et al., 2011). The distribution assumption is defined by arguments to the *compass_em* function, as are methods for censored data. These additions make COMPASS a powerful and versatile package for analysis of many different classes of dynamical signals. The main limitation of the state-space modeling framework is that prior to now, development and debugging of these models has been difficult. Development requires tedious work and extensive time and involves statistical and programming skills that are not yet common in the field of cognitive neuroscience. We hope that by providing this toolbox, we can help other researchers delve into computational behavior analysis with a much lower barrier to entry.

COMPASS' utilities to deal with non-Gaussian and incomplete data are one of the toolbox's strengths. Another strength is its ease of use, and its flexibility to build and compare different behavioral models—in the toolbox manual, we provide an example and COMPASS function which enables us to examine different modeling assumptions on the same dataset. Another key feature of COMPASS is its capability of analysis of mixed signals with Normal/Bernoulli, Gamma/Bernoulli, and point process data which make it a suitable platform for analysis of a wide variety of neural and behavioral data. However, there are other functions that can be added to COMPASS to make its applications broader. Though COMPASS addresses Bernoulli, Normal, Gamma, and Point-Process observation processes, there are other modalities of neural and behavioral data which might be better described by distributions like Beta or multinomial. For example, in associative learning, when a participant learns multiple stimuli, we can use a multinomial observation process to characterize learning with respect to multiple categories of the task (Yousefi et al., 2017b). We are currently working to add state-space models capable of processing these types of observations to COMPASS. COMPASS addresses one category of censored data—censored from above; however, addressing other forms of censored data—censored from below or censored from above-below—might have applications in behavioral and neural data like fMRI. COMPASS's addition is a point-process observation process and a particular class of marked-point process, where the mark follows a Bernoulli process. Extending the domain of marking to multivariate observations has received a lot of recent interest and its addition to COMPASS might attract more researchers from the neuroscience and BMI domains (Deng et al., 2015; Truccolo, 2016). We are in the process of building wrapper functions which make model comparison easier for individuals with a basic familiarity with MATLAB programming and scripting, plus an extension of the observation process dimension which will help researchers in analyzing behavioral and neural data captured from even more complex tasks. We are consistently working on the toolbox and add new functions as we progress and develop new theories and algorithms for analysis of neural and behavioral data. Indeed, the point of the foundational features of COMPASS allow for easy expansion and optimization for use in a wide multivariate behavioral domain, allowing researchers in a wide array of fields to apply these approaches to their data.

## Disclosure

AY, AP, AW, EE, DD, and UE reported their patent application on “System and methods for monitoring and improving cognitive flexibility”—WO2017004362A1. DD and AW reported research support and consulting/honoraria from Medtronic. DD reported research support from Eli Lilly, Roche, and Cyberonics. AW reported consulting income from Livanova and Circuit Therapeutics. AW reported research support from the Brain & Behavior Research Foundation, OneMind Institute, and Picower Family Foundation. AY, IB, AP, AW, EE, DD, and UE are partially supported by the Defense Advanced Research Projects Agency (DARPA) under Cooperative Agreement Number W911NF-14-2-0045 issued by ARO contracting office in support of DARPA's SUBNETS Program.

## Author Contributions

All authors listed have made a substantial, direct and intellectual contribution to the work, and approved it for publication.

### Conflict of Interest Statement

The authors declare that the research was conducted in the absence of any commercial or financial relationships that could be construed as a potential conflict of interest.
