# Supplementary material for: COMPASS: An Open-Source, General-Purpose Software Toolkit for Computational Psychiatry
Source: Front Neurosci. 2019 Jan 11;12:957. doi: 10.3389/fnins.2018.00957 (PMC6336923; doi:10.3389/fnins.2018.00957)
Supplement: Supplementary file 1 [file Data_Sheet_1.docx]

**Appendix A** Model Inference in COMPASS

Here, we discuss different aspects of the COMPASS toolbox and particularly its parameter estimation properties. We use EM and MLE estimation in model inference and will discuss their relationship with another common inference method: Variational Bayes. We then discuss how COMPASS can be used in single and patient group studies. Finally, we discuss parameter initialization which can be utilized in COMPASS to have a better model estimation.

1. Expectation Maximization via Variational Bayes Inference Method

COMPASS utilizes MLE and EM algorithms to estimate the state-space model free parameters. The estimation result consists of a local maximum of the model parameters plus the posterior distribution of the cognitive state. The other widely used technique in the inference step is variational Bayes (66, 67, 68), where the maximum local estimate of the model parameter is substituted by consecutive updates of the model parameters’ posterior distribution. In a previous research, we showed an application of variational Bayes in estimating parameters of a state-space model built for a multiple-choice decision-making task (68). In this research, we observed that the variance of parameters’ posteriors shrinks over multiple iterations. This suggests that EM and the resulting point estimate might provide a good estimate of the model parameters when it is equipped with a good parameter initialization. COMPASS also provides a function to calculate the covariance estimate of the model estimated parameters - *compass_param_covariance_info*. This function can be used to build the posterior distribution for the model parameters – this corresponds to the asymptotic normality of MLE.

COMPASS models are built based on our prior understanding of the observed signals and their link to cognitive state. This information is helpful to properly initialize the model parameters – we will discuss this in the following paragraphs. We can also initialize the model parameters with different values and assess their likelihood growth when there is relatively more or less knowledge about the model parameters. *compass_em* and *compass_deviance* functions provide information needed for this path of model analysis.

1. Single Subject via Group Studies in COMPASS

There is a significant body of research that seeks to characterize inter and intra behavioral or neural signals under different tasks (69, 70). COMPASS is a toolbox for analysis of dynamical signals, and thus the sequence of the task trials or observed signals plays a significant role in the modeling. This property is better aligned with single subject studies; however, COMPASS' flexibility in dealing with multi-dimensional state variables, multi-dimensional input and missing data can be utilized to build models applicable in group studies.

The first and naïve approach to extend a single subject study to a group study will be building one model per subject and studying statistical properties of the inferred parameter and state variables collectively. This is effectively the approach taken in our Gold et al. example. However, there are many other ways that we can leverage COMPASS' flexibility to build models for group study purposes. For instance, we can concatenate different subjects observed behavioral data and add a large sequence of missing data between subjects’ data. This reduces the dependence between subjects’ state variables but permits the building of a state space model for the group. Under this modeling assumption, we estimate one set of parameters for the group along with a patient specific cognitive state estimation. Another possible solution is using indicator functions per subject and embedding them into the model input – $(U_{k},I_{k})$. Under this modeling assumption, we can define common and subject specific parameters or state variables and use COMPASS to inference the model parameters. Please note these are only two possible solutions and we can adopt different forms of models for group study purposes. Note further that *compass_param_covariance_info* and *compass_deviance* can be used to assess the extent of fit or to examine different statistical test given the model parameter estimate.

1. Model Parameter Initialization

COMPASS has a default initialization for its model parameters, which might not be optimal depending on the parameter definition. Imperfect initialization might also increase the number of training iterations – and as a result, the computation time – in COMPASS. However, COMPASS lets users manually set these parameters. Let’s assume the state variables are fixed – with a zero variance – in the observation process. The resultant models – with a mild assumption – become GLM models (71). Thus, we can utilize a static GLM fit (MATLAB, Generalized linear model regression, glmfit) to the observed data to initialize the observation model's free parameters. For the state transition process, we generally start with a multi-dimensional random walk model. The state variable dimension is defined by the model specification; under this assumption, the model free parameters are *Param.Wk.* We normally start with a diagonal matrix where we can change the level of noise – diagonal elements – and study model fit under different starting points.

**Appendix B** Formulating Multivariate Associative Learning Model

Prerau et al’s model (29) utilizes a random-walk model to characterize “learning state” over the task trials. $x(k)$ is the learning state at trial $k$ and it is defined by

$x\left( k+1 \right)=x\left( k \right)+\varepsilon_{k} \varepsilon_{k}\sim N(0,\sigma_{\varepsilon}^{2})$ (A.1)

where, $\varepsilon_{k}$ is an i.i.d. normal noise characterizing possible changes in the learning state per each trial. The noise variance – $\sigma_{\varepsilon}^{2}$ – is unknown, and its value is estimated as a part of the model fitting.

The response time – $y_{c}(k)$ – is modeled by a log-normal distribution, where the distribution mean is defined by a linear function of the learning state

$\log y_{c}\left( k \right)\sim N(c_{2} x\left( k \right)+c_{1},\sigma_{c}^{2})$ (A.2)

where, $c_{1}$ and $c_{2}$ define the relationship between the reaction time and learning state. The variability in the reaction time (in logarithmic scale) is characterized by an i.i.d. white noise with variance $\sigma_{c}^{2}$. $exp(c_{1})$ is the expected reaction time, and it is scaled by $exp(c_{2} x(k))$ as the learning state evolves over the course of experiment.

The probability of a correct response – $P_{k}$ – on $k^{th}$ trial is defined by

$\log\left( \frac{P_{k}}{1-P_{k}} \right)= x\left( k \right)+d_{1}$ (A.3)

where, $d_{1}$ is a free parameter and it defines the probability of choosing a correct response in absence of the learning state.

Let’s assume a participant learns the task; this corresponds to picking more frequent correct responses over consecutive trials. Under this assumption and given the model definition, $x\left( k \right)$ grows as the task proceeds. Now, we can study how the response time progresses over the experiment. A positive $c_{1}$ suggests that the participant requires a longer time in choosing the correct choice – or, the response time increases as the learning state grows; whereas, a negative $c_{1}$ is associated with a faster response as the learning state grows – or, the participant response time decreases as the task is being learnt.

We can build this model using COMPASS functions. Figure A.1 shows the model implementation in MATLAB. We can then use COMPASS functions to estimate the model free parameters – $(c_{1},c_{2},\sigma_{\varepsilon}^{2},\sigma_{c}^{2},d_{1})$ – along with posterior estimation $x\left( k \right)$.

%% Load behavioral data and prepare it for the toolbox

% Data: Yb , Yn

load('example_a.mat');

% Yn - logarithm of reaction time

ind = find(~isnan(Yn));

Yo = Yn(ind)/1000;

Yn = log(Yn(ind)/1000);

% Yb - decision (0/1)

Yb = Yb(ind);

N = length(Yn);

% Input - 1 xi

In = zeros(N,2);

In(:,1)= 1;

In(:,2)= 1;

% Input, Ib is equal to In

Ib = In;

% Uk, which is all zero

Uk = zeros(N,1);

% valid, which is valid for the observed data point

Valid = ones(N,1);

%% Build behavioral model and learning procedure

% create model

Param = compass_create_state_space(1,1,2,2,eye(1,1),1,1,1,0);

% set learning parameters

Iter = 250;

Param = compass_set_learning_param(Param,Iter,0,1,0,1,1,1,1,1,0);

%% Run learning with a mixture of normal & binary

[XSmt,SSmt,Param,XPos,SPos,ML,YP,YB]=compass_em([1 1],Uk,In,Ib,Yn,Yb,Param,Valid);

%% Deviance analysis

[DEV_C,DEV_D]= compass_deviance([1 1],In,Ib,Yn,Yb,Param,Valid,XSmt,SSmt);

**Figure A.1** Analysis script for the associative learning task

In the model implementation shown in Figure A.1, we drop all trials with an unobserved or censored response. We can consider incorporating those categories of the observed signal in the model, which can be addressed by changing the *Valid* argument passed to *compass_em* and using *compass_set_censor_threshold_proc_mode.*

Prerau et al. used a random-walk model to characterize the learning state evolution over the course of the experiment; however, COMPASS has the utility to change the state evolution model to an autoregressive model of order $p$ – $AR(p)$. For example, we can build the learning state using an $AR(1)$ model which is described by

$x\left( k+1 \right)=a x\left( k \right)+b u\left( k \right)+\varepsilon_{k} \varepsilon_{k}\sim N(0,\sigma_{\varepsilon}^{2})$ (A.4)

where, $(a,b)$ are the model free parameters describing the state evolution. $u\left( k \right)$ is the input to the model, which might bring other factors of the task into the model including reward or history terms. For instance, let’s assume we observed that the returned reward per correct decision choice might boost the learning state and we want to incorporate this observation in the model. The reward is given randomly, but the probability of reward on correct choices is much higher than the incorrect choices. In COMPASS, $u(k)$ reflects the reward being injected into the model. We assume the chance of receiving a reward is 90% on the correct trials and is 20% on incorrect trials. Figure A.2 shows an example implementation of this new model – here, we build the reward based on our assumption and incorporate it in the model.

The model presented in Prerau et al. assumes that response time – $y_{c}(k)$ – and decision choice – $y_{b}(k)$ – are independent variables given the learning state – $x(k)$. Generally, we are interested in building the joint probability distribution of $y_{c}(k)$ and $y_{b}\left( k \right).$ Using COMPASS functions, $y_{c}(k)$'s probability distribution can be defined as a function of $y_{b}(k)$ and other covariate terms. For instance, we can build the following model for response time

%% Load behavioral data and prepare it for the toolbox

% Data: Yb , Yn

load('example_a.mat');

% Yn - logarithm of reaction time

ind = find(~isnan(Yn));

Yo = Yn(ind)/1000;

Yn = log(Yn(ind)/1000);

% Yb - decision (0/1)

Yb = Yb(ind);

N = length(Yn);

% Input - 1 xi

In = zeros(N,2);

In(:,1)= 1;

In(:,2)= 1;

% Input, Ib is equal to In

Ib = In;

% Uk, which is returned reward

Uk = zeros(N,1);

ind = find(Yb==1);

temp = rand(length(ind),1)>0.1;

Uk(ind)= temp;

ind = find(Yb==0);

temp = rand(length(ind),1)>0.8;

Uk(ind)=temp;

% valid, which is valid for the observed data point

Valid = ones(N,1);

%% Build behavioral model and learning procedure

% create model

Param = compass_create_state_space(1,1,2,2,eye(1,1),1,1,1,0);

% set learning parameters

Iter = 250;

Param = compass_set_learning_param(Param,Iter,1,1,1,1,1,1,1,1,0);

%% Run learning with a mixture of normal & binary

[XSmt,SSmt,Param,XPos,SPos,ML,YP,YB]=compass_em([1 1],Uk,In,Ib,Yn,Yb,Param,Valid);

%% Deviance analysis

[DEV_C,DEV_D]= compass_deviance([1 1],In,Ib,Yn,Yb,Param,Valid,XSmt,SSmt);

**Figure A.2** Analysis script for the associative learning task with a reward term

$\log y_{c}\left( k \right)\sim N(c_{3} y_{b}(k)+ c_{2} x\left( k \right)+c_{1},\sigma_{c}^{2})$ (A.5)

Under this modeling assumption, $y_{c}(k)$ and $y_{b}\left( k \right)$ are not independent variables. Thus, we can build different joint probability distribution functions exploring possible relationships between state variable and behavioral signals.

**Appendix C:** Reformulating the Hybrid Learning Model Using the State-Space Modeling Framework

Gold et al's. goal was to provide a quantitative fit to the pattern of data observed in patients and healthy controls. They investigated both a standard Actor-Critic architecture and a Q-learning architecture. They argued that neither taken alone could account qualitatively for both healthy control and patient data. They thus investigated a mixture model of Actor-Critic and Q-learning, which led to better qualitative and quantitative fits for all groups and explained key features of the data. The actor-critic model uses reward prediction errors to modify the probability of selecting an action, while the Q-learning model predicts sensitivity to actual outcome values. That is, Q-learning is more focused on maximizing total value, and therefore predicts that subjects will choose a high chance of gain over a high chance of avoiding a loss. Actor-critic would value those two choices equally, because they have similar chances for prediction error. Given that different patient groups showed a mixture of these strategies in responding to the task, the hybrid model should be able to better account for observed results in patients and healthy subjects.

Here, we show how the model of Gold et al. (5) can be implemented using the state-space modeling framework and thus the COMPASS toolbox. **Table A.1** shows a line-by-line comparison between two models. Here, we focus on the Hybrid model proposed in the paper. The pure actor-critic or Q-learning processes are special cases of this model with parameter *c* (see below) set to 0 or 1, respectively. Please note that we selected this complex reinforcement learning model to demonstrate COMPASS' flexibility in replicating complex dynamical models. There are many simpler reinforcement learning models which can be used in characterizing observed behavioral signal(s). Indeed, we describe one such simpler model in the prior Appendix. Given COMPASS' capacity in replicating this complex hybrid reinforcement learning model, its adaptation to simpler reinforcement models is effortless.

The model is cast in terms of the trial type – $s$ – and the possible actions within that trial type – $a$. *a* can be seen as picking one of two actions, where a correct action on the gain trials corresponds to picking the item associated with winning the reward. The correct action on the loss-avoidance trials corresponds to picking the item that is not associated with a penalty. Note that we only need to represent one of these two actions per category with a state variable; this is because taking an action implies that the other action is not being taken. We assign two state variables to each task’s category to represent the value of being in a state and of taking action 1 during that state. That is, we need $x_{s_{1}}(k)$ and $x_{a_{1}s_{1}}\left( k \right)$ to present the probability of the stimulus being category 1 and the probability of taking the action *a* when in category 1. We assign one state variable to represent the global value of taking action 1 across all possible states/trial types – $x_{a_{1}}(k)$. Now, we can define the state-transition process for the task by (note that action $a_{1}$ will be common across states):

***Table A.1*** *Hybrid model of actor-critic and Q-learning and its equivalent state-space model*

| **Hybrid Model** | **Mapping** | **State-Space Model** |
| --- | --- | --- |
| Value for each category – $s\in\left\{ s_{1},s_{2},s_{3},s_{4} \right\}$ – is updated by – $k$ is the trial index  $\ddot{a}\left( k \right)=r\left( k \right)-V\left( s,k \right)$  $V\left( s,k+1 \right)=V\left( s,k \right)+w_{v,s}\ddot{a}\left( k \right)$ | $x_{s}\left( k \right)=V\left( s,k \right)$ | Latent state variables to represent value dynamics per each category – $s\in\left\{ s_{1},s_{2},s_{3},s_{4} \right\}$  $x_{s}\left( k+1 \right)=\left( 1-w_{v,s} \right)x_{s}\left( k \right)+w_{v,s}r\left( k \right)+\varepsilon_{s}$  $\varepsilon_{s}\sim N\left( 0,\sigma_{s}^{2} \right) \sigma_{s}^{2}\to0$ |
| Critics’ weights are updated by actions – there are two actions in this task (left or right image) – $a\in\{a_{1},a_{2}\}$.  $w(s,a,k+1)= w(s,a,k) + w_{c,sa}ä(k)$ | $x_{as}(k)=w\left( s,a,k \right)$ | Latent variables to represent critics’ weights for taking action *a* when in states*.* There are thus in total $2\times s$ latent variables  $x_{as}\left( k+1 \right)=x_{as}\left( k \right)-w_{c,sa}x_{s}\left( k \right)+w_{c,sa}r\left( k \right)+\varepsilon_{as}$  $\varepsilon_{as}\sim N\left( 0,\sigma_{as}^{2} \right) \sigma_{as}^{2}\to0$ |
| Q-learning – learning the action directly  $Q(a,k+1)=Q(a,k)+w_{q,a}(r(k) -Q(a,k))$ | $x_{a}(k)=Q\left( a,k \right)$ | Latent variables to represent Q-learning – the latent variable represents the probability of taking action 1, and its complement the probability of action 2.  $x_{a}\left( k+1 \right)=\left( 1-w_{q,a} \right)x_{a}\left( k \right)+w_{q,a}r\left( k \right)+\varepsilon_{a}$  $\varepsilon_{a}\sim N\left( 0,\sigma_{a}^{2} \right) \sigma_{a}^{2}\to0$ |
| Action probability in the hybrid model  $H(s,a,k)=[(1-c)w(s,a,k)+c Q(a,k)]$  $P(a,k)={exp(H(s,a,k))}/{\sum_{a} \exp\left( H(s,a,k) \right)}$ | $z_{k}$: action at time $k$  action 1: $z_{k}=1$  action 2: $z_{k}=0$ | Model of observation process  $z_{k}$: observation at time $k$, either action 1 or action 2.  $\mathrm{logit}p\left( z_{k}=1 \right)= (1-c)x_{as}\left( k \right)+cx_{a}(k)$ |
| Hybrid Model Using the State-Space Modeling Framework. For clarity, we show the case where there is a single trial type being learned, i.e. we represent only variables $x_{s_{1}}$ , $x_{a_{1}}$, and $x_{a_{1}s_{1}}$. For four trial types as in the Gold et al. study, we would add six more state variables representing trial types 2 through 4 and the corresponding action values in those trial types. | | |
| State Variable Definition  $X_{k}=\left[ x_{s_{1}}(k) x_{a_{1}s_{1}}(k) x_{a_{1}}(k) \right]^{'}$  $U_{k}=r\left( k \right)$  $W\sim N\left( 0,\left[ \begin{matrix} \sigma_{s_{1}}^{2} & 0 & 0 \\ 0 & \sigma_{a_{1}s_{1}}^{2} & 0 \\ 0 & 0 & \sigma_{a_{1}}^{2} \end{matrix} \right] \right)$  $X_{k+1}=\left[ \begin{matrix} 1-w_{v,s_{1}} & 0 & 0 \\ 1 & -w_{c,s_{1}a_{1}} & 0 \\ 0 & 0 & 1-w_{q,a_{1}} \end{matrix} \right]X_{k}+\left[ \begin{matrix} w_{v,s_{1}} \\ w_{c,s_{1}a_{1}} \\ w_{q,a_{1}} \end{matrix} \right]U_{k}+W$  Observation Model  $\mathrm{logit}p\left( z_{k}=1 \right)=\left[ \begin{matrix} 0 & (1-c) & c \end{matrix} \right] X_{k}$ | | |

$X_{k}=\left[ {x_{s}}_{1}\left( k \right) x_{a_{1}s_{1}}\left( k \right) {x_{s}}_{2}\left( k \right) x_{a_{1}s_{2}}\left( k \right) {x_{s}}_{3}\left( k \right) x_{a_{1}s_{3}}\left( k \right) {x_{s}}_{4}(k) x_{a_{1}s_{4}}(k) x_{a_{1}}(k) \right]^{'}$ (A.6.a)

$U_{k}=\left[ r\left( k \right)*I_{s_{1}}\left( k \right) r\left( k \right)*I_{s_{2}}\left( k \right) r\left( k \right)*I_{s_{3}}\left( k \right) r\left( k \right)*I_{s_{4}}\left( k \right) \right]^{'}$ (A.6.b)

$$X_{k+1}=\overset{\mathbf{Ak}}{\overbrace{\left[ \begin{matrix} \begin{matrix} 1-w_{v,s_{1}} & 0 & 0 \\ 1 & -w_{c,s_{1}a_{1}} & 0 \\ 0 & 0 & 1-w_{v,s_{2}} \end{matrix} & \begin{matrix} 0 & 0 & 0 \\ 0 & 0 & 0 \\ 0 & 0 & 0 \end{matrix} & \begin{matrix} 0 & 0 & 0 \\ 0 & 0 & 0 \\ 0 & 0 & 0 \end{matrix} \\ \begin{matrix} 1 & 0 & 0 \\ 0 & 1 & 0 \\ 0 & 0 & 1 \end{matrix} & \begin{matrix} -w_{c,s_{2}a_{1}} & 0 & 0 \\ 0 & 1-w_{v,s_{3}} & 0 \\ 0 & 1 & -w_{c,s_{3}a_{1}} \end{matrix} & \begin{matrix} 0 & 0 & 0 \\ 0 & 0 & 0 \\ 0 & 0 & 0 \end{matrix} \\ \begin{matrix} 0 & 0 & 0 \\ 0 & 0 & 0 \\ 0 & 0 & 0 \end{matrix} & \begin{matrix} 0 & 0 & 0 \\ 0 & 0 & 0 \\ 0 & 0 & 0 \end{matrix} & \begin{matrix} 1-w_{v,s_{4}} & 0 & 0 \\ 1 & -w_{c,s_{4}a_{1}} & 0 \\ 0 & 0 & 1-w_{q,a_{1}} \end{matrix} \end{matrix} \right]}}X_{k}+\cdots$$

${\overset{\mathbf{Bk}}{\overbrace{\left[ \begin{matrix} \begin{matrix} w_{v,s_{1}} \\ w_{c,s_{1}a_{1}} \\ 0 \end{matrix} \\ \begin{matrix} 0 \\ 0 \\ 0 \end{matrix} \\ \begin{matrix} 0 \\ 0 \\ w_{q,a_{1}} \end{matrix} \end{matrix} \begin{matrix} \begin{matrix} 0 \\ 0 \\ w_{v,s_{2}} \end{matrix} \\ \begin{matrix} w_{c,s_{2}a_{1}} \\ 0 \\ 0 \end{matrix} \\ \begin{matrix} 0 \\ 0 \\ w_{q,a_{1}} \end{matrix} \end{matrix} \begin{matrix} \begin{matrix} 0 \\ 0 \\ 0 \end{matrix} \\ \begin{matrix} 0 \\ w_{c,s_{3}} \\ w_{c,s_{3}a_{1}} \end{matrix} \\ \begin{matrix} 0 \\ 0 \\ w_{q,a_{1}} \end{matrix} \end{matrix} \begin{matrix} \begin{matrix} 0 \\ 0 \\ 0 \end{matrix} \\ \begin{matrix} 0 \\ 0 \\ 0 \end{matrix} \\ \begin{matrix} w_{v,s_{4}} \\ w_{c,s_{4}a_{1}} \\ w_{q,a_{1}} \end{matrix} \end{matrix} \right]}}U}_{k}+W_{k}$ (A.6.c)

$\mathrm{logit}p\left( z_{k}=1 \right)=\overset{\mathbf{E}}{\overbrace{\left[ \begin{matrix} 0 & \left( 1-c \right)I_{s_{1}}(k) & \begin{matrix} 0 & \begin{matrix} \left( 1-c \right)I_{s_{2}}(k) & \begin{matrix} 0 & \begin{matrix} \left( 1-c \right)I_{s_{3}}(k) & \begin{matrix} 0 & \begin{matrix} \left( 1-c \right)I_{s_{4}}(k) & c \end{matrix} \end{matrix} \end{matrix} \end{matrix} \end{matrix} \end{matrix} \end{matrix} \right]}} X_{k}$ (A.6.d)

where, $I_{s_{i}}(k)$ is an indicator function which is 1 when the current trial type corresponds to stimulus category $i.$ In equation (A.6.c), the probability of taking each action is defined as a function of the learning, captured in the vector $X_{k}$. $X_{k}$ represents both actor-critic and Q-learning values – the first 8 entries describe actor-critic learning for each trial type, while the 9^th^ element represents the global Q-learning process that focuses solely on the action regardless of the trial type. As Gold et al. did, we represent the relative weight of these two learning processes (actor-critic and Q-learning) through a mixing parameter *c.* Using the Logit function defined in (A.6.d), the probability of taking action $a_{1}$ is defined by $cx_{a_{1}}\left( k \right)$ in the absence of the actor-critic model. The probability of taking action $a_{1}$ when presented with trial type $s_{1}$, in the actor-critic model, is defined by $\left( 1-c \right)x_{a_{1}s_{1}}\left( k \right)$. The overall probability of taking action $a_{1}$ when presented with $s_{1}$ is thus defined by $cx_{a_{1}}\left( k \right)+\left( 1-c \right)x_{a_{1}s_{1}}\left( k \right)$. We can similarly define the probability of both actions for the other 3 trial types. Given the model definition, an increase in $x_{a_{1}}(k)$ implies a global preference for $a_{1}$ in all trial types, while an increase in $x_{a_{1}s_{1}}\left( k \right)$ implies a specific preference for $a_{1}$ when presented with $s_{1}$.

The model of Table A.1 translates directly into MATLAB code, as shown in Figure A.3. This close mapping between model specification and computer code makes COMPASS easier to use and interpret.

In the script, RATE_A and RATE_B define initial values for $w_{v,s_{i}}$ and $w_{c,s_{i}a_{1}} i=1\cdots4$. RATE_C defines an initial value for $w_{q,a_{1}}$. The initial values for RATE_A, RATE_B, and RATE_C are set to 0.1. This setting defines initial values for elements of *A* and *B* matrices, which defines how new reward information is combined with the previous estimation of state variables. A value close to 0 indicates that the state variable is derived solely from current reward, meaning no state dependency in the behavior, and values close to 1 indicate reward accumulation over consecutive trials in deriving behavior. Setting RATE_A, RATE_B, and RATE_C to 0.1 means that the reward in the current trial contributes to 10% of state variable update whilst 90% of the state variable is carried forward from its previous value. The optimal values for $w_{v,s_{i}}$, $w_{c,s_{i}a_{1}}$, and $w_{q,a_{1}}$ are smaller than 1 and preferably close to zero, if the values for the critics’ weights and values dynamics (**Table A.1** ) reasonably replicate the task participant behavior. As a result, we initialize these parameters with a small number and then allow the E-M algorithm to drive them to their optimal values. The $c$ parameter, defined in (A.6.d), is set to 0.5 in the script and then updated by being learned from each subject's data. The value of 0.5 for $c$ corresponds to a balanced Q- and Actor-critic learning; a larger $c$ favors Q-learning and vice versa. We define a covariance matrix for $W_{k}$ using *Param.Wk*. This is set to be a diagonal matrix with a small variance, because the terms are independent of each other and there is an overall assumption that we can predict behavior accurately if these variables are known. Values of *Param.Ek* are set to either 0 or 0.5; it is set 0 for index 1, 3, 5, and 7 and it is set to 0.5 for index 2, 4, 6, 8 and 9. Note that *Param.Ek* is fixed and it is not adjusted in *compass_em.* The settings suggest that a mixture model (actor-critic and learning) is being trained in the model. If we set *Param.Ek*(9) to 0, we then have a model solely based on actor-critic. If we set all elements of *Param.Ek* to zero except *Param.Ek*(9), we then build a model based on Q-learning. The input vectors *Ib* and *Uk* represent the stimulus information and obtained reward on each trial for the subject being analyzed.

The behavioral signal analyzed in Gold et al. is solely the action – or decision –taken on each trial. However, there is also a reaction time signal, which might carry extra information about the learning evolution or attribute, as in the Prerau et al. papers discussed in section 4. For instance, we might expect to see a shorter reaction time as the correct actions are learned and the subject gains confidence in his/her decisions. It would be straightforward to add reaction time to the model as a function of the learning variables, $X_{k}$, or to add the previous decision outcome as a history term. We can define how the reaction time is linked to the model state variables or input in the function *compass_create_state_space*, and pass both continuous and discrete inputs to the *compass_em* routine. We can also define how parameters of the reaction time model will be trained using *compass_set_learning_param*. We even can define the censoring criteria if some data points are censored due to long response times, *compass_set_censor_threshold_proc_mode*.

**Figure A.3** Analysis script for the hybrid learning task

%% Load Reinforcement Learning Data

% data: In, RESP, dGain, Type, List, Gain

% There are 160 trials in total

% In: 160x4, each column corresponds to one of four different states

% ACC: 160x1, it is the accuracy (correct/ Incorrect)

% dGain: 160x1, it is the reward received per each trial

% Type: 160x1, it indicates loss-win trials (1 is for the win trials, and -1 is for the loss trials)

% List: 160x1, it show the trial state - it matches to In

% Gain: 160x1, is the cumulative gain

T = csvread('HNS2.csv',1,1);

dGain = T(:,8);

ACC = T(:,3);

List = T(:,6);

In = zeros(160,4);

In(find(List==1),1)=1;

In(find(List==2),2)=1;

In(find(List==3),3)=1;

In(find(List==4),4)=1;

%% Set Behavioral Model

% create model

% - 9 state variables, 2 state variables per stimulus plus one for action

% - 4 input to the state process, reward per stimulus

Param = compass_create_state_space(9,4,0,9,eye(9,9),[],[],[1 2 3 4 5 6 7 8 9],[0 0 0 0 0 0 0 0 0]);

RATE_A = 0.1;

RATE_B = 0.1;

RATE_C = 0.1;

% set initial value for Wv,s1 to RATE_A

Param.Ak(1,1)= 1-RATE_A;

Param.Ak(2,1)= 1; Param.Ak(2,2)= -RATE_B;

% set initial value for Wv,s2 to RATE_A

Param.Ak(3,3)= 1-RATE_A;

Param.Ak(4,3)= 1; Param.Ak(4,4) = -RATE_B;

% set initial value for Wv,s3 to RATE_A

Param.Ak(5,5)= 1-RATE_A;

Param.Ak(6,5)= 1; Param.Ak(6,6)= - RATE_B;

% set initial value for Wv,s4 to RATE_A

Param.Ak(7,7)= 1-RATE_A;

Param.Ak(8,7)= 1; Param.Ak(8,8)= - RATE_B;

% set initial value for Wq,a1 to 1

Param.Ak(9,9)= 1-RATE_C;

% set initial value of B

Param.Bk(1,1) = RATE_A;

Param.Bk(2,1) = RATE_B;

Param.Bk(3,2) = RATE_A;

Param.Bk(4,2) = RATE_B;

Param.Bk(5,3) = RATE_A;

Param.Bk(6,3) = RATE_B;

Param.Bk(7,4) = RATE_A;

Param.Bk(8,4) = RATE_A;

Param.Bk(9,1:4) = RATE_C;

% set Param.Wk

Param.Wk = 0.01 * eye(9,9);

% Set Param.Ek

Param.Ek(:) = 0.5;

Param.Ek(1) = 0;Param.Ek(3) = 0;Param.Ek(5) = 0;Param.Ek(7) = 0;

%% Set Learning Procedure

Iter = 1000;

Param = compass_set_learning_param(Param,Iter,3,1,1,1,1,1,0,2,0);

%% Format the Data

% Yb

Yb = ACC;

% Ib

Ib = zeros(length(Yb),9);

Ib(find(In(:,1)),2) = 1;

Ib(find(In(:,2)),4) = 1;

Ib(find(In(:,3)),6) = 1;

Ib(find(In(:,4)),8) = 1;

Ib(:,9) = 1;

% all data points are valid

valid = ones(length(Yb),1);

% Uk - is the reward

Uk = zeros(length(Yb),4);

Uk(:,1)= dGain.*Ib(:,2);

Uk(:,2)= dGain.*Ib(:,4);

Uk(:,3)= dGain.*Ib(:,6);

Uk(:,4)= dGain.*Ib(:,8);

%% Run learning with Binary model

[XSmt,SSmt,Param,XPos,SPos,ML,~,Pb]=compass_em([0 1],Uk,[],Ib,[],Yb,Param,valid);

**Appendix D:** Censored Data Problem Definition

We assume the in-attention state follows a random-walk model – $x_{k}$, $k=1\ldots K$. Reaction time per each trial is defined as a linear function of the state variable plus an additive noise – $y_{k}$. Reaction time is censored if it goes above threshold - $T$. Under this modeling assumption, the state and observation model are defined by

$x_{k+1}=x_{k}+\nu_{k} v_{k}\sim N(0,s_{v}^{2})$ (A.7.a)

$y_{k}=x_{k}+w_{k} v_{k}\sim N(0,s_{w}^{2})$ (A.7.b)

$o_{k}=\left\{ \begin{matrix} y_{k} & y_{k}<T \\ \mathrm{censored} & y_{k}\geq T \end{matrix} \right.$ (A.7.c)

where, the model free parameters include $s_{v}^{2}$ and $s_{w}^{2}$/ Figure A.4 shows Matlab code that generates 200 samples of the model described in equation set (7).

By running this code, we can generate a trajectory of the state and observation process. Figure 4.a and 4.b in example 3 shows the sample simulation result for $T=1.35$. Note that by changing the threshold level, we can increase/decrease the number of censored data points.

**Figure A.4** Matlab script to generate the simulation data

%% Here, we generate a trjaectory of state varibale and observation

% state noise variance

sx = 0.03;

% observation noise variance

sy = 0.09;

% number of samples

K = 200;

% first, create state variable

x = zeros(K,1);

y = zeros(K,1);

x0 = sqrt(sx)*randn();

for k=1:K

if k==1

x(k)=x0 + sqrt(sx)*randn();

else

x(k)=x(k-1) + sqrt(sx)*randn();

end

end

% second, create observation signal

for k=1:K

y(k)=x(k) + sqrt(sy)*randn();

end

%% data stimulation output

% x plus x0 are state variables

% y is the RT

We then use COMPASS to estimate the model parameters and state variable. Figure A.5 shows the analysis script to estimate the state variable and model parameters using full-likelihood method. Here, COMPASS learning is set to estimate both model parameters. The state and parameter estimation result for different threshold levels are shown in Figure 5 of Example 3. COMPASS manual provides a further explanation on how to run different estimation methods for state and parameter estimation in the dataset with different number of censored data points.

%% Here, we treat censored using full likelihood method

% set threshold

Th = 1.5;

% set model input

In = ones(K,1);

Yn = min(y,Th);

% ser obs_valid input

ind = find(y>=th);

obs_valid = ones(K,1);

obs_valid(ind)=2;

%% Set behavioral model structure and learning parameters

Param = compass_create_state_space(1,0,1,0,1,1,0,[],[]);

% set learning parameters

Iter = 100;

Param.Ck = 1;

Param.Dk = 1;

Param = compass_set_learning_param(Param,Iter,0,0,0,0,1,1,1,2,1);

% define censored point threshold and estimation technique (set as likelihood)

Param = compass_set_censor_threshold_proc_mode(Param,th,2,1);

%% Run EM step: state and parameter estimation

[XSmt,SSmt,Param,XPos,SPos,ML,Yp]=compass_em([1 0],[],In,[],Yn,[],Param,obs_valid);

**Figure A.5** Analysis script for the state and model parameter estimation – here, we assume the data is first generated using Matlab script presented in Figure A.4. Note we can set threshold by checking the maximum of y variable.
