# Supplementary material for: COMPASS: An Open-Source, General-Purpose Software Toolkit for Computational Psychiatry
Source: Front Neurosci. 2019 Jan 11;12:957. doi: 10.3389/fnins.2018.00957 (PMC6336923; doi:10.3389/fnins.2018.00957)
Supplement: Supplementary file 2 [file Data_Sheet_2.docx]

**Program Manual for State-Space Modeling Toolbox (COMPASS)**

Oct 2018

In this manual, we start with a sample problem which describes the pipeline of processing using the toolbox functions. We then provide a detailed explanation for each function used in this example.

**Example Problems**

The data file in this example is a mat file called LEARNING_1.mat which can be found in the [GitHub](https://github.com/Eden-Kramer-Lab/COMPASS) repository. This file includes the behavioral outcome per trial, including both reaction time and binary (correct/incorrect) decisions. The data come from an associative learning task, where through the task, a rhesus macaque learns the correct response on each trial to receive reward. For this task, we are interested in analyzing how the animal learns over time. The source code for this example is called compass_example_step_by_step.m and can be found in the [GitHub](https://github.com/Eden-Kramer-Lab/COMPASS) folder.

We can import the data by calling the following lines:

**%% Load behavioral data and prepare it for the toolbox**

**% Data: Yb (decision), Yn (reaction time)**

**load('LEARNING_1.mat');**

**% Yn - logarithm of reaction time in seconds**

**Yn = log(Yn/1000);**

**% Data length**

**N = length(Yn);**

**% Input - 1 xi**

**In = zeros(N,2);In(:,1)= 1; In(:,2)= 1;**

**% Input, Ib is equal to In**

**Ib = In;**

**% Uk, which is all zero**

**Uk = zeros(N,1);**

**% valid, which is valid for the observed data point**

**ind = find(isnan(Yn));**

**Valid = ones(N,1);**

**Valid(ind)=0;**

Figure 1 shows the reaction time and correct/incorrect response per trial:


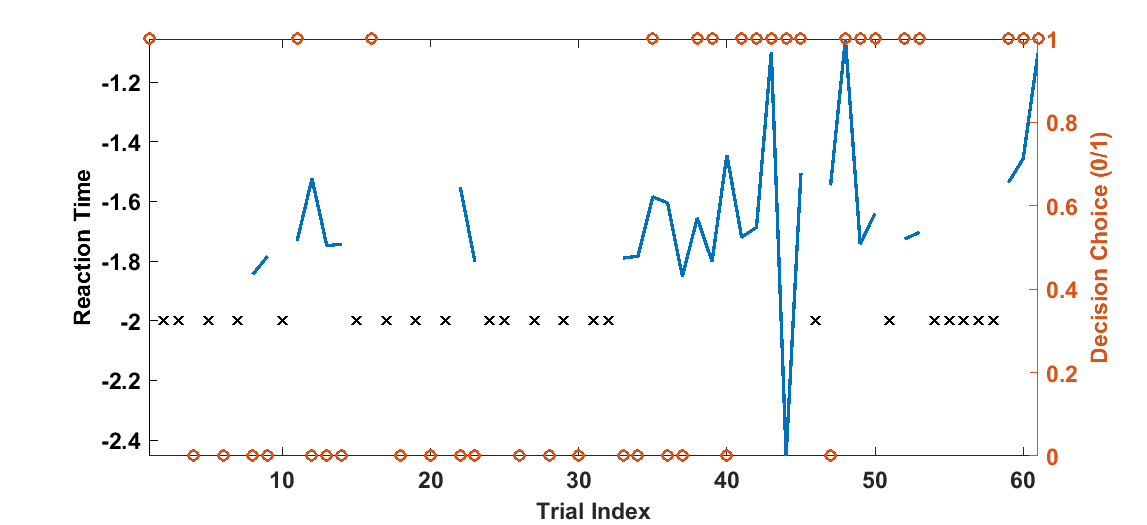


**Figure 1** Reaction time and choice per trial. Trials where no response was made are marked by ‘x’.

There is a clear trend over time towards more correct responses, showing that the animal learns the task. It also shows as the animal learns the task, it requires a longer time to pick the correct choice. Our hypothesis is that the underlying mental processes – the learning state – is projected on both the reaction time and the decision. Our goal is to infer the underlying learning state through the behavioral outcome. We also assume that in the missed trials, the animal is not focusing on the task, and as result, these points don’t carry information about the learning state. To model this task, we assume that the learning state evolution over trials can be described by a random walk model.

$x_{k+1}=x_{k}+w v\sim N(0,\sigma_{w}^{2})$ (1)

where, $x_{k}$ is the learning state over trial $k.$ The noise term allows the state to change from one trial to the next. The observation per each trial is defined by $z_{k}=\{\left( y_{c,k},y_{b,k} \right),^{'}Missed trial'\}$. The observation model for observed trials is defined by

$p(y_{b,k}=1)={\exp(x_{k}+c_{0})}/{(1+\exp(x_{k}+c_{0}))}$ (2.a)

$\log y_{c,k}=b_{1} x_{k}+b_{0}+v v\sim N(0,\sigma_{v}^{2})$ (2.b)

where, $y_{b,k}$ is decision choice per trial. $y_{b,k}$ is either 1 which means a correct choice or 0 which means an incorrect choice. $y_{c,k}$ is the reaction time which is defined in seconds. We assume that probability of a correct choice follows a Bernoulli process and can be defined by a sigmoid function. Given the model definition, as $x_{k}$ increases, the probability of picking the correct choice increases. $c_{0}$ defines the probability of picking the correct choice in the absence of the learning state; this might be biased and it is not necessarily always 50/50. We also assume that the reaction time can be defined by a log-normal distribution, and thus that the logarithm of reaction time is defined by a linear function of the learning state. $b_{0}$ defines the reaction time baseline and $b_{1}$ defines the slope of reaction time as a function of the learning state. Given the reaction time trend over trials (increasing with learning), we expect $b_{1}$ to be a positive number and $b_{0}$ to be negative. We also model the reaction time variability - or the other unknown mechanism in reaction time - using an additive white noise $v$. The toolbox helps us to estimate the model parameters - $c_{0}, b_{1}, b_{0}, \sigma_{v}, \sigma_{w}$ – along with the learning state $x_{k}$. Note that there is no scaling term (which might be called $c_{1}$ if it were present) for $x_{k}$ in the Bernoulli process. Adding this term would lead to a model that is too complex and not identifiable. $x_{k}$ appears in both the reaction time and decision choice formulas. If we have $c_{1} x_{k}$ and $b_{1} x_{k}$ terms rather than $x_{k}$ and $b_{1} x_{k}$, then we have infinitely many choices for $x_{k}$, $c_{1}$ and $b_{1}$- for example, $\alpha^{-1} c_{1} \alpha x_{k}$ and $\alpha^{-1} b_{1} \alpha x_{k}$ are valid for any nonzero values of $\alpha$.

Now that we have the model definition, we can implement the model using the COMPASS toolbox. The first step is defining the structure of the model. The following line in the code builds the model

**%% Build behavioral model and learning procedure**

**% create model**

**Param = compass_create_state_space(1,1,2,2,1,1,1,1,0);**

Look at the compass_create_state_space function description to see each of its input argument definitions and how they are set in this example. We have one state, so nx is 1. We have no inputs (trial types or external perturbations), so we set nUk to 1, and we set all of Uk's elements zero in the code above. This corresponds to the random walk definition of the learning state. We also assume In and Ib dimensions (nIn and nIb) are 2 and the elements of Ib and In are all set to 1. This implies the specific form of the observation process defined in equation (2). The COMPASS code assumes models of the form:

$\log y_{c,k}=b_{1} In(k,1) x_{k}+In(k,2) b_{0}+v$ (3)

and

$p(y_{b,k}=1)={\exp(Ib\left( k,1 \right) x_{k}+Ib\left( k,2 \right) c_{0})}/{(1+\exp(Ib\left( k,1 \right) x_{k}+Ib\left( k,2 \right) c_{0}))}$ (4)

that is, why nIn and nIb are set to 2 in this example. To have our specific model as defined in equation set (2), we set all elements of Ib and In to 1.

We also address the identifiability issue by setting the last argument of the function to 0, which implies the specific form of discrete observation process- $c_{1}=1$. Note that, a more general form of binary observation is defined by

$p(y_{b,k}=1)={\exp(c_{1} x_{k}+c_{0})}/{(1+\exp(c_{1} x_{k}+c_{0}))}$ (5)

where, we set $c_{1}$ to 1. Note that it is also possible to fix $b_{1}$ at 1 and let $c_{1}$ be adjusted by the model. If we use this definition, the interpretation of the learning state might change given the model parameters. Here, we have set dLinkUpdate to 0 and cLinkUpdate to 1 – see compass_create_state_space for the definition of these parameters. We can switch these values if we want to set $b_{1}$ at 1 and have $c_{1}$ learned from data instead.

Now that we have defined the model using compass_create_state_space, we can define the model learning rule. The following lines in the code define the learning setup.

**% set learning parameters**

**Iter = 250;**

**Param = compass_set_learning_param(Param,Iter,0,1,0,1,1,1,1,1,0);**

We can follow the compass_set_learning_param function description to see each of its input argument definition and how they are set in this example. In this example, we set the number of EM iterations to 250. These input arguments also cause the state-transition parameters to be fixed and not learned from data. Note that, the general structure of the state transition model is:

$x_{k+1}=a x_{k}+b*u_{k}+w w\sim N(0,\sigma_{w}^{2})$ (6)

where here, $a$ is set to 1 and is not updated. That is, $x_{k}$ does not decay or increase on its own. If we wished to model some form of forgetting or imperfect working memory, we might set *a* to some number slightly less than 1. We already set $u_{k}$ to zero. The arguments above cause the noise term to be updated based on data, and we assume $x_{0}$ is fixed and not updated here. Also, the input arguments suggest that other parameters of the model will be adjusted during the training. This setting is in accordance with our model specification, and it can be changed if the structure of the model or our assumption about the model parameters will change. The last term is set to 0, and would only be non-zero in models using gamma distributions.

Finally, the data we are using, like many other datasets, contain missing observations. These are not necessarily missing at random – they might indicate times when the animal did not decide before a timer expired. We need to identify these "censored" points for the algorithm and tell it what to do with them. We call the following function to define the censoring threshold and the methodology to address censored data points in the data.

**% define censored point threshold**

**Param = compass_set_censor_threshold_proc_mode(Param,log(2),1,1);**

That is, we define trials above 2 seconds to be censored, and would attempt to impute them by a single imputation draw. However, above (and below), we set elements of obs_valid as 0, implying they are missing at random. Those data will not be imputed regardless of this function's settings. If we instead set elements of obvs_valid to 2, the imputation specified in this function will take effect.

**% valid, which is valid for the observed data point**

**ind = find(isnan(Yn));**

**Valid = ones(N,1);**

**Valid(ind)=0;**

Note that we have defined the data attributes when the data were loaded. We define a **Valid** vector in the data loading process, which carries information about the data. The elements of **Valid** is 0, 1, or 2. An observed data point comes with **Valid** value of 1. Value 0 means a MAR data point and 2 means a censored data point. Note that the Value elements for dropped data points are set to 0. If we set **Valid(ind)=2**, then we assume the data on these trials are censored with a time threshold of 2 seconds. For other arguments check compass_set_censor_threshold_proc_mode definition.

Note that the model definition and learning information are all kept in the **Param** structure. The definition of **Param** elements can be found in the following pages.

Till this point, we have defined the model structure in COMPASS and we have set the learning rule. We have prepared data for the toolbox and set its attributes. The next step is to call the COMPASS main function, **compass_em.**The following line of code calls the function

**%% Run learning with a mixture of normal & binary**

**[XSmt,SSmt,Param,XPos,SPos,ML,YP,YB]=**

**compass_em([1 1],Uk,In,Ib,Yn,Yb,Param,Valid);**

The function input includes the **Param,** type of observation process, and the data observations. Note that in this example, we utilize both continuous (reaction time) and discrete observations in estimating the learning state. This corresponds to setting the DISTR argument to **[1 1]**. We might change this argument to other values if we change our assumption about the observation process. For example, if we set the argument to [0 1], then the learning will be solely based on the discrete observation. The other inputs to this function are read directly from the data, except for Valid, which is set as we described above.

The function output is our learning state of interest. It returns forward-only filter (XPos, SPos) and forward-backward smoother (XSmt, SSmt) estimates of the state variables plus updated parameters. Besides that, we get the model likelihood at each iteration (ML) and the expected reaction time and probability of correct choice per trial (YP,YB). These are the core pieces of information that shape our inference about the data and underlying state variables. Figure 2 shows the filter estimate for the learning state with its confidence bound.


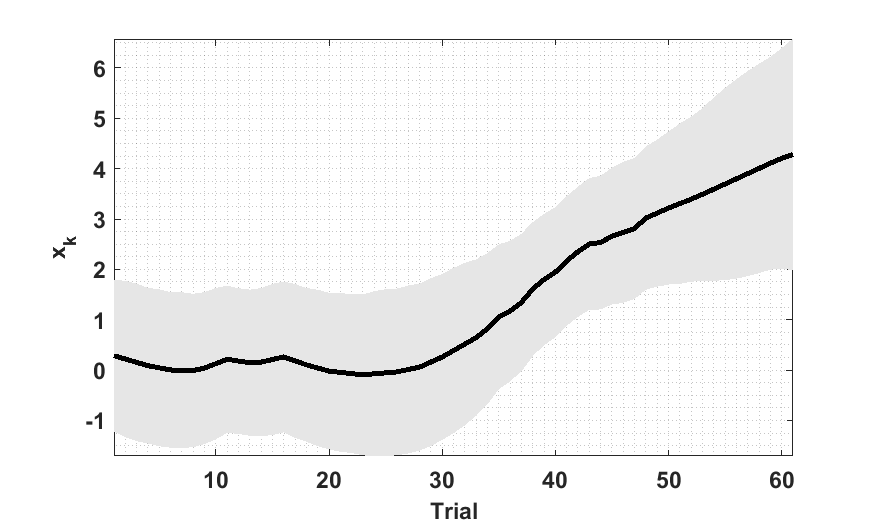


**Figure 2** Posterior estimate of $x_{k}$. The $c_{0}$ is -1.3874; the sum of $c_{0}$ and $x_{k}$ determines the probability of a correct choice per trial. The $b_{1}$ is 0.0479, which defines how the reaction time increases over trials. The $x_{1}$ is about 4 at trial 60, suggesting that the reaction time is about 20% higher as the animal proceeds through a block.

Figure 3 shows the ML estimate over 250 trials. We generally expect an increasing ML curve over trials, although this might not be always true depending on the approximations included in the model and choice of initial value of the model. To increase the likelihood of increasing ML (convergence), we can set initial values of the model parameters. These can be derived using a static generalized linear model (GLM) or similar analysis. Initial values for the model parameters are:

**Param.Wk = 0.2;**

**Param.Ck = 0.1;**

**Param.Dk = [0 -1.66];**

**Param.Vk = 0.05;**

**Parm.Fk = [0 -.15];**

Default values of these parameters are provided in compass_create_state_space; however, these setting might not be optimal for all data sets. Here, we describe how better initial values can be derived for these parameters and for modeling analyses generally.

We can see that the animal learns the task as trials go by, and that this corresponds to an increasing $x_{k}$ over trials. Note that the probability of correct choice increases as $x_{k}$ increases. On the other hand, we set the initial value of $x_{0}$ to zero. Thus, we expect $x_{k}$ to be a positive variable specifically at the end of the experiment. On the other hand, we observe that most of the choices are incorrect at the beginning of the task. Thus, this suggests $c_{0}$, which is Parm.Fk(2), should be negative. In this experiment, there are 19 correct responses out of 39 trials (with a response). We can thus set the initial values of $c_{0}$ with a number that represents 19/39~48% accuracy. Thus, we set Parm.Fk(2) to -0.15 - $\log\left( \frac{p}{1-p} \right), p=0.462$ which is about this percentage of correct response. You can check Param definition to find how Parm.Fk(2) relates to $c_{0}$. We can take a similar rationale for the reaction time model. Mean of Yn , the reaction time, is -1.66, and thus we can set $b_{0}$, which is Param.Dk(2), to this number. Yn variance is about 0.05, and thus, we can set Param.Vk to this number. We know $x_{k}$ grows over trials, and so does reaction time. This suggests that $b_{1}$, which is Param.Ck, should be a positive number. We see from the choice data, that the animal responds with an accuracy of 90% or more, which corresponds to $x_{k}$ of 2 or 3. Thus, we might assume $x_{k}$ is 3 at the end of the task, and by checking reaction time at the end of the task, which is about -1.4, we set $b_{1}$ to 0.1. The last parameter is Param.Wk. We assume that variability of the behavior might have two sources. There can be variability in the response itself or in the underlying state (learning state). We can see the assumed variability in reaction time is about 0.05, and there might be another variability which is generated by $x_{k}$. Note that we set $b_{1}$ to a small number, 0.1 here, which suggests $x_{k}$ variance should be larger in order to generate reaction time variability. We can also assume that the variability of $x_{k}$ be large enough that the E-M models allows it to change over trials. As a result, we keep $x_{k}$'s variance larger than Param.Vk and set to 0.2, Param.Wk = 0.2. Note that these are not necessarily the only choice for these parameters. The reasoning here might be useful, however, in defining other behavioral models.


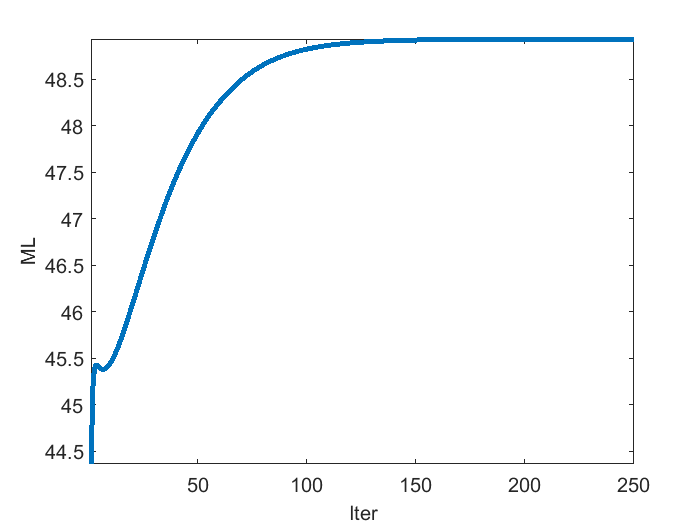
Figure 3 shows the ML curve over 250 iterations. The curve reaches a plateau, which corresponds to a local maximum. Note that, if we start with another initial setting different from the setting used here, we might converge to another local maximum. In general, we are interested in a set of parameters and state estimation which lead to a meaningful description for the model we have built. For instance, we build the model by the assumption that learning state ($x_{k}$) increases over the experiment and we can see that the model output is aligned with our hypothesis.

**Figure 3** ML curve over 250 trials. The curve reaches a plateau which corresponds to a local maximum*.*

By running compass_em, we get the posterior estimate of the state variable (XPos, SPos) and updated model parameters. We get an ML curve as shown in Figure 3. The final output is the expected reaction time and probability of choice per trial (YP,YB). This can be used to check the model's fit to the data, in terms of whether it is able to predict the observed output. Note that in the example, we have MAR points and we estimate expected the reaction time and probability of correct choice on those trials too. Figure 4 show the expected reaction time and probability of correct choice per trial.


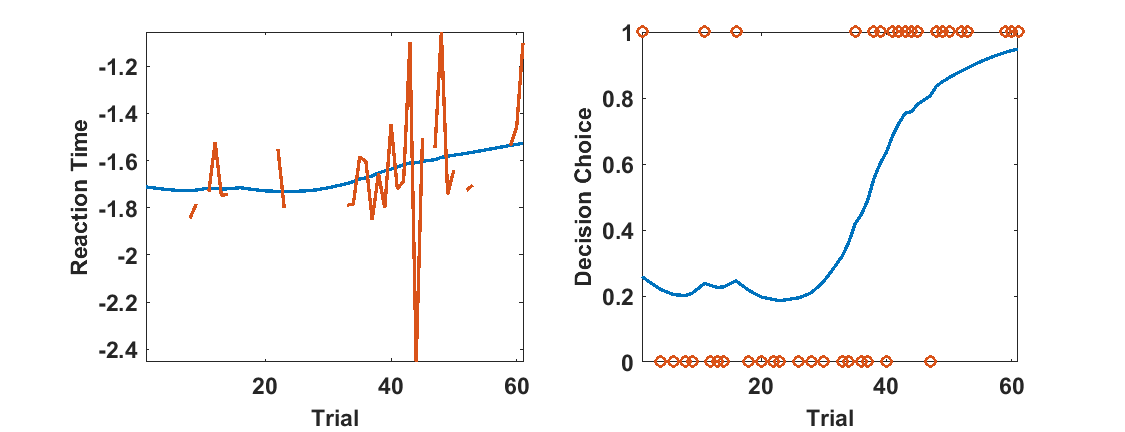


1. (B)

**Figure 4** Expected reaction time (Panel A, blue) and expected probability of correct choice (Panel B, blue). Note that because the state evolves smoothly through time, we have estimated reaction times and choices even on trials where the subject did not respond. (A) The predicted reaction time (blue) follows the central tendency of the observations (orange), effectively acting as a smoothed version of the reaction time. In Figure 2, we can see that the confidence interval of $x_{k}$ grows at the end of experiment along with the $x_{k}$ growth, and this might reflect the larger variability in the reaction time at the end of the experiment. (B) The decision prediction (blue) suggests that probability of correct choice grows from around 20% to above 90%. The dot points (Panel B, orange) show correct/incorrect choice per each trial. A value of zero means an incorrect choice and one means a correct choice being made on that specific trial.

If we are considering multiple models, we can take further steps to compare models or assess the parameters estimation. For instance, we can call compass_deviance to estimate deviance over the continuous and discrete observation processes. Deviance is a goodness-of-fit statistic for a statistical model. It is a generalization of the idea of using the sum of squares of residuals in ordinary least squares fitting problems (Reference 4 – Ch2; Reference 5 -Ch7).

**%% Deviance analysis**

**[DEV_C,DEV_D]= compass_deviance([1 1],In,Ib,Yn,Yb,Param,Valid,XSmt,SSmt);**

The inputs to this function are the outputs and/or inputs of compass_em. We can use the deviance result to assess goodness-of-fit across different models (Reference 5 -Ch7). For instance, in modeling the learning behavior, we might assume that the reaction time (RT) is dependent on the trial number. Whether this component is related to RT can be checked by the changes in the deviance – in other words, changes in the deviance carry information about the significance of this new component in predicting RT. Naively speaking, a smaller deviance corresponds to a better fit to the data. Note that in the deviance measurement, there is no penalty term for the number of free parameters or the dimension of the state variable. That type of penalty term is often used to avoid over-fitting a model to data (Reference 6). Let’s assume we want to compare two other models to the model we built so far. First, we consider a model where the correct/incorrect choice depends only on a fixed parameter $c_{0}$ that is unaffected by learning:

$p(y_{b,k}=1)={\exp(c_{0})}/{(1+\exp(c_{0}))}$ (7)

There are still both continuous and discrete observations, but only the continuous observation is now linked to the state. Model parameters, including $c_{0}$ , are learned from data. The code for this model is

**Param.Wk = 0.2;**

**Param.Ck = 0.1;**

**Param.Dk = [0 -1.66];**

**Param.Vk = 0.05;**

**Param.Ek = 0;**

**Parm.Fk = [0 -.15];**

**[XSmt,SSmt,Param,XPos,SPos,ML,YP,YB]=**

**compass_em([1 1],Uk,In,Ib,Yn,Yb,Param,Valid);**

**[DEV_C1,DEV_D1]=**

**compass_deviance([1 1],In,Ib,Yn,Yb,Param,Valid,XSmt,SSmt);**

For the second alternate model, we assume we only have the binary observation. This means the state estimation and model parameter estimation are only derived from the binary observation and the reaction time is not considered. The code implementation is

**Param.Wk = 0.2;**

**Param.Ck = 0.1;**

**Param.Dk = [0 -1.66];**

**Param.Vk = 0.05;**

**Param.Ek = 1;**

**Parm.Fk = [0 -.15];**

**[XSmt,SSmt,Param,XPos,SPos,ML,YP,YB]=**

**compass_em([0 1],Uk,In,Ib,Yn,Yb,Param,Valid);**

**[DEV_C2,DEV_D2]=**

**compass_deviance([1 1],In,Ib,Yn,Yb,Param,Valid,XSmt,SSmt);**

We can see the deviance analysis for these three models (the original and two alternative models) in the following table.

**Table 1** Deviance Comparison Between Different Models

| **Model** | **Deviance (Continuous Obs.)** | **Deviance (Discrete Obs.)** |
| --- | --- | --- |
| Original Model | -7.7 | 36.9 |
| Model with both observations, and without a link to the state in the binary observation | -3.4 | 54.0 |
| Model with binary observation alone | 32.8 | 38.5 |

The deviance result between the original model and the first alternative model suggests that including the state variable in the discrete model will improve our data explanation. This is because we see a big increase in the deviance result on the discrete part. The result also suggests that we might get a better explanation of reaction time in the first alternative model; however, Param.Ck (=0.046) becomes tiny suggesting that the reaction time alone might not carry much information about learning state. In the second alternative method, the deviance has increased a bit for the discrete observation, suggesting that the binary observation alone carries less information about the learning state compared to when both reaction time and choices are utilized in the model.This is in accordance with the original model result. Even though the reaction time on its own does not carry much information about learning, it does carry information when specifically conditioned on/considered in parallel with the subject’s choices. . Note that, in the second alternative model, we don’t tune the continuous observation parameters and thus we get a large deviance. This sort of analysis provides a view about which observation carries more information and how we can tune our model structure. We could further examine other outputs of the alternative models, such as the time course of the estimated learning state and the static model parameters. Now, we will discuss the covariance estimate of the model coefficients which provides an extra information about the model structure.

Another key model verification analysis is the covariance estimate of the model free parameters. For instance, we might be interested in checking the confidence interval for $c_{0}$, $b_{0}$ or $b_{1}$ estimates or the model noise parameter estimation. To find this information, we can call the following function:

**%% Covariance analysis**

**[COV_X,COV_C,COV_D]=compass_param_covariance_info([1**

**1],Uk,In,Ib,Yn,Yb,Param,Valid,XSmt,SSmt);**

Figure 5 shows the confidence interval for the model parameters of our original example model. The result is in accordance with deviance analysis; we can see that $b_{1}$ is close to 0, with a confidence interval almost including 0 –we discussed that RT is less dependent to learning state and decision carries much of information about the learning state . This implies that the reaction time is relatively insensitive to the state. We can see a larger confidence interval for $c_{0}$. The state carries much of the information about the choice decision, and this level of information changes over time. This does suggest that we might want more data to narrow this coefficient estimate. We see a larger variance estimate for the state process $\sigma_{w}^{2}$, shown by Sw, compared to the variance of the observation process $\sigma_{v}^{2}$, shown by Sv.

These two functions along with compass_em output provide a reasonably inclusive picture of the model and state estimation. We can use these information for refining the model structure similar to what is done in static GLM selection (Reference 5- Ch7). This will be similar to a model selection process, when new covariates are added or dropped sequentially. A cautionary point is that no single number or output from these analyses indicates that one model is preferable. Rather, the model is selected based on how well the analysis matches what we know or assume about the problem – our pre-existing hypotheses or domain-specific knowledge.

In this example, we worked over different steps that can be done in COMPASS to build a model and analyze its result. We can use COMPASS to build different models and examine how they might explain our signal.


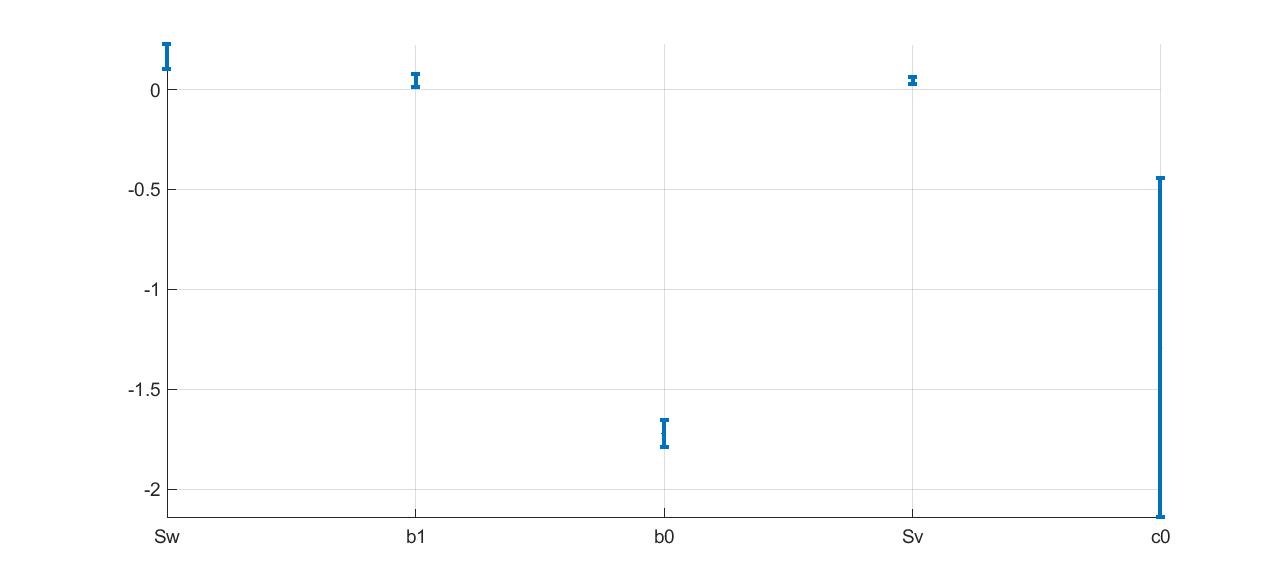


**Figure 5** Confidence interval of the model free parameters. The result suggests that $b_{1}$ might be close to zero, which suggests the reaction time is less dependent to the learning state ($x_{k}$).

**Model Creation Wrapper Function: compass_create_model.m**

**compass_create_model** is a wrapper function combining **compass_create_state_space**, **compass_set_learning_param**, and **compass_set_censor_threshold_proc_mode** to create a set of input arguments to be passed through the **compass_em** function. Using this function, the user can create a state-space model and set learning and threshold parameters easily by utilizing a series of question and answer functions. This allows the user to easily construct a desired model without possessing an in-depth understanding of each toolbox function that is necessary for model construction. The function itself is versatile and will adapt to various user preferences and data types when choosing a model structure, and will furthermore allow the user to run an unlimited amount of models consecutively through **compass_em** without interruption.

Upon initially running this function, the user will be prompted to enter the path and name of a data file containing the following variables. The function assumes that the user has already created this file in mat format, and that the user knows where it is located. If no path is given, the file must be located in the same directory as the function in order to proceed.

**Yn** – Vector of continuous observation data signal, e.g. reaction time. Vector size: **K** x 1, where **K** is the number of observed data points.

**Yb** – Vector of discrete observation data signal, e.g. correct/incorrect response. Vector size: **K** x 1.

**In** – Matrix of continuous observation input. Matrix size: **K** x **nIn**, where nIn is a scalar defining the dimension of input passed to the continuous part of the model.

**Ib** – Matrix of discrete observation input. Matrix size: **K** x **nIb**, where nIb is a scalar defining the dimension of input passed to the discrete part of the model.

**obs_valid** – Vector that determines whether each data point is completely observed (with a value of 1), missing at random (with a value of 0), or censored by threshold (with a value 2). Vector size: **K** x 1. If this variable is not provided in the user inputted data file, it will be created as a vector of dimensions **K** x 1. All values will be equal to “1.”.

**Uk** – Matrix that defines input to state-transition model across time indices. If this variable is not given, it will be created as an empty vector: [].

**NOTE:** The bare minimum variables needed to run this function are a pair of either **Yb** and **Yn**, or **In** and **Ib**. This will allow for processing of continuous variables and/or discrete variables respectively. If neither of these two sets of variables are within the uploaded data file, the function will terminate with the message “*Error: Function cannot be executed due to lack of present variables* Check data file for necessary variables and proper spelling/capitalization.”. The function will account for missing discrete variables as long as there are present continuous variables, and vice versa. Proper model construction for the presence/absence of these variables will be accounted for by the function itself.

Once all model-based questions are answered, the user will finally be prompted to save their new input arguments in a mat-file. The user will be prompted to add a suffix to the given prefixed file-name “em_set.” Then, they will be capable of running **compass_em** easily with these new parameters using the function **compass_run_models** (described below). Using **compass_run_models** in accordance with **compass_create_model** will allow users to run multiple model settings at a time without interruption. After each variable input set is passed using **compass_run_models**, the resulting model information will be saved as a new mat file named **“**model_result” with an added suffix number given by the order in which the variable input set was received by the function. This process will be explained further below at the end of the example problem.

In the following sections, we describe each step of this function by building a particular model including both continuous and discrete observations using **compass_create_model**. This will include an entire run through of the function, spanning question and answer functions regarding **compass_create_state_space**, **compass_set_learning_param**, and **compass_set_censor_threshold_proc_mode.** It should be noted that the nature of questions presented by **compass_create_model** will differ pending the data uploaded by the user, and the answers that the user provides in question and answer functions. This is merely one example of many potential models that can be generated using this function.

**Example Model Using compass_create_model.m Wrapper function**

Let’s assume the user would like to model the following equations:

$${Y_{n}(k)\sim f((2 I}_{n,1}\left( k \right)+\beta_{1} I_{n,2} )x_{1}\left( k \right)+I_{n,3}\left( k \right)\beta_{3};\theta)$$

$${Y_{b}(k)\sim g(I}_{b,1}\left( k \right)x_{1}\left( k \right)w_{1}+I_{b,2}{\left( k \right)x}_{2}\left( k \right)w_{2};\theta)$$

In the above equations, $\boldsymbol{Y}_{\boldsymbol{n}}$ stands for a continuous data signal and $\boldsymbol{Y}_{\boldsymbol{b}}$ stands for a discrete data signal.$\boldsymbol{I}_{\boldsymbol{n}}$ and $\boldsymbol{I}_{\boldsymbol{b}}$ stand for input of continuous and discrete observations respectively. $\boldsymbol{x}_{\boldsymbol{1}}\boldsymbol{(k)}$ and $\boldsymbol{x}_{\boldsymbol{2}}\boldsymbol{(k)}$ represent model state variables, while $\boldsymbol{\beta}$ and $\boldsymbol{w}$ represent model parameters. $\boldsymbol{\theta}$ represents remainder parameters of the model, including noise terms. Note that for the continuous model, we can assume a different distribution model, which will be one of the questions being called in the function. For discrete observation, we assume $\boldsymbol{Y}_{\boldsymbol{b}}\boldsymbol{(k)}$ follows a Bernoulli distribution$,\mathrm{thus} \boldsymbol{Y}_{\boldsymbol{b}}\boldsymbol{(k)}$ will have a value of 0 or 1 at each time index. Note that, in the continuous model, the coefficient of the first term is fixed – equal to 2 – for $\boldsymbol{I}_{\boldsymbol{n,1}}\boldsymbol{(k)}$ while other parameters described **($\boldsymbol{\beta}_{\boldsymbol{1}}\boldsymbol{,}\boldsymbol{w}_{\boldsymbol{1}}\boldsymbol{,}\boldsymbol{w}_{\boldsymbol{2}}$)** are assumed to be free. The toolbox has the capability of handling this and it will be described below through the questions being asked by the function. Below, it is assumed that a mat file containing **Yn**, **Yb**, **In**, **Ib**, **obs_valid**, and **Uk** is being loaded. The following answers will generate these two equations, as well as prepare a series of input arguments necessary to be passed through **compass_em**:

1. **Please provide the exact path to your mat variable input file:**

Here, the user will provide the exact path and name of their mat variable input arguments.

Ex. G:\MATLAB\COMPASS_StateSpaceToolbox\example_data.mat

1. **Do you have continuous observations? (Y/N)**

“N” would exclude continuous observations from the model, which involves variables In and Yn.

Answer “Y” because there is an equation involving continuous observation ($\boldsymbol{Yn}$).

1. **Do you have discrete observations? (Y/N)**

Answer “Y” because there is an equation involving discrete observation ($\boldsymbol{Yb}).$

1. **Which distribution for continuous observations? (If answered “Y” to continuous observations)**

Picking 2 would result in your model following a Gamma distribution.

Pick 1 to have your model follow a normal distribution.

1. **How many state variables do you have?**

Answer “2” because there are two state variables, $\boldsymbol{x}_{\boldsymbol{1}}\boldsymbol{(k)}$ and $\boldsymbol{x}_{\boldsymbol{2}}\boldsymbol{(k)}$ .

*CONTINUOUS STATE VARIABLES*

1. **Which state variable will be linked to column number “1” in your In matrix?**

Answering “0” will indicate that no state variable will be linked to this column.

Answer “1” because state variable $\boldsymbol{x}_{\boldsymbol{1}}\boldsymbol{(k)}$ is linked to column number 1 in the In matrix ($\boldsymbol{I}_{\boldsymbol{n}}$), following the above equation

1. **Is this a free or fixed parameter? ( In(1)* b *x1) Enter 1 for free, 0 for fixed:**

Answer “0” for fixed because there is no changing free parameter associated with the $\boldsymbol{I}_{\boldsymbol{n,1}}$ term.

1. **What is the initial value of the parameter?**

Answer “2” because the $\boldsymbol{I}_{\boldsymbol{n,1}}$ term has a coefficient of 2.

1. **Which state variable will be linked to column number “2” in your In matrix?**

Answer “1” because $\boldsymbol{x}_{\boldsymbol{1}}\boldsymbol{(k)}$ is linked to column number 2 in the In matrix ($\boldsymbol{I}_{\boldsymbol{n}}$).

1. **Is this a free or fixed parameter? ( In(2)* b *x1) Enter 1 for free, 0 for fixed:**

Answer “1” for fixed because there is a changing $\boldsymbol{\beta}_{\boldsymbol{2}}$ variable associated with the $\boldsymbol{I}_{\boldsymbol{n,2}}$ term.

1. **What is the initial value of the parameter?**

This value is up to the user, let’s say it is around 1.2.

1. **Which state variable will be linked to column number “3” in your In matrix?**

Answer “0” because there is no state variable linked to column number 3 in the In matrix ($\boldsymbol{I}_{\boldsymbol{n}}$).

1. **Is this a free or fixed parameter? ( In(3)* b) Enter 1 for free, 0 for fixed:**

Answer “1” for free because there is a changing $\boldsymbol{\beta}_{\boldsymbol{2}}$ variable associated with the $\boldsymbol{I}_{\boldsymbol{n,3}}$ term.

1. **What is the initial value of the parameter?**

This value is up to the user, let’s say it is around 1.4

**CONTINUOUS MODEL: Yn~f(In(1)*2*x1)*(In(2)*(b1->1.2)*x1)*(In(3)*b2->1.4)**

**Press enter to continue**

***DISCRETE STATE VARIABLES***

1. **Which state variable will be linked to column number “1” in your Ib matrix?**

Answer “1” because $\boldsymbol{x}_{\boldsymbol{1}}\boldsymbol{(k)}$ is linked to column number 1 in the **Ib** matrix ($\boldsymbol{I}_{\boldsymbol{b}}$).

1. **Is this a free or fixed parameter? ( Ib(1)* b *x1)Enter 1 for free, 0 for fixed:**

Answer “1” for free because there is a changing $\boldsymbol{w}_{\boldsymbol{1}}$ variable associated with the $\boldsymbol{I}_{\boldsymbol{b,1}}$ term.

1. **What is the initial value of the parameter?**

This value is up to the user, let’s say it is around 1.2.

1. **Which state variable will be linked to column number “2” in your Ib matrix?**

Answer “2” because $\boldsymbol{x}_{\boldsymbol{2}}\boldsymbol{(k)}$ is linked to column number 2 in the **Ib** matrix ($\boldsymbol{I}_{\boldsymbol{b}}$).

1. **Is this a free or fixed parameter? ( Ib(1)* b *x1) Enter 1 for free, 0 for fixed:**

Answer “1” for free because there is a changing $\boldsymbol{w}_{\boldsymbol{2}}$ variable associated with the $\boldsymbol{I}_{\boldsymbol{b,2}}$ term

1. **What is the initial value of the parameter?**

This value is up to the user, let’s say it is around 1.4.

**DISCRETE MODEL: Yb~g(Ib(1)*(w1->1.2)*x1)*(Ib(2)*(w2->1.4)*x2)**

**Press enter to continue**

***Learning Parameters Update***

Enter “1” for “yes”, or “2” for “no” *UNLESS OTHERWISE SPECIFIED*

1. **Update state-transition model parameters?**

A value of 1 updates elements of **A** and **B** matrices, whereas a value of 0 will not update these matrices.

Let’s choose a value of 1.

1. **Update state-transition model covariance matrix?**

A value of 1 updates **W**, the covariance matrix. A value of 0 will leave the **W** matrix un-updated.

Let’s choose a value of 1.

1. **Update initial state variable parameters?**

A value of 1 updates state variable parameters, the mean and covariance matrices, at time 0. A value of 0 will leave these parameters un-updated, and the user can manually initialize variables **Param.X0** and **Param.W0**.

Let’s choose a value of 1.

1. **Update continuous parameters?**

A value of 1 will update the continuous observation process during EM training. A value of 0 will leave these parameters un-updated. If continuous observations are not present, this question will be skipped.

Continuous observations are present, so we can choose a value of 1.

1. **Update noise-term?**

A value of 1 will update the noise-term during the EM training process. In continuous observation processes with a normal or Gamma distribution, the noise term represents additive normal white noise. Whereas in continuous processes with a Gamma distribution, the noise term corresponds to a dispersion term. If continuous observation is not present, this question will be skipped.

Continuous observation is present, so we can choose a value of 1.

1. **Update discrete parameters?**

This question and answer choices are identical to question #24 (update the observation process during EM training) but for the discrete observation process. If discrete observations are not present, this question will be skipped.

Discrete observations are present, so we can choose a value of 1.

1. **Is matrix “A” in state-transition process diagonal or not?**

A value of 1 will make the A matrix in the state transition process model diagonal, which is the favorable choice for EM learning. A value of 0 will make the A matrix a full matrix, which could lead to less accurate or unstable estimations of both parameters and state-variables.

Let’s choose a value of 1.

1. **Update mean before covariance (ENTER 1), or covariance before mean (ENTER 2)?**

For the filter mean and covariance update, this determines if mean is updated before covariance (a value of 1), or vice versa (a value of 2). Further information about this topic can be found in Reference 3.

Let’s choose a value of 1.

1. **Update a positive shift estimation during the training process?**

A value of 1 sets the estimation of a positive shift as part of the EM training process, updating this estimation during training. A value of zero will make this shift parameter pre-set and un-updated in the EM training process. This is applicable to the Gamma distribution only, so let’s choose a value of 0.

**Learning parameters are set.**

***Threshold Parameters Update***

1. **Would you like to include a threshold in your model?(Y/N)**

It should be noted that the user will only be asked this and the next three questions if there is at least one “2” value present in the **obs_valid** vector of their initial variable set. Answering “Y” will set a censor threshold to be utilized on the continuous observation process during training. Thus, any time index with a numerical value of “2” within the **obs_valid** vector will be treated as a censored data point. If obs_valid is not provided by the user’s initial variable set, the function will create an **obs_valid** variable in which all values are set to “1.”

Let’s choose “Y” assuming we have at least one “2” in our **obs_valid** vector.

1. **Please enter a numerical value for your threshold.**

Trials exceeding this given value will be censored.

This number is up to the user; let’s answer a value around 1.3.

1. **Please enter 1 to process censored data with imputation, or enter 2 to process censored data with a Gaussian approximation.**

A value of 1 means imputation method will be used for modeling censored data points. A value 2 means a full likelihood will be used in estimating the state variables. For further discussion, please check reference 3.

Let’s choose a value of 1.

1. **Enter 1 to update filter mean before covariance, or enter 2 to update covariance before filter mean.**

A value of 1 indicates that the mean is updated before covariance during the EM process, and vice versa for a value of 2. For further discussion, please check reference 3.

Let’s choose a value of 1.

**Threshold parameters are set.**

1. **Enter the number of compass_em training iterations.**

This numerical answer will determine how many iterations **compass_em** will run through during training. Let’s answer “250.”

1. **Please enter the full path of where to save your new variable set, ending with a backslash (“\”):**

Here the user must enter the entire path of where to save the new variable set. The user must not forget to add a backslash at the end of the path. This step can be skipped by pressing “enter”, and the file will be instead saved in the current working directory.

Ex. G:\MATLAB\COMPASS_StateSpaceToolbox\

1. **Please enter the suffix you would like to add to the “em_set” filename:**

Here the user may enter any suffix they would like. The user must be aware that the file will be saved with the prefix “em_set” and not merely as the suffix they present alone. Let’s input the number “1” which will result in a saved mat file named em_set1.mat.

Once all questions are answered, **compass_create_model** will save an updated set of variable inputs to be passed through compass_em.

**Wrapper Function compass_run_models.m to Run Multiple Models**

**compass_run_models** will automatically pass variable input sets one by one through **compass_em** and save their corresponding outputs in the StateSpaceToolbox folder of the user’s computer. Each set of output data will be saved as a mat file, labeled model_result preceding a suffix number corresponding to the order that the file was presented (in list-form) to the function. **compass_run_models** allows users to upload as many mat variable files as they so choose, thus generating as many updated models as they would like.

**NOTE:** Input to **compass_run_models** **must** be passed as strings in list form. In other words, the input to this function must be surrounded by curly brackets, and within the curly brackets mat files must be presented as strings (thus each file must be surrounded by single apostrophes and separated by commas).

Ex. The user would like to pass three input variable sets through compass_em. These variable sets are named em_set1.mat, em_set2.mat, and em_set3.mat (this follows the naming framework of **compass_run_models**).

The user will simply pass the names of each input variable sets through **compass_run_models**, and the resulting output data will be automatically saved as model_result1.mat, model_result2.mat, and model_result3.mat. Described in one line of code below, passing the input variable sets will result in the aforementioned saved output data sets:

**compass_run_models**({‘em_set1.mat’,’em_set2.mat’,’em_set3.mat’})

NOTE: the order in which the variable sets are passed through **compass_run_models** will determine the naming suffixes of the resulting output variable files. Thus, if the aforementioned variable sets were presented in reverse order like so:

**compass_run_models**({‘em_set3.mat’,’em_set2.mat’,’em_set1.mat’})

The output of em_set3 would be named model_result1, of em_set2 would be named model_result 2, and of em_set3 would be named model_result1.

**Running Multiple Model Using compass_run_models.m**

Using **compass_create_models** we will create three models. The data files in these examples are derived from the mat file called LEARNING_1.mat which can be found in the [GitHub](https://github.com/Eden-Kramer-Lab/COMPASS) repository. This mat file includes the behavioral outcome per trial, including both reaction time and binary (correct/incorrect) decisions. The data come from an associative learning task, where through the task, a rhesus macaque learns the correct response on each trial to receive reward.

The first model in this example is mimicked from the first example of the example problems located on page 1 of the manual. It will include both continuous and discrete observation, with **In** and **Ib** each including one singular column. All non-response trials will be considered missing at random, thus their corresponding **obs_valid** vector locations will all be set equal to zero. There will be only one state variable, with **nx** set equal to one.

The second model is exactly the same as the first, however it uses continuous observation exclusively and does not include discrete observation. This change will be encountered for by the user through the use of compass_create_models.

The third model is equivalent to the first model, however it includes the use of a threshold and involves the function **compass_set_censor_threshold_proc_mode.** Thus, all non-response trials will now be considered as censored data points, and their corresponding locations in **obs_valid** will be set equal to 2.

Further instruction on how to create these three models using **compass_create_models** can be found in your COMPASS toolbox folder, in the script named “compass_example_E.m”, under the first, second, and third headers labeled %% Model #1, %% Model #2, and %% Model #3. Here, step by step directions will be given for the creation of these models using **compass_create_models**.

Once all three models are constructed and their respective input variable sets are saved, **compass_run_models** will be called and three corresponding **compass_em** output sets will be generated and saved as individual mat files. This function can be found in your COMPASS_StateSpaceToolbox folder.

**Censored Data Problem Example**

In this example, we study a state and parameter estimation problem in a dataset with censored data points. We generate the state and observation data using the following model:

$$x_{k+1}=a x_{k}+\nu_{k} v_{k}\sim N(0,s_{v}^{2})$$

$$y_{k}=x_{k}+w_{k} v_{k}\sim N(0,s_{w}^{2})$$

We define a set of threshold values – $T_{i}$– which defines the upper bound on observation. Given the threshold, the observation is defined by

$$o_{k}=\left\{ \begin{matrix} y_{k} & y_{k}<T_{i} \\ \mathrm{censored} & y_{k}\geq T_{i} \end{matrix} \right.$$

The objective is to estimate model parameters - ($a, s_{v}^{2},s_{w}^{2}$) - and state - $x_{k}$ – given $o_{k}$ and $T_{i}$.

We simulate the data using the following parameter settings:

$$a=0.99$$

$$s_{v}^{2}=0.01$$

$$s_{w}^{2}=0.055$$

and we assume the length of observation is 200 - $k=1,\ldots,200$. Below figure shows the simulated data - $x_{k},y_{k},o_{k}$. For $o_{k}$, the threshold - $T_{i}$ - is set to 0.1563, from a possible set of values ranging from -.6131 to 1.2143.

**
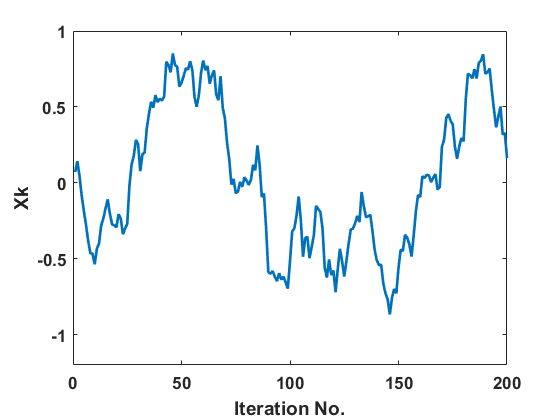
**

**Figure 8** $\boldsymbol{x}_{\boldsymbol{k}}$ state estimate

**
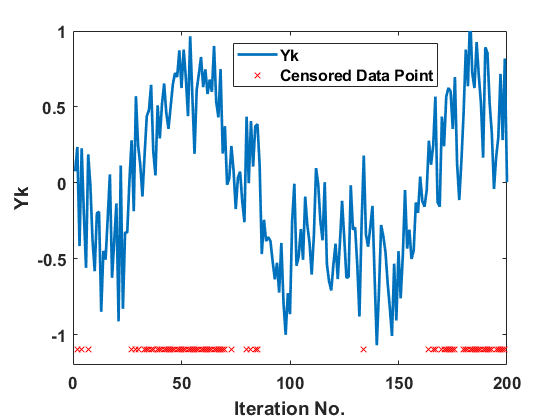
**

**
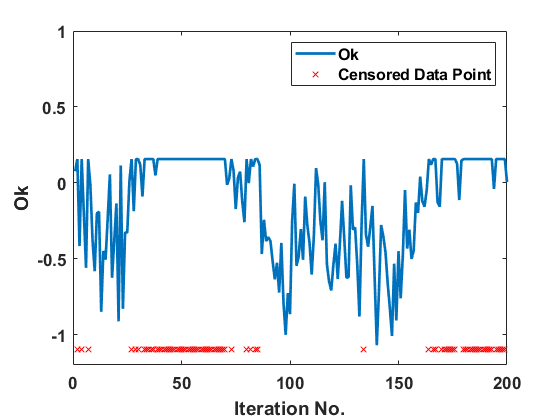
Figure 9** $\boldsymbol{y}_{\boldsymbol{k}}$ true value

**Figure 10** $\boldsymbol{o}_{\boldsymbol{k}}$ with $\boldsymbol{T}_{\boldsymbol{i}}$ = 0.1563

We use compass_create_example_F_data.m to generate the data. The Matlab code for generating this data is as follows, and the simulation result is save in the em_set100.mat file.


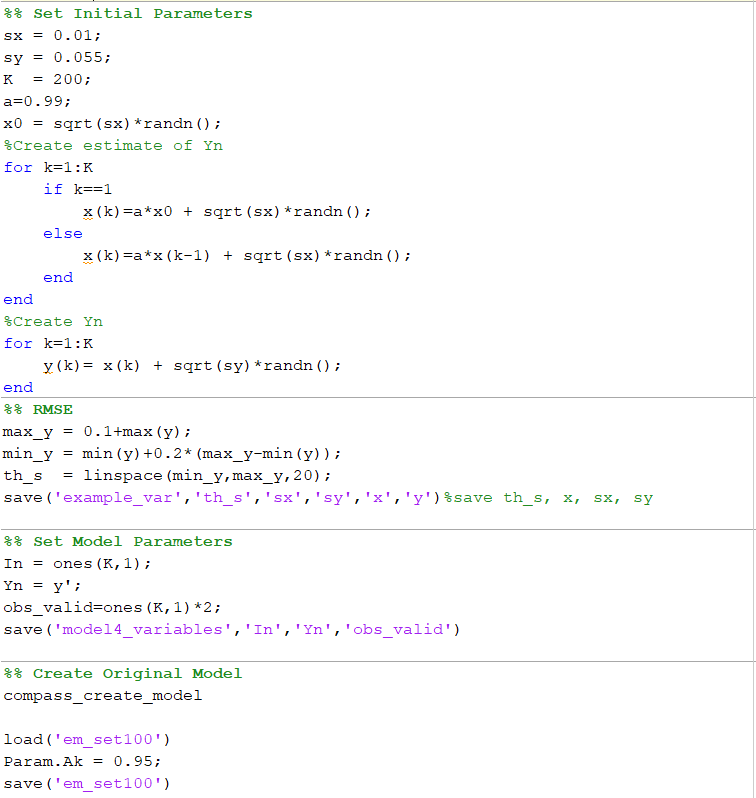


We use COMPASS to estimate the state and model parameters. COMPASS can handle the censored data problem three different ways. In the first method, the censored data are assumed to be randomly dropped – MAR – which is generally the naïve and less accurate model as no information is being passed on the censored data points. The second method is imputation, where COMPASS generates a sample per each censored data point. The last method is the full likelihood method, where COMPASS uses the full likelihood of censored data points in both state and parameter estimation. In this example, we study MAR and full-likelihood method results for a different set of thresholds. The assumption is the full likelihood method is more robust and can estimate the state and parameters with a better accuracy even in presence of many censored data points. To assess COMPASS’ and different algorithms’ performance in estimating state, we use RMSE between estimated state mean and true state generated by simulation. For parameter estimation, we visualize the deviation of parameters from their ground truth values. The hypothesis is RMSE stays reasonably the same independent of the number of censored data points in the full-likelihood method, whereas it grows in the MAR method as the number of censored data points increase. Similarly, we assume parameter estimation in the full-likelihood method is more accurate and robust in the presence of censored data points. Note that when there are no censored data points, MAR and full likelihood have the same performance.

We use the compass_create_example_F_data.m file to create the original model, and we then build more models using the **compass_run_models** function. Note that the structure of COMPASS models is the same for both MAR and full-likelihood methods; however, they need different settings to run different estimation methods. For MAR, we only need to change the **obs_valid** variable: All values exceeding the given threshold are set equal to zero. For the full likelihood method, we need two changes. First, in **obs_valid**, all values exceeding the given threshold are set equal to two. The second change is in Param. For each threshold value, we need to pass the proper value to this function, which is defined by setting Param.censor_time equal to the desired threshold value.

Within compass_example_F.m, the changes needed to account for both MAR and full-likelihood models are made. The code iterates through each threshold value. For the full-likelihood model, the code changed obs_valid elements to two if corresponding **Yn** values are greater than the threshold. For the MAR model, these corresponding elements are changed to zero. Once obs_valid is altered, for the full-likelihood model, the resulting data is saved into a new data set labeled ‘emset1’ with a suffix ranging from 1-20 (20 is the number of threshold values), pending on the order in which the data was created. For the MAR model, the resulting data is saved into a new data set labeled ‘emset2’ with the same suffix system as for the full-likelihood model.

Once 20 data sets are each saved for the full-likelihood and MAR cases (40 sets total), **compass_run_models** iterates through all 40 sets, passing these datasets through compass_em one by one. As per the functioning of **compass_run_models**, the resulting parameters from each set will be saved as “model_result” with a suffix equal to the numbered order in which the original dataset was passed through the function.

Below, Figure 9A shows the number of censored data points for different $T_{i}$. $T_{i}$ changes from -.6131 to 1.2143 with 20 linearly spaced samples; as the threshold drops the number of censored data points increases. Figures 9B-9C show RMSE plus parameter estimates using MAR and full likelihood method for all given threshold levels. Figure 9B shows RMSE as function of censored threshold for both methods. The result is in accordance with our assumption, and it suggests the full likelihood method gives a more robust estimation of the state. Figures 9C and 9D show parameter estimates using both methods for different threshold levels. Note that both methods provide an accurate estimation of the model parameters when there are no censored data points. As the number of censored data points increases, we expect the estimation of these parameters to be biased. Note that the full-likelihood method is still robust and provides an accurate estimation of model parameters even with a large number of censored data points.

| 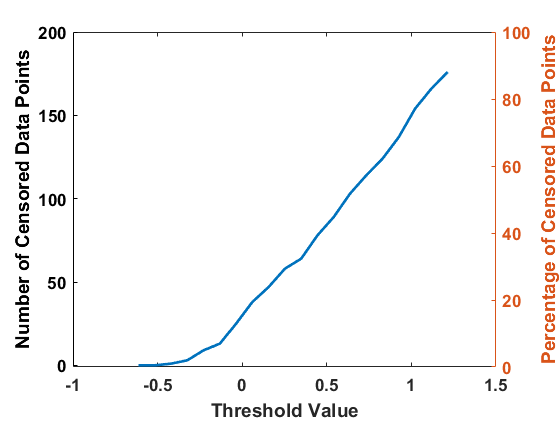 | **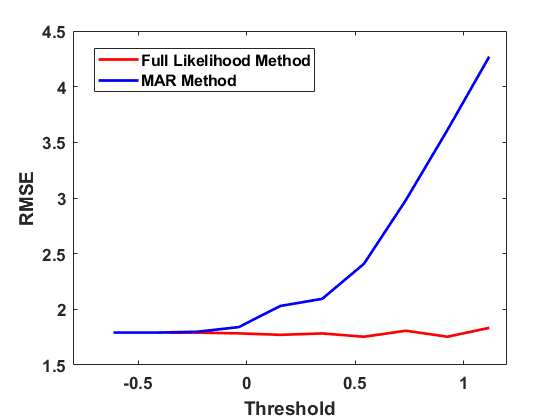** |
| --- | --- |
| **Figure 9A** Number of censored data points increases linearly as threshold value increases, exemplifying a positive correlation. Ideally, censored data points should be around 60% or else. | **Figure 9B** For full-likelihood estimate, RMSE curve remains fairly constant as threshold value increases. Unlike MAR estimate, where RMSE increases with threshold value. |
| 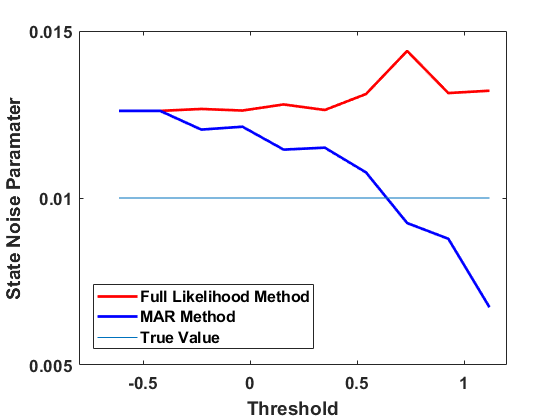 | 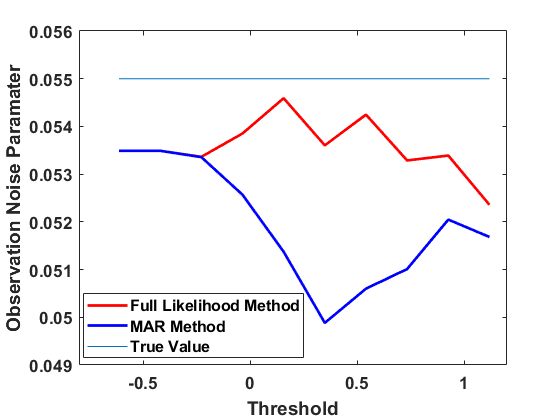 |
| **Figure 9C** For state noise parameter estimate, full-likelihood estimate remains thoroughly consistent as threshold value increases. Both full-likelihood and MAR estimates are near true value. | **Figure 9D** For observation noise parameter estimate, full-likelihood estimate undergoes slight fluctuations while remaining mostly constant. MAR estimate experiences trough and recovers. |

**Figure 9**

Besides these result, we can study other components of COMPASS and model estimations. Figure 10 shows the state estimation and ML curve for $T_{i}$ using Full likelihood and MAR models.

**Figure 10**

| 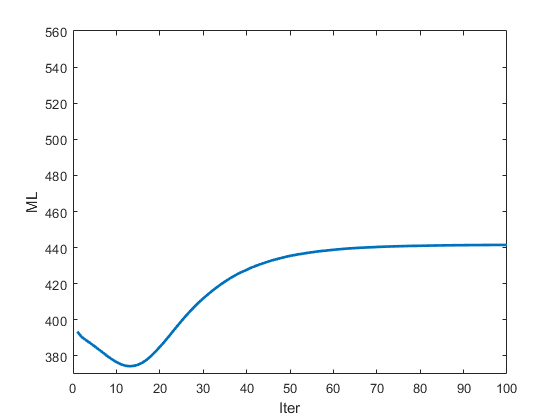 | **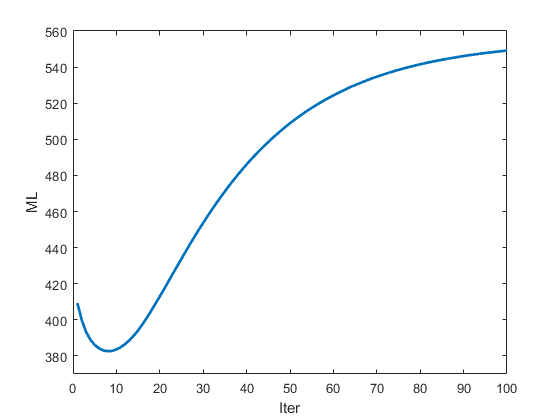** |
| --- | --- |
| **Figure 10A** Full likelihood ML curve for $T_{i}= 0.1563$. | **Figure 10B** MAR ML curve for $T_{i}= 0.1563$. |
| 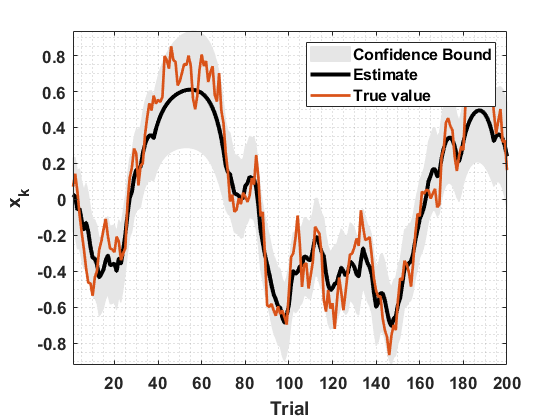 | 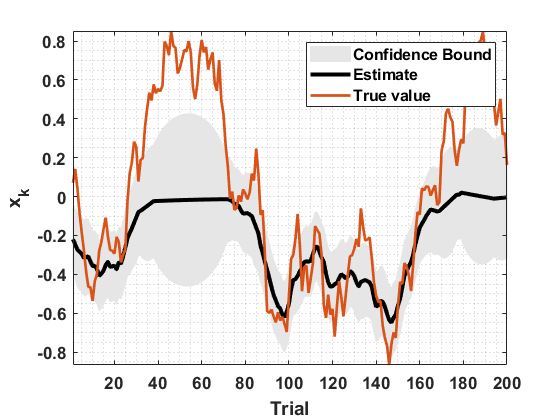 |
| **Figure 10C** Full likelihood state estimate with confidence interval and actual value. Note the similarity between all three. $T_{i}=0.1563$ | **Figure 10D** MAR state estimate with confidence interval and actual value. Note the difference between Figure 15. $T_{i}=0.1563$ |

ML curves suggest that both models are converging to a local maximum, however they might provide different predictions of the state and model parameters. For the full likelihood model, the state estimation is reasonably accurate even on the censored data points, whereas for the MAR model, we can see the bias in estimation. Note that censored data points are providing information, and the full likelihood is using this information to predict model parameters and state.

What we have shown in this example shows COMPASS’ capability in predicting the model parameters and state variables under different levels of censored data points. When there is not a censored data point, COMPASS properly estimates the model parameters and state variable. COMPASS is also capable of running different methodologies to estimate state and model parameters in presence of the censored data points and provide a robust and accurate estimation. This example provides a simple model, we can use COMPASS functions – as we described in compass_create_example_F_data.m and compass_example_F.m – to build more complex models and then use COMPASS utilities to predict model parameters.

**Manual**

Types of observed signals which can be passed to the toolbox are:

1. Normal/Log-Normal
2. Gamma
3. Bernoulli (discrete/binary choice)
4. Normal/Log-Normal + Bernoulli
5. Gamma + Bernoulli

Observed signals might include both continuous or discrete scalar values.

The toolbox includes the following functions:

1. compass_create_state_space
2. compass_set_learning_param
3. compass_em
4. compass_filtering
5. compass_sampling
6. compass_set_censor_threshold_proc_mode
7. compass_deviance
8. compass_param_covariance_info

Internal functions of this toolbox are:

1. compass_Qk
2. compass_Tk
3. compass_x_2_rp

Plot function compass_plot_bound can be used to plot state-variable confidence intervals.

**References:**

1. Yousefi, A., Paulk, A. C., Deckersbach, T., Dougherty, D. D., Eskandar, E. N., Widge, A. S., & Eden, U. T. (2015, August). Cognitive state prediction using an EM algorithm applied to gamma distributed data. In *Engineering in Medicine and Biology Society (EMBC), 2015 37th Annual International Conference of the IEEE* (pp. 7819-7824). IEEE..
2. Prerau, M. J., Smith, A. C., Eden, U. T., Kubota, Y., Yanike, M., Suzuki, W., ... & Brown, E. N. (2009). Characterizing learning by simultaneous analysis of continuous and binary measures of performance. *Journal of Neurophysiology*, *102*(5), 3060-3072.
3. Yousefi, A., Dougherty, D. D., Eskandar, E. N., Widge, A. S., & Eden, U. T. (2017). Estimating Dynamic Signals From Trial Data With Censored Values. *Computational Psychiatry*, *1*, 58-81.
4. Nelder, J. A., & Baker, R. J. (1972). *Generalized linear models*. John Wiley & Sons, Inc.
5. Venables, W.N. and Ripley, B.D., 2013. *Modern applied statistics with S-PLUS*. Springer Science & Business Media.
6. Kass, R.E., Eden, U.T. and Brown, E.N., 2014. *Analysis of neural data* (Vol. 491). New York: Springer.
7. Seltman, H.J., 2012. Experimental design and analysis. *Online at: http://www. stat. cmu. edu/, hseltman/309/Book/Book. pdf*.
8. Widge, A.S., Ellard, K.K., Paulk, A.C., Basu, I., Yousefi, A., Zorowitz, S., Gilmour, A., Afzal, A., Deckersbach, T., Cash, S.S. and Kramer, M.A., 2017. Treating refractory mental illness with closed-loop brain stimulation: Progress towards a patient-specific transdiagnostic approach. *Experimental neurology*, *287*, pp.461-472.

Written by Ali Yousefi - Jan , 2018

**compass_create_state_space**

It creates the state space model for a specific task. The function defines how each continuous and discrete part of the model is defined, and how they are linked to the state variables. This is the first function required to be called in this toolbox.

**Syntax**

Param = compass_create_state_space(nx,nUk,nIn,nIb,xM,cLink,cLinkUpdate,dLink,dLinkUpdate)

**Description**

Param = compass_create_state_space(nx, nUk,nIn,nIb,xM,cLink,cLinkUpdate,dLink,dLinkUpdate)

The function returns the parameters of the specified behavioral model defined by the function input arguments. The Param contains the model structure and necessary parameters.

**Input Arguments**

| nx | A scalar that defines dimension of state variable ($X$)  **Example:** $nx=2$, this means $X$ is a vector with dimension 2 - $X=\left[ x_{1} x_{2} \right]’.$ |
| --- | --- |
| nUk | A scalar that defines the dimension of input to the state-space model  $X\left( k+1 \right)= A X\left( k \right)+ B U\left( k \right)+W$, $U(k)$ is the input vector with “$nUk$” elements.  **Example:** $nUk=2$, for instance the $U(k)=[1 s(k)]$ has a dimension of 2. The “$1$, $s(k)$” are the components of the input to the state transition model. For example, at time $k=1$, $U(1)=[1 0]$, which means $s(1)$ is equal to 0 at time 1.  Note that $nUk$ can be set to zero when there is no $U\left( k \right)$ term in the model. |
| nIn | A scalar that defines the dimension of input passed to the continuous part of the model  $y_{c}(k)= g_{c}(I_{n}(k), X(k),P,v)$; $I_{n}(k)$ is the input vector with “$nIn$” columns and $P$ are the model free parameters.  Note that $I_{n}$ will be a matrix with size $K\times nIn$, where $K$ is the length of the process (the number of trials).  For the Normal distribution, the $g_{c}()$ function is a linear function of the $X$ and other model parameters: $y_{c}\left( k \right)={CT}_{k}X\left( k \right)+{DT}_{k}+v$ Note that ${CT}_{k}$ and ${DT}_{k}$ are functions of $I_{n}(k)$ and $P$, and variable $v$ is the observation process noise. The format of ${CT}_{k}$ and ${DT}_{k}$ is defined by the other input arguments (In, Clink, xM) to the *compass_create_state_space* function. Check xM, CLink and CLinkUpdate for more explanation.  For the Gamma distribution, you might check the function definition described in **Reference 1**.  **Example:** $nIn=5,$ for instance $I_{n}(k)=[I_{b}\left( k \right) I_{c}\left( k \right) I_{h}\left( k \right) I_{i2c}(k) I_{c2i}(k)]$ has a dimension of 5. The “$I_{b}$, $I_{c}$, $I_{h}$, $I_{i2c}$, and $I_{c2i}$” can be indicator functions for the behavioral tasks, i.e. set to 0 or 1 depending on the properties of each trial (Reference 1, Reference 8).  For example, at time $k=1$, $I_{n}(1)=[1 0 1 1 0]$. |
| nIb | A scalar that defines the dimension of the input to the discrete – binary – part of the model  $y_{d}(k)= g_{b}(I_{b}(k), X(k),Q)$. $I_{b}(k)$ is the input vector with “$nIb$” elements, and the $g$ function is defined by a sigmoid function: $P(y_{d}\left( k \right)==1)={exp \left( {ET}_{k} X\left( k \right)+{FT}_{k} \right)}/\left( 1+exp \left( {ET}_{k} X\left( k \right)+{FT}_{k} \right) \right)$ Note that ${ET}_{k}$ and ${FT}_{k}$ are functions of $I_{b}(k)$ and $Q$. The format of ${ET}_{k}$ and ${FT}_{k}$ is defined by the other input arguments (Ib, DLink, xM) to the *compass_create_state_space* function. Check xM, DLink and DLinkUpdate for more explanation.  **Example:** $nIb=5$, for instance $I_{d}(k)=[I_{b}\left( k \right) I_{c}\left( k \right) I_{h}\left( k \right) I_{i2c}(k) I_{c2i}(k)]$ has dimension of 5. The “$I_{b}$, $I_{c}$, $I_{h}$, $I_{i2c}$, and $I_{c2i}$” are indicator functions for the behavioral task which are the input to the discrete part of the behavioral model.  For example, at time $k=1$, $I_{d}(1)=[1 0 1 1 0]$. |
| xM | A transform matrix which determines how the state variables are used in both $y_{c}(k)$ and $y_{d}(k)$ observation processes. The default transform matrix is an identity $nx\times nx$ matrix, but it can be a rectangular matrix with a larger number of rows than its columns.  Elements of the $xM$ are 0 and 1, and the sum of each row is equal to 1.  Note that rather than using $X(k)$ in the continuous or discrete process, we use $xM\times X(k)$. Using the $xM$, we have more flexibility in defining the observation processes used in the model. When $xM$ is an identity matrix, each state variable can be linked to one of the input elements, whereas using a rectangular $xM$, we will be able to define behavioral models for which each state variable can be linked to more than one input element. For example, we might have the following behavioral model: $y_{c}\left( k \right)=(I_{c}\left( k \right)+\beta_{0}*I_{h}\left( k \right))*x\left( k \right)+I_{h}\left( k \right)+v$ here, the state variable is linked to two input elements. To build this model, we need to set $xM=\left[ \begin{matrix} 1 \\ 1 \end{matrix} \right]$; thus, the state variable – $nx=1$ here – is linked to both $I_{c}\left( \cdot\right)$ and $I_{h}\left( \cdot\right)$. Note that $I_{c}\left( \cdot\right)$ and $I_{h}\left( \cdot\right)$ will be columns of $In$ input passed to the model.  **Default:** an identity $nx\times nx$ matrix  **Example:** $xM\boldsymbol{=}\left[ \begin{matrix} 1 & 0 \\ 0 & 1 \\ 0 & 1 \end{matrix} \right]$, the state variable utilized in the behavioral model is treated as a three-component state variable, where the second and third components are the same. Note that the $nx$ must be 2 here. |
| cLink | A matrix of size $1\times tx$, where $tx$ is the number of $xM$ rows.  In this toolbox, we assume that there are $tx$ state variables rather than $nx$, and $cLink$ defines which of $I_{n}$'s columns is linked to each state variable – check $In$ in compass_em function. Elements of the matrix can have values between $1$ to $nIn$. $I_{n}$ is the input to the continuous observation process with size of $K\times nIn$, where $K$ is the length of process or number of trials.  **Example:** $cLink=\left[ \begin{matrix} 1 & 3 & 4 \end{matrix} \right]$, this means there are 3 state variables, and the first state variable is linked to the first column of $I_{n}$ and so on. This implies that, $nIn$ should be at least 4. The $\mathrm{cLink}$ suggests that the dynamic part of the continuous process will be defined by: $I_{n,1}\left( k \right)x_{1}\left( k \right)\beta_{1}+I_{n,3}\left( k \right)x_{2}\left( k \right)\beta_{2}+I_{n,4}(k)x_{3}(k)\beta_{3}$ here, ($\beta_{1},\beta_{2},\beta_{3}$) are model parameters, and ${CT}_{k}=[I_{n,1}\left( k \right)*\beta_{1} I_{n,3}\left( k \right)*\beta_{2} I_{n,4}\left( k \right)*\beta_{3}]$.  We will explain in the cLinkUpdate definition, which defines which of the parameters are fixed or should be estimated using the learning step. |
| cLinkUpdate | A matrix of size $1\times tx$, similar to cLink. Elements of the matrix are 0 and 1, and it determines whether the corresponding parameter - $\beta_{i}$ – will be updated or not, during the compass_em. A value of 1 means $\beta_{i}$ will be updated, and value of 0 means $\beta_{i}$ is fixed. The default value for the fixed parameters is 1. Check the cLink argument description. Note that cLink defines the model structure and then using cLinkUpdate function we determine which of the model parameters needs to be estimated in the learning step.  **Example:** $cLinkUpdate=\left[ \begin{matrix} 1 & 0 & 0 \end{matrix} \right]$, this means only the first variable will be updated using EM algorithm. Note that this refers to the parameter linked to the first state variable, not to the first column of $I_{n}(k)$. Check cLink example, this means the continuous part of the continuous process is defined by: $I_{n,1}\left( k \right)x_{1}\left( k \right)\beta_{1}+I_{n,3}\left( k \right)x_{2}\left( k \right)+I_{n,4}(k)x_{3}(k)$ In the other words, it means $\beta_{2}=\beta_{3}=1$ – fixed values, it can be any other values as well – and $\beta_{1}$ will be adjusted through the EM algorithm. |
| dLink | dLink is equivalent to cLink for discrete – or binary - process. Check the cLink for further information.  Note that we could set these parameters with dummy or null values if there are no discrete processes. The setting won’t be used when we the learning – EM – function later. |
| dLinkUpdate | dLinkUpdate is equivalent to cLinkUpdate for discrete process. Check the cLinkUpdate for further information.  Note that we could set these parameters with dummy or null values if there are no discrete processes. The setting won’t be used when we the learning – EM – function later. |

**Output Arguments**

| Param | A structure array that defines the state-space and behavioral model structure and paramaters. Note that most \ elements are filled with their default values and they need to be set either manually or by calling compass_em function.  The Param structure fields are:   - **nx** – Check the input argument - **cLinkMap** – It is equal to cLink - **dLinkMap** – It is equal to dLink - **nIn** – Check the input argument, number of In elements. - **nIb** – Check the input argument, number of Ib elements. - **Ak** – The state-transition model is defined by: $X\left( k+1 \right)= A X\left( k \right)+ B U\left( k \right)+W$  The Ak defines the $A$ variable of the state-transition model.   - **Bk** – the state-transition model is defined by: $X\left( k+1 \right)= A X\left( k \right)+ BU\left( k \right)+W$  The Bk defines the $B$ variable of the state-transition model.   - **Wk** – the state-transition model is defined by: $X\left( k+1 \right)= A X\left( k \right)+ B U\left( k \right)+W$  The W defines the noise covariance term, and it is assumed to be stationary with a zero-mean.   - **X0** – The state-variable estimate at time 0. It is assumed that state variable at time 0 has a Normal distribution with mean X0 and variance W0. - **W0** – The covariance matrix of the state-variable estimate at time 0. - **Ck** – Model parameters for the ${CT}_{k}$ section of the continuous observation process: $y_{c}\left( k \right)= {CT}_{k} X\left( k \right)+{DT}_{k}+v$  Note that ${CT}_{k}$ at time $k$ is defined by: $\left( C_{k}*In(k,cLink) \right)\times xM$- $*$ is an element-by-element multiplication, and $\times$ is a dot product.   - **Dk** – Model parameters for the ${DT}_{k}$ of the continuous observation process: $y_{c}\left( k \right)= {CT}_{k}X\left( k \right)+{DT}_{k}+v$  Note that ${DT}_{k}$ at time $k$ is defined by: $\left( D_{k}*DMk \right)\times In(k,:)'$. $DMk$ elements are either 0 or 1, and it defines which columns of the $In$ contribute in the stationary part of the observation process. $DMk$ is equal to zero for the columns identified in cLink; thus, the stationary part of the observation process is defined by a linear combination of input columns not being linked to state variables.  Note the $DMk$ is an internal parameter of the toolbox, which is derived from cConstantUpdate.  Note that, if we want to have a term similar to: $I_{c}\left( k \right)*x\left( k \right)+\beta_{0}*I_{c}\left( k \right)$ in the observation process, we should replicate $I_{c}\left( k \right)$ twice in the $In$ definition.   - **cConstantUpdate** – It is a vector of length $nIn$, and it determines which elements of $D_{k}$ will be set to zero. Elements of the cConstantUpdate are either 0 or 1, where elements with value 1 determines columns of $In$ used in the stationary part of the observation process. Note that $DMk$ is derived using the cConstantUpdate. The cConstantUpdate is 1 for the input columns not linked to the state variables and it is set 0 for the input columns lined to the state variables. - **Vk** – The variance for the continuous observation noise, $v$. This will be dispersion term for the Gamma distribution. Check **Reference 1** for further information. - **S –** It defines a constant shift in the observation signal and it is used for Gamma distribution. Check **Reference 1** for further information. - **Ek** – It is equivalent to Ck, and it is used for the discrete part of the state-space model. - **Fk** – It is equivalent to Ek, and it is used for the discrete of the state-space model. - **dConstantUpdate** – It is equivalent to cConstantUpdate, and it is used for the discrete part of the model. - **xM** – check the input argument |
| --- | --- |

**Example**

Param = compass_create_state_space(3,1,4,4,eye(3,3),[1 3 4],[1 1 1],[1 3 4],[0 0 0]);

Order of the input parameters to create the state space model:

1. The state-variable dimension is set to be 3.

$$X\left( k \right)=\left[ x_{1}\left( k \right) x_{2}\left( k \right) x_{3}\left( k \right) \right]'$$

2. The input to the state-space transition model is a scalar, length 1.
3. Given items in 1 and 2, the state transition process is:

$$X\left( k+1 \right)=AX\left( k \right)+bU\left( k \right)+W$$

4. The input to both the continuous and discrete model of the behavior are vectors with 4 columns and K rows, where K is the number of trials
5. The xM is an identity matrix, thus we have a state vector with 3 elements.
6. The cLink is [1 3 4], which means the first state variable is linked to the 1^st^ element of $I_{n}$, the second state variable is linked to 3^rd^ element of $I_{n}$, and the third state variable is linked to the 4^th^ element of $I_{n}.$
7. The cLinkUpdate is [1 1 1], which means all three $C$ parameters will be updated. Check cLinkUpdate.
8. Given items 6 and 7, the observation process for a normal distribution is defined by:

$$y_{c}\left( k \right)=c_{1}In\left( k,1 \right)x_{1}\left( k \right)+c_{2}In\left( k,3 \right)x_{2}\left( k \right)+c_{3}In\left( k,4 \right)x_{3}\left( k \right)+d_{1}In\left( k,2 \right)+w$$

9. The dLink is [1 3 4], which share a similar structure of the continuous model of the behavior.
10. The dLinkUpdate is [0 0 0], which means none of the $E$ parameters will be updated. Check dLinkUpdate.
11. Item 10 is very important to avoid mis-specified models of the observed signals.
12. Given items 9 and 10, the observation process for the discrete process is defined by:

$$\mathrm{Logit}\left( P(y_{b}\left( k \right)==1) \right)=Ib\left( k,1 \right)x_{1}\left( k \right)+Ib\left( k,3 \right)x_{2}\left( k \right)+Ib\left( k,4 \right)x_{3}\left( k \right)+f_{1}In\left( k,2 \right)$$

Note, Ek elements are assumed to be equal to 1.

1. Check items 8 and 11 to find the relationship between cLink, cLinkUpdate, dLink, and dLinkUpdate with the process model. Note that cLinkUpdate and dLinkUpdate must be carefully chosen, otherwise we might have a non-identifiable model.

**Note**

The compass_create_state_space sets the Ak, Bk, Wk, W0, Ck, Dk, Vk, S, Ek and Fk parameters with predefined values. Values of these parameters can be changed either inside this function or after calling this function; the other output arguments mainly define the model structure and it shouldn’t be changed manually.

We will see later that some of these parameters will be updated, while some are fixed. These parameters can be changed inside the compass_create_state_space function or even manually. Specifically, the cConstantUpdate and dConstantUpdate are defined by a specific assumption of how the input to state-space model will be linked to constant parameters or state variables, which is part of the compass_create_state_space function. We can change the structure of the behavioral model manually by changing elements of Param variables.

**compass_set_learning_param**

It sets learning parameters used in the EM algorithm, i.e. which parameters are considered fixed vs. updated based on data. Note this function can be only called after compass_create_state_space function. Using this function, we determine how the parameters of the models will be updated later using compass_em function. Note that compass_create_state_space defines a proper structure of the model, while the compass_set_learning_param sets how parameters of the state-space model will be trained using the Expectation-Maximization (EM) algorithm.

**Syntax**

Param = compass_set_learning_param(Param,Iter,UpdateStateParam,UpdateStateNoise,UpdateStateX0,UpdateCModelParam,

UpdateCModelNoise, UpdateDModelParam,DiagonalA,UpdateMode,UpdateCModelShift)

**Description**

Param = compass_set_learning_param(Param,Iter,UpdateStateParam,UpdateStateNoise,UpdateStateX0,UpdateCModelParam,

UpdateCModelNoise, UpdateDModelParam,DiagonalA,UpdateMode,UpdateCModelShift)

This function sets how the model parameters will be adjusted using the EM learning procedure run by compass_em.

**Input Arguments**

| Param | A structure array consisting both the structure and parameters of the state-space model parameters along with EM learning procedure.  Check Param in compass_create_state_space for a more complete description. |
| --- | --- |
| Iter | Number of EM training iterations.  Note that the value of *Iter* can be determined by inspecting the ML curve produced by compass_em. Afer visualizing the ML curve, we can reset the *Iter* to make sure that ML converges to a local maximum.  **Example:** $Iter=100$. The EM training iteration number is 100. |
| UpdateStateParam | It can be either 0 or 1.  A value of 1 means that the state-transition model parameters, elements of A and B matrices, will be updated. A value of 0 means that these parameters are not updated in the EM training process.  State transition process model is defined by: $X\left( k+1 \right)= A X\left( k \right)+ B U\left( k \right)+W$ **Example:** $UpdateStateParam=1$. The state-transition process A and B matrixes will be updated in the EM learning process.  Note that we either update both $A$ and $B$ or neither.  Training only $A$ or $B$ would require a change in the compass_em code. |
| UpdateStateNoise | It can be either 0 or 1.  Value of 1 means that the state-transition model covariance matrix will be updated. It is assumed that W, a covariance matrix, is diagonal. Value 0 means that W won’t be updated in the training process.  State transition process model is defined by: $X\left( k+1 \right)= A *X\left( k \right)+ B* U\left( k \right)+W$ **Example:** $UpdateStateNoise=1$. The state-transition process parameters are trained in the EM learning process. |
| UpdateStateX0 | It can be either 0 or 1.  Value of 1 means that the state variable parameters at time 0 – mean and covariance matrix - will be updated. Value 0 means that the initial parameters of the state variable at time 0 won’t be updated in the EM training process. The initial value of X0 are set inside compass_create_state_space. If UpdateStateX0 is set to 0, the user should manually initialize the state by accessing Param.X0 and Param.W0.  State transition process model is defined by: $X\left( k+1 \right)= A *X\left( k \right)+ B* U\left( k \right)+W$ $X\left( 0 \right)\sim N(X_{0},W_{0})$ **Example:** $UpdateStateX0=1$. It means $X_{0}$ and $W_{0}$ – mean and covariance matrix - of the initial value of the state-variable is estimated in compass_em function. |
| UpdateCModelParam | It can be either 0 or 1.  Value of 1 means parameters of the continuous observation process will be updated in the EM training process. The parameters are a sub-set of parameters in the $C$ set to be trained and all the parameters in $D$ vectors. Read the cLinkUpdate in compass_create_state_space function to find which subset – or index - of the $C$ parameters will be updated.  Value of 0 means parameters of the continuous observation process won’t be updated in the EM training process.  **Example:** $UpdateCModelParam=1$. It means parameters of the continuous observation process – elements of $C$ and $D$ vectors – will be updated in the EM training process. |
| UpdateCModelNoise | It can be 0 or 1.  Value of 1 means the observation process noise-term will be updated through the EM training process, compass_em. For the continuous observation process with a normal or log-normal distribution, the noise term – $v$ – represents additive Normal white noise. For the observation process with a Gamma distributed signal, the $v$ term corresponds to the dispersion term. For further information about the canonical form of the Gamma distribution and dispersion term definition, please see **Reference 1**.  For the Normal observation process, we have: $y_{c}\left( k \right)={CT}_{k}X\left( k \right)+{DT}_{k}+v$ $v\sim N(0,\sigma_{v}^{2})$ here, $v$ is a white noise with a Normal distribution with a variance of $\sigma_{v}^{2}$.  **Example:** $UpdateCModelNoise=1$. It means the noise-term of the observation process is updated through the EM training process. |
| UpdateDModelParam | It can be 0 or 1.  It is equivalent to the UpdateCModelParam , but applies to a discrete observation process. Check the UpdateDModelParam for further explanations.  Note that for the discrete process, there is no noise-term to be updated. |
| DiagonalA | It can be 0 or 1. Default is 1.  Value 1 means the $A$ matrix in the state-transition process model will be a diagonal matrix. The diagonal assumption is the favorable choice for EM learning. Note that state variables will be dependent on each other through the observation process even if $A$ is a diagonal. Also note that the posterior estimates of the state variables are not necessarily independent, because they are updated through the observation model.  Value 0 means the $A$ matrix is a full matrix. For the EM algorithm, this might lead to a less accurate or even instable estimation of both parameters and state-variables.  Note that whether $A$ is updated from data during the EM process will be determined by UpdateStateParam. The state transition process can be either an AR(1) model or a random walk. For many applications, we will want to set the state-transition process as a random-walk type process, where we set $A$ to be an identity matrix and exclude it from the training process. This does not mean that the state is fixed – the state will still change trial-to-trial based on the posterior updates (which in turn depend on the observations). It means that the state only changes based on the observations, not based on its own internal dynamics. Random-walk state processes are useful whenever we are interested in understanding how a cognitive process evolves through time.  We will have an AR(1) state model whenever *A* is something other than an identity matrix. This models a state that naturally settles over time to a default value. This might be appropriate if the state should naturally decay to 0 over time, such as a forgetting process or the decaying effect of something like a reward/punishment. It will often be helpful to then define an input term to the state process, and this input term will be a major driver of state change.  **Example:** $DigonalA =1$ – default value. It means the $A$ matrix is a diagonal matrix. |
| UpdateMode | It can be 1 or 2. Default is 2.  It determines which update method is used in the Filter mean and covariance update. Note that when we use a Gaussian approximation method, we generally might have different update rules for the mean and co-variance of the state variables. We are using two different update rules:   1. First update Mean, and then update Covariance - **UpdateMode 1** 2. First update Covariance, and then update Mean – **UpdateMode 2**   Picking the right update rule depends on the observation process and its characteristics, specifically its variability and noise. In practice, the good starting point is **UpdateMode 2.** To identify the better update rule, we can check both the estimation and ML result using both update rules, then select the one which gives a higher ML or a more stable estimation result.  For a further detail about which of these approximations might give a better result, you can check **Reference 2**. **Reference 2** describes the Gaussian approximation for the point process model, which can be extended for other distributions including Gamma, Bernouli, and or mixture of these distributions.  **Example:** $UpdateMode =2$ – default value. It means the Filter estimate of the state-variable is based on the second method used in the posterior Gaussian approximation. |
| UpdateCModelShift | It can be 0 or 1.  It determines whether the estimation of a positive shift – or bias - will be part of the EM training process or not. For the Gamma distribution, we define a positive shift and we can tune this parameter using the EM algorithm. A value of 1 means that the shift parameter will be updated in the EM training process.  A value of 0 means that this shift parameter is pre-set and it is not updated in the EM training process, compass_em function.  Note that this is a specific parameter of the Gamma distribution, and it is not used in the observation process with Normal or Log-Normal distributions.  For a further information about the Gamma distribution, please check **Reference 1**.  **Example:** $pdateCModelShift =1$ . The time shift parameter will be updated if the continuous observation process is set to be Gamma. |

**Output Arguments**

| Param | An updated structure array consisting of both the state-space model structure/parameters and EM learning parameters.  The new fields of the Param structure are:   - Iter – check the input argument - UpdateStateParam – check the input argument - UpdateStateNoise – check the input argument - UpdateStateX0 – check the input argument - UpdateCModelParam – check the input argument - UpdateCModelNoise – check the input argument - UpdateDModelParam – check the input argument - DigonalA – check the input argument - UpdateMode - check the input argument - UpdateCModelShift - check the input argument   Note that Param will also include the state-space model parameters defined by compass_create_state_space as well. |
| --- | --- |

**Example**

% create state space model

Param = compass_create_state_space(3,1,4,4,eye(3,3),[1 3 4],[1 1 1],[1 3 4],[0 0 0]);

% set the learning parameters

Iter = 300;

Param = compass_set_learning_param(Param,Iter,0,1,1,1,1,1,1,2,1);

In this example, we set Number of EM training iterations to 300. Given the input terms to the compass_set_learning_param, the state parameters are fixed and not updated in the EM step. Similarly, we can define whether other elements of the model will be updated or not by corresponding values being passed to compass_set_learning_param.

**Note**

In compass_create_state_space, we determine which part of the proposed dynamical model will have free parameters. Using the compass_set_learning_param function, we determine if these free parameters will be updated or not.

For the state-transition model, we can:

1. Set whether A will be diagonal or full matrix
2. Set whether elements of A and B matrices will be updated or not – Both *A* and *B* will determine the state-transition dynamics, and for this reason, we either update both *A* and *B* or none of them. Separate updating of only *A* or *B* will be available in a future version of COMPASS.
3. Set whether the noise covariance matrix – which is assumed to be diagonal – will be updated or not.
4. Set whether X0 variable distribution will be updated or not.

For the continuous behavioral model, we can:

1. Set whether model parameters will be updated or not. This is a selected subset of the *C* and all D elements.
2. Set whether observation noise will be updated or not.
3. For the Gamma distribution, we can determine whether the shift term will be updated or not.

For the discrete behavioral model, we can set whether model parameters will be updated or not.

**compass_set_censor_threshold_proc_mode**

It sets censor threshold for the continuous observation process and how it will be processed in the training step. The censoring threshold will be added to the Param structure. We assume that on the censored trials, the continuous observation process exceeds the censoring threshold and that both the continuous and discrete observations are unobserved. Check obs_valid argument in the **compass_em** for further definition of the censored or missed trials.

**Syntax**

Param = compass_set_censor_threshold_proc_mode(Param,censor_thr, censor_mode,update_mode)

**Description**

Param = compass_set_censor_threshold_proc_mode (Param,censor_thr,censor_mode,update_model)

This function gets the Param structure plus the censored value, censor_thr, and it updates the Param structure. The censor_time in the Param keeps the value of censoring threshold. The censor_mode and update_model define how the censored points will be processed in the training step. At present, imputation of censored points is only available for a univariate continuous observation; vector-valued continuous observations cannot be imputed through this function.

**Input Arguments**

| Param | A structure array consisting both the state-space and observation process model parameters along EM learning parameters.  Check Param in compass_create_state_space and compass_set_learning_param for a more complete description. |
| --- | --- |
| censor_thr | It define the continuous observation process censor time.  The censor_time variable of the Param will be equal to censor_thr.  **Example:** $censor\_thr=1$. It means that trials are censored whenever they have a continuous observation process value larger than 1. |
| censor_mode | It defines which processing mode is being for censored data points. There are two modes, 1 and 2.  The censor_mode equal to 1 uses imputation (single imputation) to process censored data point. In this mode, the compass_em process will treat the imputed points as though they were actually observed. In other words, missing observations will not affect the confidence bounds of the state estimate.  The censor_mode equal to 2 uses a Gaussian approximation, to approximate the full likelihood of censored data. This is the method described in Reference 3. As in that paper, the state estimate will have wider confidence bounds on missing data, because the uncertainty is propagated through.  **Example:** $censor\_mode=2$. It means the censored trials are processed and the process values imputed using a full-likelihood method. |
| update_mode | It defines how the fast Gaussian solution will be run on the censored data points to impute them under censor_mode = 2. There are two modes, 1 and 2  This setting is similar to UpdateMode defined for compass_set_learning_param and defines how the filter solution is called over the censored data points.  When censor_mode is set to 1, we impute data for the censored trials and the data point is treated as an observed point. Thus, this setting is only effective when we use the Gaussian approximation mode (censor_mode =2) for processing censored data points.  **Example:** $update\_mode=1$. It means that missing censored data will be imputed using a Gaussian filtering approximation, where first the mean and then the covariance is updated during the EM process. |

**Output Arguments**

| Param | An updated structure array of Param consisting of model structure, model parameters and EM learning parameters. The new field of the Param structure is: censor_time - check the input argument  The following arguments are being added to the Param.  Param.censor_time: it keeps the censoring threshold  Param.censor_mode: it keeps the censor_mode argument  Param.censor_update_mode: it keeps update_mode argument |
| --- | --- |

**Example**

censor_time = 1;

censor_mode= 2;

update_mode= 1;

Param= **compass_set_censor_threshold_proc_mode** (Param,censor_time, censor_mode, update_mode);

This means that the censored trials will have values larger than censor_time which is 1 here. The full likelihood methodology will be called on the censored data points, and the update_mode is type 1.

**compass_em**

This function runs the EM algorithm to adjust model parameters and estimate the state variables. This is the ***core*** function of the toolbox. It takes the model structure, input and observation data and it returns updated model parameters and state estimates. It also returns the Maximum Likelihood (ML) estimate which shows progression of the ML through multiple iterations.

**Syntax**

[rXSmt,rSSmt,Param,rXPos,rSPos,ML,EYn,EYb,rYn,rYb]=compass_em(DISTR,Uk,In,Ib,Yn,Yb,Param,obs_valid)

**Description**

[rXSmt,rSSmt,Param,rXPos,rSPos,ML,EYn,EYb,rYn,rYb]= compass_em(DISTR,Uk,In,Ib,Yn,Yb,Param,obs_valid)

The function gets input arrays (Uk,In,Ib), observation signals (Yn,Yb, obs_valid), model parameters (Param), and type of observation processes (DISTR), and returns an estimate of state variables (smoother - rXSmt,rSSmt -, plus filter - rXPos,rSPos) along with the likelihood value on each iteration (ML), expected observed signals (EYn,EYb), and updated observed signals along with imputed data for censored trials (rYn,rYb).

The filter estimate uses past and current observations to predict the current state. It is causal – the state at each timepoint is only influenced by prior observations.

The smoother estimate uses all the observations – past and future - to predict the current state. Smoother output is non-causal, but may give a more accurate estimate of the underlying process and is usually more suitable for off-line analysis.

**Input Arguments**

| DISTR | A 2 element vector which defines the type of the observed signals and their corresponding distributions.  **DISTR=[1 0]** means there is only continuous observation signal and the proper distribution for the signal is Normal. Note that the input argument Yn carries the continuous signal.  **DISTR=[2 0]** means there is only continuous observation signal and the proper distribution of the signal is Gamma. Note that the input argument Yn carries the continuous signal.  **DISTR=[0 1]** means there is only discrete observation – a Bernoulli observation process.  Note that the input argument Yb carries the continuous signal.  **DISTR=[1 1]** means there are both continuous and discrete observation signals and the proper distribution for the signal is a mixture of Normal and Bernoulli distributions. Note that the input arguments Yn and Yb carry the mixed signals.  **DISTR=[2 1]** means there are both continuous and discrete observation signals and the proper distribution for the signal is a mixture of Gamma and Bernoulli distributions. Note that the input arguments Yn and Yb carry the mixed signals.  **Example:** $DISTR =[1 0]$, it means the observed signal includes only continuous observation. Note that we might define the state-space model with both continuous and discrete observations; the EM algorithm might process either continuous or discrete parts of the process given the $DISTR$ elements. |
| --- | --- |
| Uk | A matrix of size $K\times nUk$ which defines the input to the state-transition model – $K$ is the length of observation, and $nUk$ is number of elements per each time index. The state-transition process is defined by: $X\left( k+1 \right)= A X\left( k \right)+ B U\left( k \right)+W$ **Example:** $Uk=ones(100,1)$, it mean that input is a fixed value of 1 at each time index. Note that it is assumed that $K$ is equal to 100, which is the length of the observation. |
| In | A matrix of size $K\times nIn$ which defines the input to the continuous observation process. For further information about how columns of $In$ is utilized in the continuous observation process, check the compass_create_state_space function.  **Example:** $In=randn(100,5)$; it means a random input with 5 elements at each time index is passed to the continuous observation process. Note that it is assumed that $K$ is equal to 100, which is the length of the observation.  Practically, the $In$ elements could be indicator functions of the task factors or even continuous values, including history terms.  Note that $Uk$, $In$, and $Ib$ share the same number of rows. |
| Ib | A matrix of size $K\times nIb$ which defines the input to the discrete observation process. It is equivalent to $In$ of the continuous part.  **Example:** $Ib=randn(100,5)$; it means a random input with 5 elements at each time index is passed to the discrete observation process. Note that it is assumed that $K$ is equal to 100, which is the length of the observation.  Practically, the $Ib$ elements could be indicator functions of the task factors or even continuous values, including history terms.  Note that $Uk$, $In$, and $Ib$ share the same number of rows. |
| Yn | A vector of size $K\times1$ which carries the continuous observation signal - $K$ is the length of observation process.  Values of Yn can real values or NaN. Note that for a NaN observation signal, the corresponding obs_valid must be either 0 or 2. Check obs_valid for further information.  Note that for an observation process with a Normal distribution, $DISTR=[1 X]$, the Yn elements may be any real values. For the observation process with a Gamma distribution, $DISTR=[2 X]$, the Yn elements must be positive real-valued.  **Example:** $Yn=rand(100,1)$; it means a positive random input with 100 rows.  Note that $Uk$, $In$, $Ib$, and $Y_{n}$ share the same number of rows. |
| Yb | A vector of size $K\times1$ which carries the discrete observation signal - $K$ is the length of observation process.  Values of Yb are 0, 1, or NaN. Note that for a NaN observation signal, the corresponding obs_valid must be either 0 or 2. Check obs_valid for further information.  Value of 0 means an incorrect response, and a value of 1 means a correct response.  **Example:** $Yb=randi(2,[100 1])-1$, which means a vector with random values of 0 or 1 at each row.  Note that $Uk$, $In$, $Ib$, $Yn$, and $Yb$ share the same number of rows. |
| Param | A structure array consisting of both the state-space model structure plus EM learning parameters.  Check Param in compass_create_state_space and compass_set_learning_param for a more complete description. |
| obs_valid | A vector of length K with elements of 0, 1, and 2.  An element with value 1 means the observed signal is valid.  An element with value 0 means the observation is missing.  An element with value 2 means the observation is censored. The censoring threshold is defined by the Param.censor_time.  Note that a NaN value in either Yn or Yb must be accompanied by a value 0 or 2 in obs_valid, otherwise the EM algorithm fails to have a correct estimate of the model parameters and state variables.  Note that obs_valid determines how the EM algorithm treats each row of the observed signal in running either E- or M-step of the EM process. Thus, setting correct values of obs_valid has a significant importance in running the model correctly.  **Example:** $obs\_valid= ones(100,1)$, it means all elements of the observed signals are filled with valid values.  Note that $Uk$, $In$, $Ib$, $Yn$, $Yb$, and $obs\_valid$ share the same number of rows. |

**Output Arguments**

| rXSmt | The mean value of the state variable smoother result.  A structure array of length K, where K is the number of observation samples. Each element of the $rXSmt$ is a vector of length $nx$. |
| --- | --- |
| rSSmt | The covariance matrix of the state variable smoothing result.  A structure array of length K, where the K is number of observation samples.  Each element of the $rSSmt$ is a matrix of size $nx\times nx$. |
| Param | Updated Param structure array which returns EM learning result.  Note that Param keeps all the model parameters. |
| rXPos | The mean value of the state variable filter result.  A structure array of length K, where K is the number of observation samples.  Each element of the $rXPos$ is a vector of length $nx$. |
| rSPos | The covariance matrix of the state variable filter result.  A structure array of length K, where the K is the number of observation samples.  Each element of the $rSPos$ is a matrix of size $nx\times nx$. |
| ML | Maximum likelihood value of the EM algorithm at each iteration.  The ML is a vector of the length Iter. Iter is the number of EM iterations, and it is passed through Param to compass_em.  Practically, the ML might be an increasing function of the iteration. In the other words, it is expected to grow as the number of iteration increases.  ML growth rate or reaching a plateau might be an indication of enough number of EM iteration. The ML value can be used as the stop criterion of the algorithm; a future version of compass_em will contain this non-deterministic stopping . Note that the optimal number of $Iter$ which is passed to the compass_set_learning_param can be defined by observing the ML curve growth over itrations.  Because we start with a random start value for model parameters, there is a chance to see a non-increasing ML on early iterations of the algorithm. One approach would be to estimate a static version of the model, e.g. through a standard GLM with the same terms as the state-space model. The arguments of Param can then be manually set as described above. Note that changes in Param must be done after calling compass_create_state_space function. Using a reasonable starting point, we might reduce number of EM iterations. Furthermore, we might get a more well-behaved ML curve as well. |
| Eyn | A vector of length $K$ which returns the expected value of the continuous process at each time index.  In the Normal observation process, this is the deterministic part of the observation process estimated by the model – or simply the mean estimate of the process at each time index. This is defined by: $y_{c}\left( k \right)={CT}_{k}X_{k\vert K}\left( k \right)+{DT}_{k}$ In the Gamma observation process, this is equal to the mean of the observation process at each sample. Check **Reference 1** for further information. This is defined by: $y_{c}\left( k \right)={exp(CT}_{k}X_{k\vert K}\left( k \right)+{DT}_{k})$ Note that parameters in ${CT}_{k}$ and ${DT}_{k}$ are updated paramaters. |
| Eyb | A vector of length $K$ which returns the expected probability of the correct choice at each time index.  The expected probability at each time index is defined by: ${P(y}_{d}\left( k \right)==1)={{exp(ET}_{k}X_{k\vert K}\left( k \right)+{FT}_{k})}/{(1+{exp(ET}_{k}X_{k\vert K}\left( k \right)+{FT}_{k}))}$ Note that parameters in ${ET}_{k}$ and ${FT}_{k}$ are updated parameters. |
| rYn | Updated Yn Vector. It has the same length as Yn.  Elements of the vector corresponding to censored data are replaced by an imputation technique. Check compass_sampling for further information. |
| rYb | Updated Yb Vector. It has the same length as Yb.  Elements of the vector corresponding to censored data are replaced by an imputation technique. Check compass_sampling for further information |

**Example**

Here, we assume input elements are properly loaded and assigned.

%% Load sample data

% data: Ii, Ini, Iin, Yk_1 , Yk

load('MSIT_1.mat');

%% First step, build state space model - create behavioral model

Param = compass_create_state_space(1,0,5,0,[1;1],[1 2],[0 1],[],[]);

%% Second step, define learning parameters

Iter = 100;

Param = compass_set_learning_param(Param,Iter,0,1,1,1,1,1,1,2,1);

%% Third step, define censored time threshold if it is necessary

censor_time = 1;

Param= compass_set_censor_threshold_proc_mode (Param,censor_time);

%% Fourth step, format the data (all points are observed)

% In

In = [Ini Iin ones(length(Yk),1) Ii Yk_1];

% all data points are valid

valid = ones(length(Yk),1);

%% fifth Step, EM Algorithm

[XSmt,SSmt,Param,XPos,SPos,ML,Yp]=compass_em([2 0],[],In,[],Yk,[],Param,valid);

**compass_filtering**

This function runs the filtering (causal state estimation) algorithm, given the most recent observed signals. This function is appropriate for real-time filtering, when the observed signals are updated trial-by-trial. This function comes in handy when we want to run the toolbox in real time to assess changes in underlying state variable. The function gets the current observation signals plus an estimate of the state variable from the previous trial to estimate the current value of the state variables. The Param structure normally comes from compass_em, which should be run on a training dataset. That is, the best way to use this function is to have the subject perform a block of behavioral trials, run compass_em, and then use the results of that analysis to perform real-time behavior analysis.

The present version of compass_filtering can only run on scalar values of the continuous/discrete process, i.e., a single value such as reaction time and a single value such as correct/incorrect choice.

**Syntax**

[XPos,SPos,YP,YB]=compass_filtering(DISTR,Uk,In,Ib,Yn,Yb,Param,obs_valid,XPos0,SPos0)

**Description**

[XPos,SPos,YP,YB]=compass_filtering(DISTR,Uk,In,Ib,Yn,Yb,Param,obs_valid,XPos0,SPos0)

It runs an on-line filter algorithm on each new trial – or time-index – of the data.

**Input Arguments**

| DISTR | Check compass_em for further information |
| --- | --- |
| Uk | A vector of size $1\times nUk$ which defines the current values of the input to the state-transition model.  Note that compass_filtering will be called per trial, i.e. only one trial worth of input should be provided.  For further information, check compass_em function  **Example:** $Uk=1$, it means the current input to the state-transition process is a scalar value of 1. |
| In | A vector of size $1\times nIn$ which defines the current value of the input to the continuous process of the model.  Note that compass_filtering will be called per trial.  For further information, check compass_em function  **Example:** $In=rand(1,5)$, it means a random input with 5 elements at each trial. |
| Ib | A vector of size $1\times nIb$ which defines current value of the input to the discrete process of the model.  Note that compass_filtering will be called per trial.  For further information, check compass_em function  **Example:** $Ib=rand(1,5)$, it means a random input with 5 elements on the current trial. |
| Yn | A scalar value; either a real value or NaN.  For further information, check compass_em function definition.  **Example:** $Yn=rand$, it means a random input for current observation of the continuous signal.  Note that in practice, this is the observed behavior or an equivalent signal. |
| Yb | A scalar value either 0, 1, or NaN.  For further information, check compass_em function definition.  **Example:** $Yb=1$, it means a correct response for the current trial of the discrete signal.  Note that in practice, this is the observed behavior or an equivalent signal. |
| Param | Updated Param structure. In practice, this is the output of the compass_em run over a training dataset.  A structure array consisting both the state-space and behavioral model parameters plus EM learning setup.  Check Param in compass_create_state_space and compass_set_learning_param for a more complete description. |
| XPos0 | The mean value of the state variable filter on the previous trial.  The $XPos0$ is a vector of length $nx$. |
| SPos0 | The covariance matrix of the state variable filter on the previous trial.  The $sPos0$ is a matrix of size $nx\times nx$. |
| obs_valid | A scalar variable with values of either 0, 1, or 2.  A value of 1 means a valid observed signal/s.  A value of 2 means a censored observed signal/s.  A value of 0 means a missing observed signal/s.  For further information, check the compass_em function definition. |

**Output Arguments**

| Xpos | The mean value of the state variable filter on the current trial.  Note that it is a function of the previous trial state estimate plus the new observed signals.  The $XPos$ is a vector of length $nx$. |
| --- | --- |
| Spos | The covariance matrix of the state variable filter on the current trial.  Note that, it is a function of the previous trial state estimate plus the new observed signals.  The $sPos$ is a matrix of size $nx\times nx$. |
| YP | A scalar value which returns the expected value of the continuous process on the current trial.  It is a function of the current estimate of the state variable and the trial input variables – $Uk$ and $In$. |
| YB | A scalar value which returns the expected probability of correct decision on the current trial.  It is a function of the current estimate of the state variable and the trial input variables – $Uk$ and $Ib$. |

**Example**

Here, we assume the Param is the output of the compass_em algorithm ran over the training dataset. compass_filtering is called twice, once for the first trial and then on the second trial of the task. Note how we set our initial estimates of the mean and covariance of the state variables. If we had training data, we could equally set these based on the end of that training data. We can put the following procedure in a loop, where it would be called on each subsequent trial.

% Set initial state and variance; seed values are required for the algorithm

XPos0=[0;0];

SPos0=[10 0;0 10];

% first observation (note In,Ib are vectors of length 1xnIn, 1xnIb and similarly Uk)

% XPos0 and SPos0 are the estimate of state mean and variance from previous one

% we assume data is valid

[XPos0,SPos0]=compass_filtering([1 0],[],In(1,:),[],Yn(1),[],Param,1,XPos0,SPos0);

% second observation

[XPos0,SPos0]=compass_filtering([1 0],[],In(2,:),[],Yn(2),[],Param,1,XPos0,SPos0);

**compass_sampling**

This function is called to generate a sample of continuous and discrete signals on the censored trials. This function is used in the imputation technique used in compass_em on the censored trials. The current version of compass_em fetches a sample of data given the current update of the observation distribution on the censored trials, then runs the EM algorithm using the imputed sample.

**Syntax**

[Yn,Yb]=compass_sampling(DISTR,Cut_Time,Uk,In,Ib,Param,XPos0,SPos0)

**Description**

[Yn,Yb]=compass_sampling(DISTR,Cut_Time,Uk,In,Ib,Param,XPos0,SPos0)

It generates a sample (Yn,Yb) for continuous and discrete observation signals, given the current inputs of the model (Uk,In,Ib) and a previous estimate of the state-variables (XPos0,SPos0). The Cut_Time variable defines the censoring threshold; note that censoring criteria is only conditioned on the value of the continuous signal. Param keeps the updated set of the model parameters.

**Input Arguments**

| DISTR | It is similar to DISTR defined in compass_filtering and compass_em . Check compass_em or compass_filtering for further information |
| --- | --- |
| Cut_Time | It is a scalar value which define the censoring threshold.  A copy of this value is carried by Param as well. |
| Uk | It is similar to Uk defined in compass_filtering. Check compass_filtering for further information. |
| In | It is similar to In defined in compass_filtering. Check compass_filtering for further information |
| Ib | It is similar to Ib defined in compass_filtering. Check compass_filtering for further information  . |
| Param | It is similar to Param defined in compass_filtering. Check compass_filtering for further information |
| XPos0 | It is similar to XPos0 defined in compass_filtering. Check compass_filtering for further information |
| SPos0 | It is similar to SPos0 defined in compass_filtering. Check compass_filtering for further information |

**Output Arguments**

| Yn | A scalar variable returning a sample of the continuous signal over the censored threshold. |
| --- | --- |
| Yb | A scalar discrete variable returning a sample of the discrete signal over the censored threshold.  Yb can be either 0 or 1. |

**Example**

Here, the compass_sampling is called inside the compass_em function to create sample data to impute the state for the censored trials.

if obs_valid(k)==2

[tYP,tYB]=compass_sampling(DISTR,censor_time,Uk(k,:),In(k,:),Ib(k,:),Param,XPre{k},SPre{k});

if DISTR(1)>0 Yn(k)=tYP; end;

if DISTR(2)==1 Yb(k)=tYB; end;

end

**compass_deviance**

This function can be called to estimate the deviance measure over both continuous and discrete parts of the observations. Deviance is an extension of the concept of mean squared error, which is used in the linear models with a normal observation process. This function requires compass_em's output argument and it can be called after that. The deviance measure can be used to compare goodness-of-fit across different models, and to identify the model which gives a better fit.

**Syntax**

[DEV_C,DEV_D]=compass_deviance(DISTR,In,Ib,Yn,Yb,Param,obs_valid,XSmt,SSmt)

**Description**

[DEV_C,DEV_D]=compass_deviance(DISTR,In,Ib,Yn,Yb,Param,obs_valid,XSmt,SSmt)

It generates deviance measures (DEV_C, DEV_D) for continuous and discrete observation signals, given the smoother estimate of the state variables plus other inputs to the model (In,Ib). Param keeps the updated set of the model parameters. obs_valid carries information of each data point: whether it is observed, censored or dropped (missing at random). Note that deviance calls compass_sampling on censored data points and ignores missing at random data points in calculating deviance. We do not require Uk as an input argument. Uk determines the state transition process, and we have the state estimation through XSmt and SSmt. Thus there is no need for Uk.

**Input Arguments**

| DISTR | It is similar to DISTR defined in compass_filtering and compass_em . Check compass_em or compass_filtering for further information |
| --- | --- |
| Cut_Time | It is a scalar value which define the censoring threshold.  A copy of this value is carried by Param as well. |
| In | It is similar to In defined in compass_filtering. Check compass_filtering for further information |
| Ib | It is similar to Ib defined in compass_filtering. Check compass_filtering for further information  . |
| Param | It is similar to Param defined in compass_filtering. Check compass_filtering for further information |
| XSmt | It is similar to XSmt defined in compass_em output argument. Check compass_em for further information |
| SSmt | It is similar to SSmt defined in compass_em output argument. Check compass_em for further information |

**Output Arguments**

| DEV_C | A scalar variable returning the deviance measure for the continuous observation process. This is a valid value only when DISTR is set for continuous observation. |
| --- | --- |
| DEV_CD | A scalar variable returning the deviance measure for discrete observation process. This is a valid value only when DISTR is set for discrete observation. |

**Example**

Here, compass_deviance is called after compass_em to estimate the deviance measures for both discrete and continuous parts.

%% Run learning with a mixture of normal & binary

% note, we ran compass_em once with the default setting for parameters and then ran it a second time with

% the resulting learned parameters.

[XSmt,SSmt,Param,XPos,SPos,ML,YP,YB]=compass_em([1 1],Uk,In,Ib,Yn,Yb,Param,Valid);

**%% Deviance analysis**

[DEV_C,DEV_D]= compass_deviance([1 1],In,Ib,Yn,Yb,Param,Valid,XSmt,SSmt);

**compass_param_covariance_info**

This function returns the covariance matrix estimates for model free parameters. The covariance estimate can be used to identify the confidence interval of the model parameters. This function is called after compass_em and it is used to examine significance of the model being built for the data. The covariance estimate of the model parameters can be used to refine the model structure and to check whether each element is significant or not. Parameters whose variance (on the diagonal) implies a distribution that does not include zero could be considered significant. Parameters with a wide variance (distribution covers zero) should be considered for removal from the model. Covariance analysis is similar to GLM covariance analysis (Reference 7 – Ch10). It can be used to assess goodness of the model and to refine the model structure.

**Syntax**

[COV_X,COV_C,COV_D]=compass_param_covariance_info(DISTR,Uk,In,Ib,Yn,Yb,Param,obs_valid,XSmt,SSmt)

**Description**

[COV_X,COV_C,COV_D]=compass_param_covariance_info(DISTR,Uk,In,Ib,Yn,Yb,Param,obs_valid,XSmt,SSmt)

This function gets all the input being passed to compass_em and the filter result returned by compass_em: XSmt and SSmt. Note that in this function, we assume that all model parameters are known, and that we have an optimal estimate of the state variables. In other words, we use maximum likelihood estimates of the model parameters and state variables here. It returns the covariance estimates for the state transition process free parameters and similarly for free parameters of the continuous and discrete observation processes. The function returns three structures:

1. COV_X: which carries covariance matrix estimate for the parameters of the state-transition process. Note that we estimate the noise variance as well.
2. COV_C: which carries covariance matrix estimates for parameters of the continuous observation process. We estimate the noise variance or dispersion depending on the distribution.
3. COV_D: which carries covariance matrix estimates for parameters of the discrete observation process.

**Input Arguments**

| DISTR | A 2 element vector which defines the type of the observed signals and their corresponding distributions.  **DISTR=[1 0]** means there is only continuous observation signal and the proper distribution for the signal is Normal. Note that the input argument Yn carries the continuous signal.  **DISTR=[2 0]** means there is only continuous observation signal and the proper distribution of the signal is Gamma. Note that the input argument Yn carries the continuous signal.  **DISTR=[0 1]** means there is only discrete observation – a Bernoulli observation process.  Note that the input argument Yb carries the continuous signal.  **DISTR=[1 1]** means there are both continuous and discrete observation signals and the proper distribution for the signal is a mixture of Normal and Bernoulli distributions. Note that the input arguments Yn and Yb carry the mixed signals.  **DISTR=[2 1]** means there are both continuous and discrete observation signals and the proper distribution for the signal is a mixture of Gamma and Bernoulli distributions. Note that the input arguments Yn and Yb carry the mixed signals.  **Example:** $DISTR =[1 0]$, it means the observed signal includes only continuous observation. Note that we might define the state-space model with both continuous and discrete observations; the EM algorithm might process either continuous or discrete parts of the process given the $DISTR$ elements. |
| --- | --- |
| Uk | A matrix of size $K\times nUk$ which defines the input to the state-transition model – $K$ is the number of observations, and $nUk$ is number of elements per each time index. The state-transition process is defined by: $X\left( k+1 \right)= A X\left( k \right)+ B U\left( k \right)+W$ **Example:** $Uk=ones(100,1)$, it mean that input is a fixed value of 1 at each time index. Note that it is assumed that $K$ is equal to 100, which is the length of the observation. |
| In | A matrix of size $K\times nIn$ which defines the input to the continuous observation process. For further information about how columns of $In$ are utilized in the continuous observation process, check the compass_create_state_space function.  **Example:** $In=randn(100,5)$; it means a random input with 5 elements at each time index is passed to the continuous observation process. Note that it is assumed that $K$ is equal to 100, which is the length of the observation.  Practically, the $In$ elements could be indicator functions of the task factors or even continuous values, including history terms.  Note that $Uk$, $In$, and $Ib$ share the same number of rows. |
| Ib | A matrix of size $K\times nIb$ which defines the input to the discrete observation process. It is equivalent to $In$ of the continuous part.  **Example:** $Ib=randn(100,5)$; it means a random input with 5 elements at each time index is passed to the discrete observation process. Note that it is assumed that $K$ is equal to 100, which is the length of the observation.  Practically, the $Ib$ elements could be indicator functions of the task factors or even continuous values, including history terms.  Note that $Uk$, $In$, and $Ib$ share the same number of rows. |
| Yn | A vector of size $K\times1$ which carries the continuous observation signal - $K$ is the length of observation process.  Values of Yn can be real values or NaN. Note that for a NaN observation signal, the corresponding obs_valid must be either 0 or 2. Check obs_valid for further information.  Note that for an observation process with a Normal distribution, $DISTR=[1 X]$, the Yn elements might be real values. For the observation process with a Gamma distribution, $DISTR=[2 X]$, the Yn elements might be positive real-valued.  **Example:** $Yn=rand(100,1)$; it means a positive random input with 100 rows.  Note that $Uk$, $In$, $Ib$, and $Y_{n}$ share the same number of rows. |
| Yb | A vector of size $K\times1$ which carries the discrete observation signal - $K$ is the length of observation process.  Values of Yb are 0, 1, or NaN. Note that for a NaN observation signal, the corresponding obs_valid must be either 0 or 2. Check obs_valid for further information.  Value of 0 means an incorrect response, and a value of 1 means a correct response.  **Example:** $Yb=randi(2,[100 1])-1$, which means a vector with random values of 0 or 1 at each row.  Note that $Uk$, $In$, $Ib$, $Yn$, and $Yb$ share the same number of rows. |
| Param | A structure array consisting of both the state-space model structure plus EM learning parameters.  Check Param in compass_create_state_space and compass_set_learning_param for a more complete description. |
| obs_valid | A vector of length K with elements of 0, 1, and 2.  An element with value 1 means the observed signal is valid.  An element with value 0 means the observation is missing.  An element with value 2 means the observation is censored. The censoring threshold is defined by the Param.censor_time.  Note that a NaN value in either Yn or Yb must be accompanied by a value 0 or 2 in obs_valid, otherwise the EM algorithm fails to have a correct estimate of the model parameters and state variables.  Note that obs_valid determines how the EM algorithm treats each row of the observed signal in running either E- or M-step of the EM process. Thus, setting correct values of the obs_valid has a significant importance in running the model correctly.  **Example:** $obs\_valid= ones(100,1)$, it means all elements of the observed signals are filled with valid values.  Note that $Uk$, $In$, $Ib$, $Yn$, $Yb$, and $obs\_valid$ share the same number of rows. |
| XSmt | It is similar to XSmt defined in compass_em output argument. Check compass_em for further information |
| SSmt | It is similar to SSmt defined in compass_em output argument. Check compass_em for further information |

**Output Arguments**

| COV_X | This structure returns covariance matrices for parameters of the state-transition process.  r = 1:nx  COV_X{r}.A: covariance matrix for matrix A, r-th row  COV_X{r}.AB:cross covariance matrix for matrix A & B, r-th row  COV_X{r}.B: cross covariance matrix for matrix B, r-th row  COV_X{r}.W: variance estimate for r-th state variable model  COV_X{r}.SE_W: std error for r-th state variable model  If we have nx state variables, each of these nx structures will address the covariance estimate for the corresponding state variable. |
| --- | --- |
| COV_C | This structure returns covariance matrices for parameters of the continuous observation process  COV_C.C: covariance matrix for C – check observation model definition  COV_C.SE_C: std error for C – check observation model definition  COV_C.CD: covariance matrix for C & D parameters – check observation model definition  COV_C.D: covariance matrix for D parameters – check observation model definition  COV_C.SE_D: std error for D – check observation model definition  COV_C.V: variance for observation process noise |
| COV_D | This structure returns covariance matrices for parameters of the discrete observation process  COV_D.E: covariance matrix for E – check observation model definition  COV_D.SE_E: std error for E – check observation model definition  COV_D.EF: covariance matrix for E & F – check observation model definition  COV_D.F: covariance matrix for F – check observation model definition  COV_D.SE_F: std error for F – check observation model definition |

Note that the function also returns covariance matrices estimates for model fixed parameters – the subset of parameters being excluded from training step. These are found in the corresponding rows of their matrices. This will be helpful for model modification and provides a better sense about whether the choice of those parameters was reasonable.

**Example**

Here, we call compass_param_covariance_info after compass_em. We can check output arguments of the function to find significant components.

%% Run learning with a mixture of normal & binary

% note, we ran compass_em once with default setting for parameters and then

% set parameters close to estimated one.

[XSmt,SSmt,Param,XPos,SPos,ML,YP,YB]=compass_em([1 1],Uk,In,Ib,Yn,Yb,Param,Valid);

**%% Covariance analysis**

**[COV_X,COV_C,COV_D]=compass_param_covariance_info([1 1],Uk,In,Ib,Yn,Yb,Param,Valid,XSmt,SSmt);**
